# Long wavelength single photon like driven photolysis via triplet triplet annihilation

**DOI:** 10.1038/s41467-020-20326-6

**Published:** 2021-01-05

**Authors:** Ling Huang, Le Zeng, Yongzhi Chen, Nuo Yu, Lei Wang, Kai Huang, Yang Zhao, Gang Han

**Affiliations:** Department of Biochemistry and Molecular Pharmacology, University of Massachusetts Medical School, Worcester, MA USA

**Keywords:** Light harvesting, Synthesis and processing

## Abstract

Photolysis has enabled the occurrence of numerous discoveries in chemistry, drug discovery and biology. However, there is a dearth of efficient long wavelength light mediated photolysis. Here, we report general and efficient long wavelength single photon method for a wide array of photolytic molecules via triplet-triplet annihilation photolysis. This method is versatile and “LEGO”-like. The light partners (the photosensitizers and the photolytic molecules) can be energetically matched to adapt to an extensive range of electromagnetic spectrum wavelengths and the diversified chemical structures of photoremovable protecting groups, photolabile linkages, as well as a broad array of targeted molecules. Compared to the existing photolysis methods, our strategy of triplet-triplet annihilation photolysis not only exhibits superior reaction yields, but also resolves the photodamage problem, regardless of whether they are single photon or multiple photon associated. Furthermore, the biological promise of this “LEGO” system was illustrated via developing ambient air-stable nanoparticles capable of triplet-triplet annihilation photolysis.

## Abstract (web summary)

The use of short wavelength light in photolysis applications in chemistry and biology is limited by photolytic reaction yields, photodamage and photobleaching. Here, the authors report a general long wavelength single photon driven photolysis method using a triplet-triplet annihilation process.

## Introduction

**Fig. 1:**
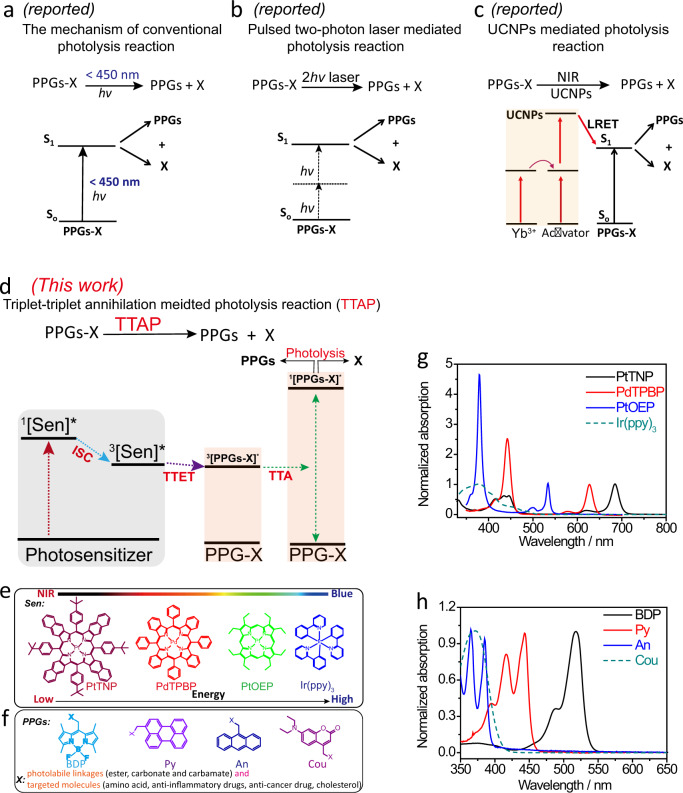
The mechanism of reported state-of-the-art photolytic reactions and the TTAP mechanism in this work. **a** The reported mechanism of conventional photolytic mechanism. *hv*: single photon. **b** Pulsed two photon laser strategy mechanism for photolytic reaction. 2 *hv*: two photon. **c** The reported lanthanide ion-doped upconversion nanoparticles (UCNPs)-mediated photolytic reaction. LRET luminescence resonance energy transfer. **d** The mechanism of triplet–triplet annihilation-mediated photolysis (TTAP, this work). ISC intersystem crossing, TTET triplet–triplet energy transfer, TTA triplet–triplet annihilation, ^1^[Sen]^*^ the singlet excited state of the photosensitizer, ^3^[Sen]^*^ the triplet excited state of the photosensitizer, PPGs-X a photolytic molecule, PPGs photoremovable protecting groups (BDP, Py, An, and Cou), X targeted molecules (amino acids, anti-inflammatory drugs, anti-cancer drug, cholesterol), and photolabile linkage (ester bond, carbonate, carbamate). **e** Molecular structures of photosensitizers, including PtTNP, PdTPBP, PtOEP, and Ir(ppy)_3_. **f** Molecular structures of PPGs, including BDP, Py, An, and Cou moieties. **g** The normalized UV–vis absorption spectra of photosensitizers. **h** The normalized UV–vis absorption spectra of PPGs.

Photolysis is a chemical reaction in which a chemical compound is broken down by light to allow for non-invasive control of the release and activation of targeted molecules. Due to the unique and precise spatiotemporal controllability, the use of photolysis has been a powerful approach that has vast applications from organic synthesis, drug discovery to numerous biological areas, such as developmental biology, neuromodulation, as well as cancer treatments. In general, a photolytic molecule (PPG-X) consists of three key components: a target molecule, a photolabile linkage and a photoremovable protecting group (PPG). In photolysis, PPG absorbs high-energy photons and then transitions to an excited state, causing photolabile linkage to break down and the subsequent release of targeted molecules. Unfortunately, most of the existing PPGs, such as coumarin (Cou), anthracene (An) and perylene (Py), boron-dipyrromethene (BDP) groups only respond to high-energy short wavelength light excitation for subsequent single photon photolytic reactions (Fig. 1a)^1^. However, the use of short wavelength light for photolysis has inherent drawbacks. For instance, the shallow penetration of such short wavelength light through colored reaction solvents or media leads to poor photolytic reaction yields, especially in large-scale chemical reactions^2^. In addition, short wavelength photons cause the rapid photodamage and photobleaching of PPGs^1,2^. Moreover, in regard to photolysis applications in biology, the term “photouncaging” has been coined to describe the technique of using light to remove the PPGs and activate biological compounds so as to noninvasively probe different biological processes, neuronal connections, as well as to develop disease treatments^3^. However, photouncaging using conventional short-wavelength light also comes along with a series of serious problems, such as inevitable phototoxicity and shallow tissue penetration depths^4^. Ideally, these problems can be overcome by the use of lower energy long wavelength light (including far red light and near infrared light), which has much higher penetration depth through various media and biological tissue^5^.

To date, there are only a pair of state-of-the-art long wavelength activation photolytic methods in the reported literature. For example, the femtosecond pulsed two-photon laser can be used to remove PPGs^6^. However, this process is quite inadequate for photolysis, as the two-photon absorption cross-sectional area of PPGs is quite weak and the reactions can only take place in the tiny area of the laser focal point (Fig. 1b)^1^. Meanwhile, lanthanide ion-doped upconversion nanoparticles (UCNPs)-assisted photolysis have emerged as an appealing method^7^. Yet, owing to low absorption and emission cross-sections, the lanthanide ion-doped UCNPs typically suffer from the need for high power light excitation (10^1^–10^4^ W cm^−2^) and inherently low quantum yields^8,9^. In addition, upconversion luminescence resonance energy transfer (LRET) from inorganic UCNPs to conjugated PPGs is generally inefficient (Fig. 1c).

In this study, we report on a highly effective and general long wavelength single photon-driven photolysis method via triplet–triplet annihilation (TTA). The detailed mechanism for our TTA-mediated photolysis (TTAP) is delineated in Fig. 1d. As depicted, photosensitizers (Sen) can absorb low-energy long wavelength photons, reach their singlet excited state (^1^[Sen]^*^) and subsequently populate their long-lived triplet excited state (^3^[Sen]^*^) through rapid intersystem crossing (ISC). The lifetime of this photosensitizer triplet excited state is long enough to allow collisions of the targeted PPGs-X to occur. The energy of ^3^[Sen]^*^ can thus be efficiently transferred to PPGs-X. Consequently, the TTA of ^1^[PPGs-X]^*^ drives photolytic reactions to take place, thus breaking down the photolabile linkage and releasing and activating the targeted molecules. Because of the much greater light absorption of photosensitizers than PPGs themselves and the highly efficient triplet–triplet energy transfer (TTET) from photosensitizers to PPGs-X, we envision our long wavelength single photon-driven TTAP method to outperform the existing short-wavelength single photon direct activation and the above-mentioned long wavelength activation methods.

## Results

### The general rules to design effective TTAP “LEGO” systems

In particular, in the proposed TTAP process, there are two general rules to design effective TTAP “LEGO” systems. Firstly, the PPGs should have a lower laying triplet excited state than that of the triplet excited state of the photosensitizer (^3^[PPGs]^*^ < ^3^[Sen]^*^), allowing the efficient TTET process to occur from Sen to PPGs. Secondly, the doubled energy of the triplet excited state of the PPGs should be higher than that of the singlet excited state of the PPGs (2×^3^[PPGs]^*^ > ^1^[PPGs]^*^), enabling the ultimate TTA to take place (Fig. 1d)^10–12^.

### Versatile, high-performing, and “LEGO”-like TTAP light partners

To verify our hypothesis, we began our TTAP investigation with a widely used PPG from an ultraviolet light absorbing Cou group (*T*_1_ = 2.18 eV) (Supplementary Table 1)^13^. Here, due to its broad presence in pharmaceuticals and natural products, in conjunction with such PPG from Cou group, the ester bond was tested to see the feasibility of its being used for photolabile linkage to protect carboxylate (acetic acid) in compound **1** (see below). After considering the energetic requirements of the TTAP, Ir(ppy)_3_ (*T*_1_ = 2.4 eV) (Supplementary Table 1) was chosen as the initial coupling photosensitizer partner (Supplementary Fig. 2 and Supplementary Tables 2 and 3)^14^. The TTET process from Ir(ppy)_3_ to compound **1** was studied via Stern−Volmer photoluminescence quenching assays under oxygen-free conditions. When we titrated compound **1** into the Ir(ppy)_3_ solution, the phosphorescence decreased dramatically (Supplementary Fig. 3). The respective Stern−Volmer constants (*k*_sv_) were calculated to be 3.13 × 10^3^ M^−1^. Next, when combining the Ir(ppy)_3_ with compound **1**, we observed the photolytic product in the yield of 73% under the blue light illumination at characteristic absorption of Ir(ppy)_3_ (476 nm, LED, 20 mW cm^−2^). In contrast, in the absence of Ir(ppy)_3_, no photolytic product was observed under such blue light irradiation, since the PPG of the Cou group requires ~360 nm ultraviolet light direct activation (Supplementary Table 4). This experiment clearly validated the feasibility of our proposed TTAP concept.

Inspired by the above-mentioned TTAP experimental results, we continued to examine whether this TTAP concept is general enough to be adapted to a wide spectrum of electron-magnetic wavelengths (Fig. 1e–h). To do so, we tested a series of long wavelength absorbing sensitizers, ranging from green (PtOEP), far-red (PdTPBP) to near infrared light (PtTNP) (Fig. 1e and g), as well as a broad array of PPGs (An, Py, and BDP) (Fig. 1f and h). Through extensive analysis of the photophysical properties (Supplementary Tables 2 and 3), we identified a family of potential TTAP light partners based on the energetic match among these sensitizers and PPGs. These new TTAP pairs are: (1) PtOEP: An; (2) PtOEP: Py; (3) PdTPBP:Py; (4) PdTPBP:BDP; (5) PtTNP:Py; and (6) PtTNP:BDP (Supplementary Tables 2 and 3, and Supplementary Fig. 2). To further support our theoretical spectrum analysis, we then tested the Stern−Volmer constants (*k*_sv_) (Supplementary Figs. 5–15 and Supplementary Tables 5–11) in regard to all of these selected combinations. As shown in Supplementary Table 1, compound **2** with PPG of An (*T*_1_ = 1.77 eV)^15^ and compound **8** with PPG of perylene (*T*_1_ = 1.52 eV)^16^ demonstrate significant quenching of the photoluminescence of green light-absorbing photosensitizer of PtOEP (*T*_1_ = 1.92 eV) (Supplementary Figs. 5 and 7). Meanwhile, for far-red light absorbing photosensitizer PdTPBP, compound **7** with PPG of BDP (*T*_1_ = 1.49 eV) and compound **8** with PPG of perylene exhibited obvious quenching effects on the photoluminescence of PdTPBP (*T*_1_ = 1.55 eV) (Supplementary Figs. 9 and 10). Moreover, regarding NIR light absorbing photosensitizer PtTNP, compounds **7** and **8** also show quenching effect on its photoluminescence (*T*_1_ = 1.43 eV) (Supplementary Fig. 11).

Moreover, the TTA-upconversion (TTA-UC) properties were studied for these pairs. The TTA-UC spectra of these pairs were measured in deaerated toluene. The upconversion quantum yields (Φ_UC_) were calculated based on the established method in the literature^12^. Supplementary Figs. 6 and 8 are the TTA-UC spectra of green light-activated TTA-pairs of PtOEP: An (compound **2**) and PtOEP:Py (compound **8**) under 530 nm (20 mW cm^−2^) light illumination. The upconversion quantum yields are determined to be 8.7% for PtOEP: An (compound **2**) and 9.5% for PtOEP: Py (compound **8**). Supplementary Fig. 15a and b are TTA-UC spectra of the red light-activated TTA pairs of PdTPBP:Py (compound **8**) and PdTPBP:BDP (compound **7**) under 650 nm (20 mW cm^−2^) light illumination. The upconversion quantum yields are determined to be 5.8% for PdTPBP:Py (compound **8**) and 8.4% for PdTPBP:BDP (compound **7**). Supplementary Fig. 15c and d are the TTA-UC spectra of the NIR light-activated TTA pairs of PtTNP:Py (compound **8**) and PtTNP:BDP (compound **7**) under 650 nm (20 mW cm^−2^) light illumination. The upconversion quantum yields are determined to be 0.05% for PtTNP:Py (compound **8**) and 0.3% for PtTNP:BDP (compound **7**). Furthermore, the upconversion emission for the TTA-pairs is observed to be power-dependent (Supplementary Figs. 12–14). The threshold intensities (*I*_th_) are determined to be 25.4 mW cm^−2^ for PtOEP:An (compound **2**), 21.8 mW cm^−2^ for PtOEP:Py (compound **8**), 48.1 mW cm^−2^ for PdTPBP:Py (compound **8**), 46.7 mW cm^−2^ for PdTPBP:BDP (compound **7**), 94.5 mW cm^−2^ for PtTNP:Py (compound **8**), and 119.8 mW cm^−2^ for PtTNP:BDP (compound **7**), respectively. Below the respective threshold intensity (*I*_th_), the upconversion intensity (*I*_UC_) and excitation power intensity (*I*_ex_) are in a quadratic relationship. When the *I*_ex_ exceeds the threshold intensity, the relationship between *I*_UC_ and *I*_ex_ becomes linear (Supplementary Figs. 12–14).

**Fig. 2:**
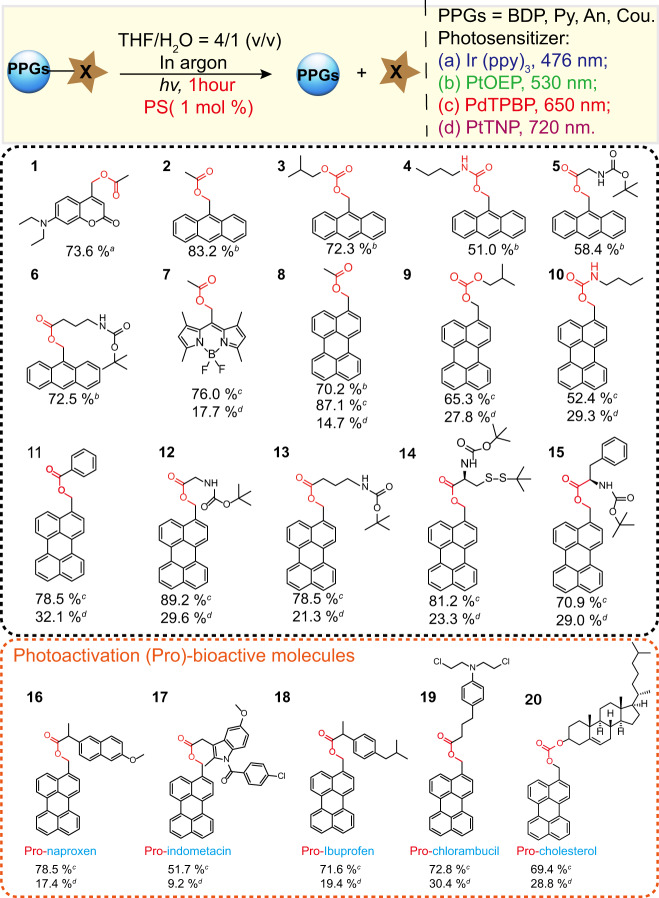
Triplet–triplet annihilation-mediated photolytic reactions under diverse low power long wavelength LED irradiation. The superscripts (a–d) next to the reaction yields represent the sensitizers used and their respective operation light wavelength.

Next, we measured the photolysis yields of the above-mentioned TTAP “LEGO” systems (Fig. 2). Specifically, we first tested the two green light-activating TTAP pairs, including PtOEP:An (compound **2**) and PtOEP:Py (compound **8**). We found that their photolytic yield is outstanding: 83% for An containing compound **2** and 61% for Py containing compound **8**, respectively, under 20 mW cm^-2^ 532 nm LED light illumination (Supplementary Tables 5 and 6). In a similar manner, we also observed significant photolytic yield at 76% and 87% for our far-red light activating TTAP pairs (PdTPBP:compound **7**, PdTPBP:compound **8**), respectively, under 20 mW cm^−2^ 650 nm LED light irradiation (Supplementary Tables 7 and 8). Moreover, we tested our NIR light-activating TTAP candidates (PtTNP:compound **8**, PtTNP:compound **7**). Notable photolytic yield at 14.7% and 17.7%, respectively, were also observed under 1 h illumination of 20 mW cm^−2^ 720 nm NIR LED (Supplementary Tables 9 and 10).

It is exciting that we further found that such low-energy long wavelength activation can indeed even surpass the traditionally high-energy short-wavelength light needed direct activation. This is the case because of the advantages of the TTAP in regard of the intense long wavelength light absorbance of photosensitizer, the highly effective TTAP mechanism and the reduced photodamage on the PPGs-X. For example, in the absence of the photosensitizer partner, the traditional photolytic reaction on compound **8** with blue light (445 nm, 20 mW cm^−2^) only leads to a photolytic yield of 59% (Supplementary Table 11). In contrast, our TTAP “LEGO” system (PdTPBP: compound **8**) is able to effectively improve the photolytic yield to 87.1% for compound **8** under a far-red LED (20 mW cm^−2^, 650 nm). Furthermore, the photolytic quantum efficiency (QE) was calculated (see details in “Methods” section). The resulted QE value gives an overall evaluation with respect to photolysis reactions, as it considers both the photolysis quantum yield of PPGs and the absorbance of sensitizers, TTET and TTA quantum yields (Supplementary Table 12). As a result, for the PPG of An (compound **2**), in conjunction with PtOEP, QE_(TTAP)_ is calculated to be 1255. For the PtOEP and Py (compound **8**), the QE_(TTAP)_ is calculated to be 708. Moreover, in conjunction with PdTPBP, the QE_(TTAP)_ is calculated to be 679 for the PPG of Py (compound **8**) and QE_(TTAP)_ is 12.3 for BDP (compound **7**), respectively. In addition, the QE_(TTAP)_ is 7.1 for the PPG of Py (compound **8**) and QE_(TTAP)_ is 0.53 for the PPG of BDP in conjunction with PtTNP, respectively.

Compared to conventionally used protecting groups in peptide synthesis that are involved with harsh chemical conditions such as acid/base-sensitive or redox-sensitive groups, PPGs have been emerging as a traceless and green alternative to allow “reagent-free” deprotection under light illumination^17^. Thus, herein we expanded our TTAP concept to a variety of amino acids such as glycine, cysteine, and phenylalanine (Fig. 2). As a result, we observed excellent low power long wavelength light-driven single photon-mediated deprotection for these amino acids containing target molecules. For PPGs of An for protecting glycine (compound **5**), we observed the photolytic yield is 58.4% in conjunction with PtOEP under green LED illumination. For PPGs of Py for protecting glycine (compound **12**), in conjunction with PdTPBP and PtTNP, the photolytic yield is 89.2%, 29.6%, respectively. For PPGs of Py for protecting cysteine (compound **14**), when PdTPBP and PtTNP are used as photosensitizers, the photolysis yields are 81.2% and 23.3%, respectively. In addition, for PPGs of Py for protecting phenylalanine, with PdTPBP and PtTNP as photosensitizers, the photolytic yields are 70.9% and 29.0%, respectively. These results demonstrated the great potential of our TTAP technology in a wide array of applications in relation to functional photocaged amino acids and peptide synthesis. (Fig. 2)

Having identified our TTAP “LEGO” systems enable photolytic ester bonds, we then turned our attention to evaluate the scope of this method with other typically used photolabile linkages of carbonate (compound **3**, compound **9**) and carbamate (compound **4**, compound **10**) in the alcohols and amines containing targeted molecules. We found that PtOEP:compound **3** and PtOEP:compound **4** were effectively photolyzed under green light illumination, respectively, in 72.3%, 51.0% yield. Under far red-light illumination, PdTPBP:compound **9** and PdTPBP:compound **10** were also efficiently photocleaved. The photolytic yields are 65.3%, 52.4%, respectively (Fig. 2). Moreover, NIR light-activated TTAP “LEGO”s including PtTNP:compound **9** and PtTNP:compound **10**, were both compatible with this method, affording 27.8%, 29.3% photolytic yield (Fig. 2).

In addition to the above-mentioned amino acids, we also explored the possibility of extending TTAP compatible targeted molecules to small molecule drugs in current clinical use. In particular, small molecule drugs such as anti-inflammatory and anti-tumor drugs, are known to have severe systematic off-target side effects^18–20^. To this end, light-activatable prodrugs constitute emerging major targets for utilization in drug discovery, due to their high spatiotemporal resolution in the treatment of complex diseases^21,22^. Here, we constructed a series of TTAP compatible prodrugs via the conjugation of a series of anti-nonsteroidal and anti-inflammatory drugs (naproxen, indomethacin, ibuprofen) with perylene via ester bonds (Fig. 2). These resultant compounds (compounds **16**–**18**) were denoted as pro-naproxen, pro-indomethacin, and pro-ibuprofen. In conjunction with the far-red light photosensitizer (PdTPBP) or the NIR light photosensitizer (PtTNP), we observed that pro-naproxen (compound **16**) has excellent photolytic reaction yields of 78.5% (PdTPBP) and 17.4% (PtTNP). For the pro-indomethacin (compound **17**), the photolytic reaction yield is 51.7% (PdTPBP) and 9.2% (PtTNP). In addition, the photolytic reaction yields are also found to be quite effective: 71.6% (PdTPBP), and 19.4% (PtTNP) for pro-ibuprofen (compound **18**). The carbonate photolabile linkage containing pro-cholesterol (compound **20**) was also constructed and can be selectively photolyzed in the yields of 69.4% (PdTPBP) and 28.6% (PtTNP) under 1 h far-red or NIR light illumination (Fig. 2).

### Designing aqueous soluble TTAP nanoparticles to illustrate biological applications of TTAP

Next, in order to illustrate the biological applications via TTAP, we chose chlorambucil, which is an FDA approved anti-cancer drug to evaluate the effect of cancer treatment^23^. In this regard, we conjugated Py with chlorambucil to obtain pro-chlorambucil (compound **19**). When PdTPBP or PdTNP were used as the coupling light partners, we observed the respective photolytic yield of 72.8% (PdTPBP) (Supplementary Table 14) and 30.4% (PtTNP) (Supplementary Table 15). Furthermore, we studied the relationship between the incident light intensity and the photolysis yield for the pair of PdTPBP and compound **19**. We compared the photolysis yield under the same photon flux (36 J cm^-2^) but with different power density and experimental duration. As a result, the photolysis yield was found to be 47% under the higher incident light power density and a short time duration (20 mW cm^−2^, 30 min) (Supplementary Table 14, entry 3). However, the photolysis yield of only 26% was observed under the lower incident light power density and the longer duration (10 mW cm^−2^, 60 min) (Supplementary Table 14, entry 4). These results suggested that the photolysis yield is light intensity dependent, thus further supporting our proposed model. We then designed an oleylamine-substituted amphiphilic polymer (PSMA-PEG-OAm) encapsulated TTAP nanoparticles to resolve the noxious oxygen quenching of the triplet states of Sen and PPGs-X under ambient condition (Supplementary Fig. 16). This unsaturated olefin-modified amphiphilic polymer was found to be able to encapsulate Sen and PPG-X to form air-stable monodispersed and water soluble ultra-small sized TTAP nanoparticles (TTAP NPs) (Supplementary Figs. 17–19).

Via ^1^H NMR spectra and the phosphorescence quenching experiment (Supplementary Fig. 16), we found that the unsaturated olefins can react with singlet oxygen to exhaust oxygen from the stored solution under light illumination. Considering the outstanding photolytic yield of the combination PdTPBP:compound **19**, we prepared TTAP NPs that contain PdTPBP and compound **19**. The entrapment and drug-loading efficiency are measured to be 89% and 16% in TTAP NPs. Via transmission electron microscope (TEM) and dynamic light scattering (DLS) characterization, the TTAP NPs have ultra-small size (12.9 ± 2.6, 29.7 ± 4.6 nm, respectively). Moreover, after 30 days, the size also maintained at ~30 nm via DLS, suggesting the stability of TTAP NPs (Supplementary Fig. 17). Via the UV–vis absorption of TTAP NPs, we further confirmed that TTAP NPs contain PdTPBP and compound **19** (Supplementary Fig. 18). In addition, after 650 nm (20 mW cm^−2^) light irradiation for 30 min, we did not observe significant photobleaching, suggesting that TTAP NPs have robust photostability (Supplementary Fig. 19). We then tested the photolytic kinetic process of TTAP NPs (Supplementary Figs. 20 and 21). After 60 min 20 mW cm^−2^ 650 nm LED illumination, 64% prodrug was effectively photolyzed. Moreover, the photolysis process was clearly dependent on the ON–OFF pattern of the LED excitation (Supplementary Fig. 21b). These results clearly validated the feasibility of the use of our TTAP “LEGO” system in aqueous solution under ambient air. The dose and duration of the prodrug activation can be precisely interrogated by our TTAP nanoparticles.

We then conducted in vitro and in vivo studies of our TTAP NPs. Firstly, via (3-(4,5-dimethylthiazol-2-yl)-2,5-diphenyltetrazolium bromide) tetrazolium reduction (MTT) assays, we demonstrated that TTAP NPs significantly enable the inhibition of cancer cell growth (Supplementary Fig. 22). This result suggests that via TTAP, the prodrug (pro-chlorambucil) was photolyzed and released from TTAP NPs into cancer cells, causing cancer cell death under far red-light illumination. Moreover, we evaluated the phototoxicity of PdTPBP NPs per se. However, we did not observe obvious cell death in the presence of the light, suggesting the negligible phototoxicity of PdTPBP (Supplementary Fig. 23).

**Fig. 3:**
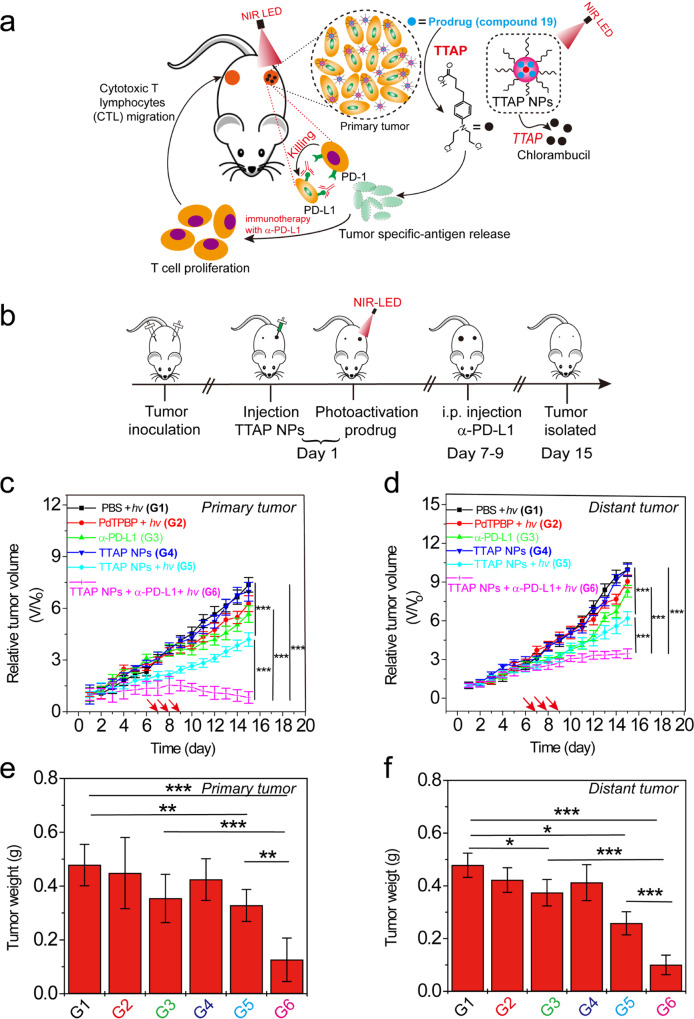
TTAP NPs-mediated synergistic immunotherapy. **a** A schematic illustration of the in vivo study of TTAP NPs-mediated prodrug photoactivation with checkpoint blockade the α-PD-L1 antibody to realize synergistic immunotherapy in a bilateral model of 4T1 tumor-bearing BALB/c mice. The right-side tumor stands for primary tumor meanwhile the left side tumor stands for distant tumor or “metastatic tumor”. **b** A schematic illustration of the experimental immunotherapy process. At first, the 4T_1_ cells were seeded at both right back and left back of mice. When the right-side tumor volume reached to 100 mm^3^, the TTAP NPs was injected into the right-side tumor, which was then illuminated by NIR LED (20 mW cm^−2^, 650 nm). This day was called as Day 1. At Day 7–9, the α-PD-L1 was intraperitoneally (i.p.) injected for mice of groups 3 and 6. Finally, the tumor of mice was isolated on Day 15. **c** The growth curves of primary tumors. **d** The growth curves of distant tumors. The red arrow stands for i.p. injection α-PD-L1 for groups 3 and 6 at 7th, 8th, and 9th day. **e** The primary tumor weight of mice. **f** The distant tumor weight of mice. “G” is the abbreviation of group, values are means ± s.e.m. (*n* = 5 mice per group). ^*^p < 0.05, ^**^p < 0.01, and ^***^p < 0.001.

Next, we went on to examine the synergistic anti-tumor immunotherapy effect of our TTAP system in conjunction with the checkpoint blockade PD-L1 anti-body (α-PD-L1) in a bilateral model of 4T_1_ tumor-bearing BALB/c mice^24,25^. As shown in Fig. 3a, the tumor on the right represented the primary tumor that was subjected to the injection of TTAP NPs and the subsequent light treatment. The tumor on the left was untreated and served to mimic a distant metastatic tumor. When the tumor on the right reached 100 mm^3^, TTAP NPs were intratumorally injected and this day was named Day 1. After 4 h, this tumor was exposed to NIR light (650 nm, 20 mW cm^−2^) for 30 min. At the 7th, 8th, and 9th day, the mice were i.p. injected with the α-PD-L1 (75 µg per mouse) (Fig. 3b). The therapeutic efficacy of different treatment groups was evaluated by measuring tumor volume and weight. The volume growth rates for the tumor on the right and left are presented, respectively, in Fig. 3c and d. In group 5 (TTAP NPs + *hv*), we only observed that the right tumor volume was more suppressed than that observed in groups 1–4. In contrast, for group 6 (TTAP NPs + *hv* + α-PD-L1), both the right and left tumors volume showed obvious reduction (Fig. 3 and Supplementary Fig. 24). Moreover, after 15 days, the right and left tumors were isolated and then weighed the tumor mass, respectively, as shown in Fig. 3e and f, which were consistent with the growth trend of tumor volume. These experimental results clearly demonstrated that the TTABP-mediated prodrug photolytic system potentiated the checkpoint blockade immunotherapy efficacy and promoted abscopal effects.

We next explored the mechanism by which TTAP-mediated prodrug photolysis enhanced the efficacy of immunotherapy (Supplementary Figs. 24–27). In particular, we first analyzed immune cell profiling in the spleen (Supplementary Figs. 25a, b and 26). The cytotoxic CD8^+^ T cell and helper CD4^+^ T cell levels significantly increased in the treated group, as compared to those in the PBS treatment group (group 1). After stimulation with PMA/ionomycin for 4 h, the cytokine of IFN-γ produced in CD4^+^ and CD8^+^ T cells were counted. The number of antigen-specific IFN-γ-producing T cells significantly increased in group 6, suggesting that TTAP plus α-PD-L1 treatment induced a tumor-specific T cell response. We further profiled infiltrating leukocytes in the primary and the distant tumors. Flow cytometry measurements for group 6 showed a significant increase in tumor-infiltration of CD4^+^ and CD8^+^ T cells in both primary and distant tumors (Supplementary Figs. 25c–f and 27). These results demonstrated TTAP-mediated prodrug plus α-PD-L1 treatment increased the infiltration of the effector T cells to treat the metastasis.

We also tested whether there was any in vivo toxicity from TTAP NPs by measuring the body weight of mice in each cohort (Supplementary Figs. 28–30 and Supplementary Table 16). The body weight experiment showed negligible side-effects (Supplementary Fig. 28). Furthermore, we compared the hematoxylin and eosin (H&E)-stained images of the major organs (heart, liver, spleen, lung, and kidney) from normal mice to those treated with TTAP NPs and light. Neither results displayed noticeable organ damage or inflammation lesions, suggesting that no obvious heart, liver, spleen, lung, or kidney dysfunction for the mice were induced by the photoactivatable process using TTAP NPs (Supplementary Fig. 29). Further, as shown in Supplementary Table 16, we did not observe abnormal results from the serum analysis experiments, which suggests that no observable unwanted inflammation was induced. Excrement from the mice 96 h after an IR806 dye-conjugated TTAP NPs intravenous (i.v.) injection was also collected. Compared to the PBS-injected control group, we detected the fluorescence of the IR806 dye signal in the excrement of mice in the TTAP NPs-treated group (Supplementary Fig. 30), suggesting that TTAP NPs can be cleared from the body through the fecal route. Besides, as shown in Supplementary Fig. 31, in addition to liver, the fluorescence was also observed at kidney at 10, 24, 48 h and disappeared at 96 and 168 h, suggesting the RGD-TTAP NPs may also be excluded from the body through kidney, which is similar to other nanoparticles^26,27^ (Supplementary Fig. 31). All of these results demonstrate that the as-designed TTAP NPs possess high biosafety and are highly biocompatible.

Finally, to achieve targeted tumor therapy, we covalently conjugated the cyclical Arg-Gly-Asp (cRGD) peptide on the surface of TTAP NPs (RGD-TTAP NPs) to enhance their tumor targeting properties (Supplementary Figs. 31 and 32). To verify the targeted tumor-killing effect of RGD-TTAP NPs in vivo, we utilized mice bearing a subcutaneous 4T_1_ tumor xenograft. To explore the best accumulation time-point of RGD-TTAP NPs in the tumor tissue, the 4T1 tumor-bearing mice were intravenously injected with IR806 fluorescence dye modified RGD-TTAP NPs (IR806-RGD-TTAP NPs) and subjected to in vivo imaging at different time-points. The fluorescence at the tumor site increased gradually and reached a maximum level 24 h post-injection. After 48 h, the fluorescence intensity of IR806-RGD-TTAP NPs in the 4T_1_ tumor gradually decreased. As an additional control, the cRGD free nanoparticles (IR806-TTAP NPs) were also intravenously injected to mice. However, the IR806-TTAP NPs-treated mice displayed a much weaker contrast between normal and tumor tissues (Supplementary Fig. 32b). This result demonstrates that the cRGD peptide actually improved the targeting to the tumor of TTAP NPs in our system.

As the accumulation of IR806-RGD-TTAP NPs in the tumor reached its maximum at 24 h, we then examined the treatment effect of IR806-RGD-TTAP NPs accompanied by irradiation with LED (650 nm, 20 mW cm^−2^) for 30 min. After such treatment, the therapeutic effects were assessed by monitoring changes in tumor volume (Supplementary Fig. 32d) as well as by H&E staining of the tumor tissues (Supplementary Fig. 32f). We only observed that the tumor growth in the treatment group 6 (IR806-RGD-TTAP NPs + *hv*) was remarkably suppressed. From the H&E-staining analysis, the tumor tissue also showed clear necrosis, which indicates that IR806-RGD-TTAP NPs can be effectively activated by far red LED irradiation for intense anticancer effect.

## Discussion

In sum, photolytic reaction has powerful and important applications in numerous aspects of chemistry, materials as well as biology. Our discovery of long wavelength single photon-driven TTAP overcomes the key problems (low effectiveness and high photodamage) in existing methods. This method is “LEGO”-like and highly modular. The light partners (the photosensitizers and the PPGs-X) can be energetically matched to adapt to the needs of a wide range of electromagnetic spectrum wavelengths and numerous chemical structures of PPGs, photolabile linkages, and a broad range of targeted molecules. Moreover, we exemplified the biological promise of TTAP via creating an ambient air-stable, ultra-small, water-soluble TTAP nanoparticle. Such nanoparticles achieve highly effective, ultra-low power, long wavelength single photon-driven anticancer prodrug activation and, in turn, potentiated anti-tumor immunotherapeutic responses and promoted abscopal effects.

## Methods

### Photolytic experiments

The NIR LED (720 nm), far red LED (650 nm), green LED (530 nm), blue LED (476 nm), and deep blue LED (455 nm) were used for photolytic reactions. The different pairs of photosensitizers (PtTNP, PdTPBP, PtOEP, or Ir(ppy)_3_) and PPGs-X (BDP, perylene, An, or Cou) were degassed for at least 15 min with argon in THF/H_2_O (4/1, v/v). Then, the solution was excited with a LED (20 mW cm^−2^) at 37 ^o^C. After the photolytic reaction, the raw product was dried and re-dissolved in CH_3_CN/H_2_O = (2/1, v/v) and HPLC was used to analyze the yield of product.

### The Stern–Volmer quenching plot experiment

The photoluminescence of photosensitizers (PtTNP, PdTPBP, PtOEP, and Ir(ppy)_3_) in the presence of different concentrations of PPGs including BDP (compound **7**), perylene (compound **8**), An (compound **2**), and Cou (compound **1**). The mixed solutions were degassed for at least 15 min with argon in toluene. The *k*_sv_ constants were calculated with the following equation^28^:

$$\frac{{I_{\rm{{O}}}}}{{I_{\rm{{t}}}}} = 1 + k_{\rm{{{sv}}}}Q,$$

where *I*_o_ and *I*_t_ stand for photoluminescence intensity of photosensitizer in the absence of PPGs-X and the photoluminescence intensity of photosensitizer in the presence PPGs-X. *Q* is the concentration of PPGs-X. Bimolecular quenching constants (*k*_q_) were calculated by the following equation^28^:

$$k_{\rm{{{sv}}}} = k_{\rm{{q}}} \times \tau _{\rm{{T}}}$$

The *τ*_T_ is the phosphorescence lifetime of photosensitizer in argon.

### The upconversion quantum yields (Φ_UC_) calculation^12^

The upconversion quantum yields were calculated with the following equation:

$${\Phi}_{{\mathrm{UC}}} = 2 \times {\Phi}_{{\mathrm{std}}} \times \frac{{{A}_{{\mathrm{std}}}}}{{{A}_{{\mathrm{unk}}}}} \times \frac{{{I}_{{\mathrm{unk}}}}}{{{I}_{{\mathrm{std}}}}} \times \left( {\frac{{{\eta }}_{{\mathrm{unk}}}}{{{\eta }}_{{\mathrm{std}}}}} \right)^2,$$

where Φ_UC_ and Φ_std_ stand for the upconversion luminescence quantum yield of the TTA-UC (TTAP 1–6) samples and the fluorescence quantum yield of the reference compounds, respectively. *A*_unk_ and *A*_std_ stand for absorbance of the TTA-UC samples and the reference compound, respectively. *I*_unk_ and *I*_std_ stand for the integrated upconversion luminescence intensity of the TTA-UC sample and the fluorescence intensity of the reference compounds, respectively. *η*_unk_ and *η*_std_ stand for the refractive index of solvents of the TTA-UC samples and the reference compounds. The solvent used is toluene (*η*_unk_ = 1.4967) for all of the TTAP pairs. The equation is multiplied by a factor of 2 to make the maximum quantum yield of two unified emitters. For the pairs: (1) PtTNP:BDP; (2) PtTNP:Py, the reference compound is ZnPc, the fluorescence quantum yield (Φ_*f*_ = 17%) in DMF (*η*_std_ = 1.333). For the pairs of (3) PdTPBP:BDP, (4) PdTPBP:Py, the reference compound is methylene blue (MB), and its fluorescence quantum yield (Φ_*f*_ = 3%) was measured in methanol (*η*_std_ = 1.333). For the pairs of (5) PtOEP:Py and (6) PtOEP:An, the reference compound is rhodamine B, and the fluorescence quantum yield (Φ_*f*_ = 49%) was measured in ethanol (*η*_std_ = 1.361).

The upconversion experiments was conducted in deaerated solution with argon for ca. 15 min before each measurement and the argon gas flow was maintained during the measurement. Please see the respective quantum yield, as follows:

Φ_UC_ (PtTNP/Py) = 0.05%; Φ_UC_ (PtTNP/BDP) = 0.3%; Φ_UC_ (PdTPBP/Py) = 5.8%; Φ_UC_ (PdTPBP/BDP) = 8.4%; Φ_UC_ (PtOEP/Py) = 9.5%; Φ_UC_ (PtOEP/An) = 8.7%.

### Photolytic QE for TTAP systems^29^

The QE gives a comprehensive evaluation of photolysis reactions as it considers both photolysis quantum yield of PPGs and absorbance of sensitizers. We also have considered the TTET and TTA quantum yields in the calculation.

We can estimate the efficiency of Φ_TTET_ × Φ_TTA_ by the following formula^12^:

$$\Phi_{\mathrm{UC}}=\Phi_{\mathrm{ISC}} \times \Phi_{\mathrm{TTET}} \times \Phi_{\mathrm{TTA}} \times \Phi_{\mathrm{f}}.$$

In this formula, the Φ_ISC_ is the ISC quantum yield of the photosensitizer. Φ_TTET_ is TTET quantum yield between photosensitizer and annihilator. Φ_TTA_ is quantum yield of TTA for annihilator. Because TTET and TTA processes are a multi-molecular intermolecular energy transfer process, so far, there is still a lack of accurate determination of Φ_TTET_ and Φ_TTA_. However, we can calculate the result of Φ_TTET_ × Φ_TTA_ according to this above formula. The Φ_ISC_ for Ir(ppy)_3_^14^, PtOEP^30^, PdTPBP, and PtTNP^31^ is close to 1, and Φ_UC_ and Φ_f_ has been calculated according to reported protocol.

Φ_TTET_ × Φ_TTA_ (PtTNP/BDP) = 0.4%,

Φ_TTET_ × Φ_TTA_ (PtTNP/Py) = 0.06%,

Φ_TTET_ × Φ_TTA_ (PdTPBP/BDP) = 11.3%,

Φ_TTET_ × Φ_TTA_ (PdTPBP/Py) = 6.6%,

Φ_TTET_ × Φ_TTA_ (PtOEP/Py) = 10.9%,

Φ_TTET_ × Φ_TTA_ (PtOEP/An) = 20.1%.

At last, we considered the TTET and TTA processes to correct photolytic QE with the following equation:

$${\mathrm{{QE}}}=\varepsilon \times \Phi_{\mathrm{p}} \times \Phi_{\mathrm{TTET}} \times \Phi_{\mathrm{TTA}}.$$

QE is the photolytic QE, *ε* is molar extinction coefficient of sensitizers, and Φ_p_ is photolytic quantum yield of PPGs.

QE (PtTNP/BDP) = 0.53; QE (PtTNP/Py) = 7.1; QE (PdTPBP/BDP) = 12.3; QE (PdTPBP/Py) = 679; QE (PtOEP/Py) = 708; QE (PtOEP/An) = 1255.

In the UCNPs-assist photolysis, the upconversion quantum yield is low (<0.1% UV upconversion). Moreover, the LRET efficiency is not high (<10%). Therefore, these systems not only consider the photolytic quantum yield (Φ_p_) of PPGs, but also the upconversion quantum yield (Φ_uc_) and LRET (Φ_LRET_) to get the true photolytic QE with the following equation:

$${\mathrm{{QE}}} \, {\mathrm{(upconversion)}}=\Phi_{\mathrm{p}} \times \Phi_{\mathrm{uc}} \times \Phi_{\mathrm{LRET}} \times\varepsilon_{\mathrm{(PPG)}} .$$

According to the reported results in the literature^32^, we calculated photolytic QE of Cou (QE_(Cou)_) to be 0.02873 (note: other PPGs including Py, BDP, and An have not been reported in the literature in combination with UCNPs-assist photolysis).

### Preparation of TTAP NPs

TTAP NPs was prepared via self-assembly of PdTPBP and compound **19**, and PSMA-PEG-OAm with a single-step. Briefly, 0.25 mg PdTPBP, 20 mg compound **19**, and 100 mg PSMA-PEG-OAm were dissolved in 8 mL THF. The mixture was added to 10 mL PBS buffer. The mixed solution was stirred at 40 °C in dark for 30 min to let THF remove out. In order to absolutely remove out the THF, we used the air flow to accelerates THF evaporation at 40 °C for 30 min. Afterward, the TTAP NPs was centrifuged at 6000 × *g* for 60 min. large nanoparticles precipitated and the supernatant containing small nanoparticles was decanted. And then the TTAP NPs solution was purified by dialysis tube (cut off *M*_w_ = 3500). Finally, TTA NPs was stored at 4 °C until use. Similar procedures were used to prepare the PdTPBP nanoparticles (PdTPBP NPs) and Py nanoparticles (Py NPs).

### The entrapment efficiency of dyes in nanoparticles

Firstly, before wrapping the dyes, we measured the absorbance of dyes in dichloromethane and determined the initial absorbance of dyes that was fed initially (*A*_0_). After encapsulating the dyes with PSMA-PEG-OAm, the nanoparticles were centrifugated (5000 × *g*) for 20 min to attain TTAP NPs. We then added the dichloromethane to extract the dyes from the nanoparticles, and to test the absorbance (*A*) to determine the amount of dyes in TTAP NPs. Then the entrapment efficiency of the dyes was calculated according to the following equation^33^.

Entrapment efficiency (%) = absorbance of dyes in nanoparticles (*A*)/absorbance of dyes fed initially (*A*_0_) × 100.

The PdTPBP entrapment efficiency is 89.0%.

According the entrapment efficiency of prodrug, the mass of prodrug was calculated in TTAP NPs. The TTAP NPs were also dried by lyophilization and weight the mass of TTAP NPs.

The prodrug loading efficiency (%) = the mass of prodrug in TTAP NPs /total mass of TTAP NPs × 100%.

The prodrug loading efficiency of prodrug is calculated to be 16%.

### In vitro experiments for TTAP NPs

Human cervical carcinoma (HeLa cell lines) and mice breast cancer cells (4T_1_ cell lines) were firstly cultured in Dulbecco’s modified Eagle’s medium (DMEM) containing 10% fetal bovine serum (FBS), 100 µg mL^–1^ streptomycin and 100 U mL^–1^ penicillin at 37 °C in a humidified incubator containing 5% CO_2_ and 95% air. The medium was replenished every other day and the cells were subcultured after reaching confluence. then, the cells (100 μL, 5000) were plated in a 96-well plate. After 12 h, the nanoparticles were added at different concentrations (0, 5, 10, 15, 20, 25 μg mL^–1^). The nanoparticles were taken up over 12 h. The cells were irradiated with far red LED light (650 nm, 20 mW cm^–2^). After 30 min irradiation, the cells were incubated another 24 h under 5% CO_2_ atmosphere at 37 °C. MTT solution (5.0 mg mL^–1^, 50 μL) was added to every well and left for 4 h. The old cell culture medium was removed carefully and 200 μL DMSO was added to every well. A microplate reader (Bio-Rad) was used to record the absorption at 595 nm. And the cell viability was calculated with the following equation:

$${\mathrm{Cell}} \, {\mathrm{viability}} (\%)= {\mathrm{OD}} \, {\mathrm{value}} \, {\mathrm{test}} / {\mathrm{OD}} \, {\mathrm{value}} \, {\mathrm{control}} \times 100\%.$$

### In vivo studies of combination of TTAP NPs with check-point blockade immunotherapy in 4T_1_ tumor-bearing mice

(The animal experiments followed the protocol approved by the Institutional Animal Care and Use Committee (IACUC), University of Masssachusttes Medical school) The 5 week’s BALB/c female mice were ordered from Jackson lab. The 4T_1_ cells (2 × 10^6^) was seeded at mice right back and the left mice back seeded the 4T_1_ cells (2 × 10^5^). After the right-side tumor volume reached to 100 mm^3^, the 4T_1_ bearing mice were subjected to six different treatments: (1) PBS + *hv*; (2) PdTPBP + *hv*; (3) α-PD-L1 only; (4) TTAP NPs + dark; (5) TTAP NPs + *hv*; (6) TTAP + *hv* +α-PD-L1. The TTAP NPs (1 mg mL^–1^, 100 μL) or PdTPBP NPs (10 μg mL^–1^, 100 μL) was intratumorally injected in right side tumor in groups 4–6 or group 2, respectively. After 4 h, the right tumor site was exposed to far red LED (650 nm) for 30 min. The α-PD-L1 (75 μg per mouse) was injected by intraperitoneal during 7−9 day in group 3 and 6. After 15 days treatment, the mice were euthanized and spleen, right and left tumor tissues were isolated to analyse the immunity responses. Different treatment groups were monitored by measuring the right and left tumor sizes using a Vernier caliper for 15 days. Tumor size = width × width × length/2.

### Flow cytometry test of the immune cells in tumors

The tumors (right and left side) were isolated from the mice after different treatments. The tumor tissue was divided into small pieces, treated with 1 mg mL^–1^ collagenase I (Gibco) for 1 h at 37 °C and ground using the rubber end of a syringe (BD, 10 mL syringe). Cells were filtered through nylon mesh filters (Corning, cell strainer, 70 μm nylon). The single cells were collected by centrifugation (800 × *g*, 5 min). The blood cells in the tumor tissue was eliminated by cold NH_4_Cl lysis. The single suspensions tumor cells were washed by cold PBS containing 2% FBS. The tumor cells were stained with fluorescence-labeled antibodies PerCP-Cy™5.5 Hamster Anti-Mouse TCR β chain (BD bioscience, clone H57-597, catalog No. 560657), PerCP-Cy™5.5 Rat Anti-Mouse CD3 Molecular Complex Clone (BD bioscience, catalog No. 560527), Pacific Blue™ Rat Anti-Mouse CD45R (clone RA3-6B2, BD bioscience, catalog No. 558108), PE-Cy™7 Rat Anti-Mouse CD4 (Clone GK1.5, BD bioscience, catalog No. 563933), PE Rat Anti-Mouse CD8a (Clone 53-6.7, BD bioscience, catalog No. 553032), APC Mouse Anti-Mouse NK-1.1 (Clone PK136, BD biosciences, catalog No. 561117), FITC Rat Anti-Mouse CD44 (Clone IM7, BD biosciences, catalog No. 561859), APC-Cy™7 Rat Anti-Mouse CD62L (Clone MEL-14, BD biosciences, catalog No. 560514) following the manufacturer’s instructions. All antibodies were diluted 200 times. Flow cytometric analyses were performed on an LSRFortessa (BD Biosciences) and analyzed using FlowJo Software (Tree Star). The results are showed as the mean ± standard error of the mean (s.e.m.) Moreover, Student’s *t*-test was used for two-group comparisons. The Origin 9.0 was used for all statistical analyses. The threshold for statistical significance was ***p < 0.001, **p < 0.01, or *p < 0.05.

### Flow cytometry test of the immune cells in the spleen

The spleens were isolated from the mice after different treatments. We grinded the spleen and cells, which were then filtered by nylon mesh filters. The blood cells were lysis by NH_4_Cl solution twice, and then washed with cold PBS containing 2% FBS. The single cells were collected by centrifugation (800 × *g*, 5 min), and the blood cells in the tumor tissue was eliminated by cold NH_4_Cl lysis. The cells were incubated with 50 ng mL^−1^ PMA (Sigma) and 500 ng mL^−1^ ionomycin (Sigma) in the presence of GolgiStop (BD) in complete T cell media at 37 °C for 4 h. Cells were washed with PBS buffer containing 1% FBS, stained with Live/Dead fixable aqua dead cell marker (Invitrogen). The following antibodies were used for staining: PerCP-Cy™5.5 Hamster Anti-Mouse TCR β chain (BD bioscience, clone H57-597, catalog No. 560657), PerCP-Cy™5.5 Rat Anti-Mouse CD3 Molecular Complex Clone (BD bioscience, catalog No. 560527), Pacific Blue™ Rat Anti-Mouse CD45R (clone RA3-6B2, BD bioscience, catalog No. 558108), PE-Cy™7 Rat Anti-Mouse CD4 (Clone GK1.5, BD bioscience, catalog No. 563933), PE Rat Anti-Mouse CD8a (Clone 53-6.7, BD bioscience, catalog No. 553032), APC Mouse Anti-Mouse NK-1.1 (Clone PK136, BD biosciences, catalog No. 561117). Intracellular staining for FITC Rat Anti-Mouse IFN-γ (Clone XMG1.2, BD biosciences, catalog No. 562019). Intracellular cytokine staining was performed by fixing cells in 2% paraformaldehyde, followed by permeabilization and staining (BD Biosciences). Flow cytometric analyses were performed on an LSRFortessa (BD Biosciences) and analyzed using FlowJo Software (Tree Star). The results are shown as the mean ± standard error of the mean (s.e.m.) Moreover, Student’s *t*-test was used for two-group comparisons. The Origin 9.0 was used for all statistical analyses. The threshold for statistical significance was ***p < 0.001, **p < 0.01, or *p < 0.05.

### Preparation of IR806-TTAP NPs

5 mL TTAP NPs in PBS buffer, 10 mg sodium sulfonate *N*-hydroxysuccinimide (NHS.SO_3_Na), and 10 mg *N*-(3-dimethylaminopropyl)-*N*′-ethylcarbodiimide hydrochloride (EDC.HCl) were added to the nanoparticle solution and the mixture was then stirred at room temperature for 5 h. After that the amine-substituted IR806 (0.5 mg) was added into the mixture, and stirred for another 24 h. Finally, the nanoparticles were purified by dialysis (*M*_w_ = 12,000).

### Body clearance of TTAP NPs

IR806-TTAP NPs were studied in 4T_1_ tumor-bearing mice. They were subjected to the following treatments: group 1, intravenous injection of IR806-TTAP NPs (150 μL, 100 μg mL^–1^); group 2, intravenous injection of PBS. We collect the mice feces during 48 h, and then measured the fluorescence of IR806 with IVS animal imaging. Excitation filter was ICG (750–790 nm) and emission filter was ICG (815–850 nm).

### Preparation of IR806-RGD-TTAP NPs

5 mL IR806-TTAP NPs in PBS buffer, 10 mg sodium sulfonate *N*-hydroxysuccinimide (NHS-SO_3_Na) and 10 mg *N*-(3-dimethylaminopropyl)-*N*′-ethylcarbodiimide hydrochloride (EDC·HCl) were added to the nanoparticle solution and the mixture was then stirred at room temperature for 5 h. After that the *c*RGD (3 mg) was added into the mixture, and stirred for another 24 h. Finally, the nanoparticles were purified by dialysis (*M*_w_ = 120,000).

### Tumor-targeted property on subcutaneous 4T1 bearing breast tumor model

Tumor-targeting property of IR806-TTAP NPs and IR806-RGD-TTAP NPs was studied in 4T_1_ tumor-bearing mice. They were subjected to the following treatments: Group 1: intravenous injection of IR806-RGD-TTAP NPs; Group 2: intravenous injection of IR806-TTAP NPs. The dose of NPs is 150 μL, 100 μg mL^–1^ in saline. We measured the fluorescence of IR806-TTAP NPs and IR806-RGD-TTAP NPs at different times (10, 24, 48, 96, 168 h) with IVS animal imaging. Excitation filter was ICG (750–790 nm) and emission filter was ICG (815–850 nm).

### In vivo bio-distribution of IR806-RGD-TTAP NPs

After tail-vein injection of IR806-RGD-TTAP NPs, the 4T_1_ tumor-bearing mice were sacrificed, and major organs including heart, liver, spleen, lung, and kidney were carefully removed for visualization under the IVS animal imaging system in different time periods (10, 24, 48, 96, and 168 h). The fluorescent signals of each organ were analyzed by the accompanied software.

### In vivo studies of RGD-TTAP NPs for tumor inhibition in 4T_1_ tumor-bearing mice

The mice were subjected to six different treatments: PBS control group (group 1), treatment with light irradiation only (group 2), RGD-TTAP NPs-intravenous injection only (group 3), TTAP NPs-intravenous injection and then irradiation (group 4), PdTPBP NPs-intravenous injection and then irradiation (group 5), and RGD-TTAP NPs-intravenous injection and then treatment with irradiation (group 6). After 24 h, far-red light LED (650 nm) analysis was performed on groups 2, 4, 5, and 6 at 20 mW cm^–2^ for 30 min. Two mice from each group were euthanized 14 days post-treatment, and tumor tissues of the above-mentioned treatment groups 1−6 were harvested for histological study by H&E staining under a BX51 optical microscope (Olympus, Japan) in a blinded fashion by a pathologist. Different treatment groups were monitored by measuring the tumor size using a Vernier caliper for 14 days. Tumor size = width × width × length/2.

## Supplementary information

Supplementary information

## Data Availability

The experimental procedures, data, and analysis supporting the conclusions of this work can be found in the figures and Supplementary Information. Additional data are available from the corresponding author upon request.
