# Supplementary material for: Long wavelength single photon like driven photolysis via triplet triplet annihilation
Source: Nat Commun. 2021 Jan 5;12:122. doi: 10.1038/s41467-020-20326-6 (PMC7785739; doi:10.1038/s41467-020-20326-6)
Supplement: Supplementary file 1 — Supplementary information [file 41467_2020_20326_MOESM1_ESM.pdf]

Supplementary Information

Long Wavelength Single Photon Like Driven Photolysis Via Triplet-Triplet Annihilation

*Ling Huang, Le Zeng, Yongzhi Chen, Nuo Yu, Lei Wang, Kai Huang, Yang Zhao, Gang Han**

Department of Biochemistry and Molecular Pharmacology, University of Massachusetts Medical School, USA.

Supplementary Methods

Chemicals: perylene, tris [2-phenylpyridinato-C_2, N] iridium (III) ($Ir(ppy)_3$) were purchased from TCI chemicals. The solvents of dimethylformamide (DMF), phosphoryl chloride ($POCl_3$), sodium borohydride ($NaBH_4$), methanol, acetyl chloride, pyridine, dicyclohexylcarbodiimide (DCC), 4-(dimethylamino) pyridine (DMAP); triethylamine (TEA) *N*-butylamine, dry CH_2Cl_2, 3-(4,5-dimethylthiazol-2-yl)-2,5-diphenyltetrazolium bromide (MTT), propidium iodide (PI), 9-anthracenemethanol, 4-nitrophenylchloroformate, chloro (3-methylbutoxy) methanone, *N*-pivaloyl glycine, 4-[(2,2-dimethylpropanoyl)amino] butanoic acid and poly(styrene-co-maleic anhydride) (PSMA, M_w = 1600) were purchased from Sigma-Aldrich (St. Louis, MO, USA). meso-tetraphenyltetrabenzoporphine palladium complex (PdTPBP) and platinum octaethylporphyrin (PtOEP) were purchased from Frontier Scientific, Inc. Dulbecco's Modified Eagle Medium (DMEM), fetal bovine serum (FBS), penicillin-streptomycin, trypsin-EDTA (EDTA = ethylenediaminetetraacetic acid), and phosphate-buffered saline solution (PBS) were obtained from Invitrogen (Carlsbad, CA, USA). Ultrapure water was prepared by using a Millipore Simplicity System (Millipore, Bedford, USA). All of the above-mentioned chemicals were used as received without further purification.

Characterization: ^{1}H NMR spectra were recorded with a Bruker 500 MHz spectrometer. UV−Vis spectra were recorded on an Agilent Cary-5 spectrophotometer. Steady-state fluorescence spectra were measured on a HITACHI F-7000 spectrometer. The morphology of the TTAP NPs was characterized by using a JEOL JEM-200CX transmission electron microscope (TEM) operated at 80 kV. The sample for TEM measurement was prepared by dropping the solution onto a carbon-coated copper grid following negative staining with 2.0% (w/v) phosphotungstic acid. The particle size and size distribution were measured by using dynamic light scattering (DLS) on a Malvern Nano-ZS particle size analyzer, excitation wavelength 532 nm. All samples in the Stern-Volmer quenching plots experiments were deaerated with argon for ca. 15 min before measurement and the gas flow was maintained during the measurements. For the TTA upconversion spectra measurement, the diode pumped solid-state laser (650 nm, continues wave, CW, Hi-Teach company, China) was used as the excitation light source and a modified spectrofluorometer was used to record the upconversion spectra. For the prodrug photolytic experiment, all the LEDs

(Mightex Company) were used as the excitation light source.

Preparation and characterization of PPGs-X.

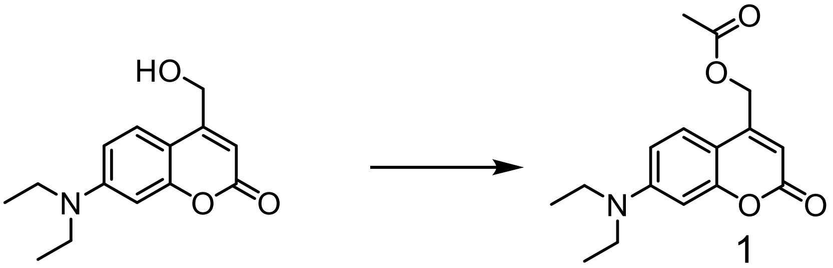

Compound 1: 4-benzyl alcohol coumarin was synthesized according to the previous synthesis protocols.[1]

Then 4-benzyl alcohol coumarin (247.1 mg, 1.0 mmol) was dissolved in dry dichloromethane (DCM) (10 mL) and pyridine (50 μL) were mixed. Acetyl chloride (2.0 mmol) was dissolved in dry DCM (10 mL) and added dropwise to the mixture over 10 min and then stirred for 2 hours. After the reaction, the solvents were removed under vacuum condition. The raw **1** was purified by silica gel flash column chromatography with DCM/n-hexane =1/1 (v/v) as elute. The yellow band was collected and yield determined to be 92% (266.9 mg). ^{1}H-NMR (500 MHz, $CDCl_3$): δ = 7.29-7.28 (d, 1H, J = 5.0 Hz,), 6.59-6.57 (d, 1H, J =10.0Hz), 6.52 (s, 1H), 6.14-6.13 (d, 1H, J = 5.0 Hz), 5.22 (s, 2H), 3.44-3.39 (m, 4H), 2.19 (s, 3H), 1.22-1.20 ppm (m, 6H). ^{13}C-NMR (125 MHz, $CDCl_3$): δ = 150.7, 149.4, 124.4, 108.7, 106.5, 106.0, 97.9, 61.4, 44.8, 20.8, 12.4 ppm. HRMS (ESI) exact mass calculated for $[M + H]^+$ ($C_{16}H_{19}NO_4 + H^+$) m/z 290.1392, found m/z 290.1387.

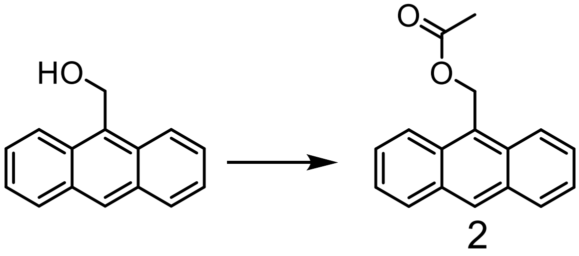

Compound 2: 4-benzyl alcohol anthracene (208 mg, 1.0 mmol) dissolved in dry dichloromethane (DCM) (10 mL) and pyridine (50 μL) were mixed. Acetyl chloride (2.0 mmol) was dissolved in

dry DCM (10 mL) and added dropwise to the mixture over 10 min and then stirred for 2 hours. After the reaction, the solvents were removed under vacuum condition. The raw **2** was purified by silica gel flash column chromatography with DCM/n-hexane =1/2 (v/v) as elute. The yellow band was collected and yield determined to be 85% (212.5 mg). ^{1}H-NMR (500 MHz, $CDCl_3$): δ = 8.52 (s, 1H), 8.34-8.32(d, 2H, J = 10.0Hz), 8.05-8.03 (d, 2H, J = 9.5 Hz), 7.58-7.56 (m, 2H), 7.50-7.48 (m, 2H), 6.16 (s, 2H), 2.09 ppm (s, 3H). ^{13}C-NMR (125 MHz, $CDCl_3$): δ = 171.3, 131.4, 131.1, 129.2,129.1, 126.7, 126.2, 125.1, 123.9, 58.8, 21.0 ppm. HRMS (ESI) exact mass calculated for [M + Na^+] ($C_{17}H_{14}O_2$ + Na^+) m/z 273.0891, found m/z 273.0886.

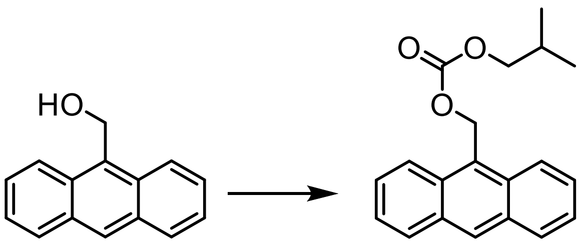

3

Compound 3: 4-benzyl alcohol anthracene (208 mg, 1.0 mmol) dissolved in dry dichloromethane (DCM) (10 mL) and pyridine (50 μL) were mixed. Isobutyl chloroformate (2.0 mmol) was dissolved in dry DCM (10 mL) and added dropwise to the mixture over 10 min and then stirred for 2 hours. After the reaction, the solvents were removed under vacuum condition. The raw **3** was purified by silica gel flash column chromatography with DCM/n-hexane =1/2 (v/v) as elute. The yellow band was collected and yield determined to be 65% (200.2 mg). ^{1}H-NMR (500 MHz, $CDCl_3$): δ = 8.51 (s, 1H), 8.40-8.38 (d, 2H, J = 10.0Hz), 8.03-8.01 (d, 2H, J = 9.5Hz), 7.60-7.56 (m, 2H), 7.50-7.47(m, 2H), 6.22(s, 2H), 3.96-3.94 (d, 2H, J = 10.0Hz), 1.98-1.91 (m, 1H), 0.92 (s, 3H), 0.90 ppm (s, 3H). ^{13}C-NMR (125 MHz, $CDCl_3$): δ = 155.68, 131.4, 131.2, 129.5, 129.1, 126.8, 125.1, 124.0, 123.9, 74.4, 74.0, 62.1, 27.8, 18.9 ppm. HRMS (ESI) exact mass calculated for [M + Na]$^+$ ($C_{20}H_{20}O_3$ + Na^+) m/z 331.1310, found m/z 331.1305.

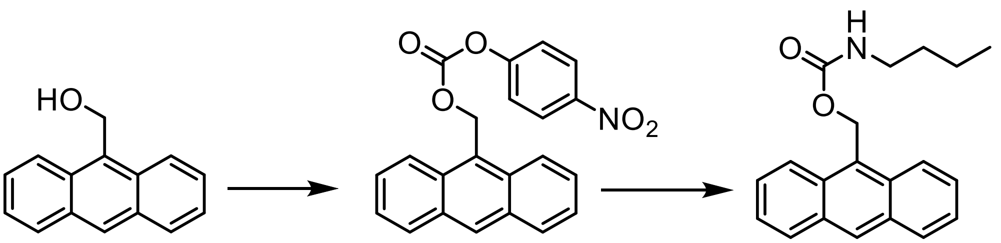

4

Compound 4: 4-benzyl alcohol anthracene (208 mg, 1.0 mmol) dissolved in dry dichloromethane (DCM) (10 mL) and pyridine (50 μL) were mixed. 4-nitrophenyl chloroformate (1.2 mmol) was dissolved in dry DCM (10 mL) and added dropwise to the mixture over 10 min and then stirred for 2 hours. Next, the butylamine (2.0 mmol) was added and stirred for another 12 hours in room temperature. After the reaction, 50 mL CH_2Cl_2 was added. The solutions were washed with saturated aqueous solution of sodium bicarbonate (30 mL). The organic layer was collected and dried by Na_2SO_4; the solvents were removed under vacuum condition. The raw **4** was purified by silica gel flash column chromatography with DCM/n-hexane =1/1 (v/v) as elute. The shallow yellow band was collected and yield determined to be 60% (184.2 mg). ^{1}H-NMR (500 MHz, $CDCl_3$): δ= 8.50 (s, 1H), 8.40-8.38 (m, 2H), 8.03-8.01 (d, 2H, J =9.0 Hz), 7.57-7.55 (m, 2H), 7.50-7.47 (m, 2H), 6.14 (s, 2H), 4.66 (s, 1H), 3.23-3.20 (m, 2H), 1.50-1.44 (m, 2H), 1.35-1.30 (m, 2H), 0.92-0.90 ppm (m, 3H). ^{13}C-NMR (125 MHz, $CDCl_3$): δ= 156.6, 131.4, 131.0, 129.1, 129.0, 126.9, 126.6, 125.1, 124.1, 59.0, 40.9, 32.0, 19.9, 13.7 ppm. HRMS (ESI) exact mass calculated for [M + Na]$^+$ ($C_{20}H_{21}NO_2$ + Na^+) m/z 330.1470, found m/z 330.1465.

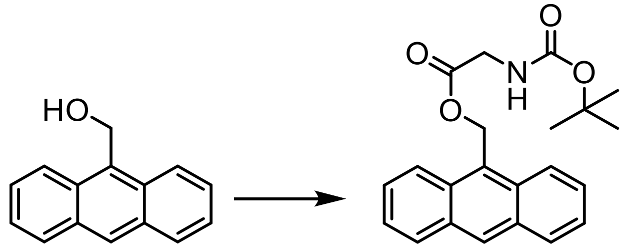

5

Compound 5: 4-benzyl alcohol anthracene (208 mg, 1.0 mmol) and Boc-Gly-OH (1.5 mmol) dissolved in dry dichloromethane (DCM) (10 mL) in 0 °C. Next, the 4-dimethylaminopyridine (DMAP, 0.2 mmol) and *N*, *N*'-dicyclohexylcarbodiimide (DCC, 1.2 mmol) were added to the mixture and then stirred for 12 hours. After the reaction, the solvents were removed under vacuum condition. The raw **5** was purified by silica gel flash column chromatography with DCM/n-hexane =1/1 (v/v) as elute. The shallow yellow band was collected and yield determined to be 71% (260 mg). ^{1}H-NMR (500 MHz, $CDCl_3$): δ= 8.53 (s, 1H), 8.33-8.32 (d, 2H, J = 5.0 Hz), 8.04-8.03 (d, 2H, J = 5.0Hz), 7.60-7.57 (m, 2H), 7.52-7.49 (m, 2H), 6.24 (s, 2H), 4.98 (s, 1H), 3.93 (s, 2H), 1.42 ppm (s, 9H). ^{13}C-NMR (125 MHz, $CDCl_3$): δ = 131.4, 131.1, 129.5, 129.2, 126.9, 125.2, 123.8, 59.7, 42.5, 28.3 ppm. HRMS (ESI) exact mass calculated for [M + Na]$^+$ ($C_{22}H_{23}NO_4$ + Na^+) m/z

388.1525, found m/z 388.1519.

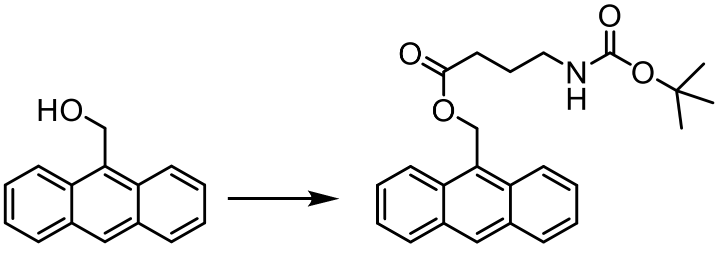

6

Compound 6: 4-benzyl alcohol anthracene (208 mg, 1.0 mmol) and Boc-GABA-OH (1.5 mmol) dissolved in dry dichloromethane (DCM) (10 mL) in 0 °C. Next, 4-dimethylaminopyridine (DMAP, 0.2 mmol) and *N*, *N*'-dicyclohexylcarbodiimide (DCC, 1.2 mmol) were added to the mixture and then stirred for 12 hours. After the reaction, the solvents were removed under vacuum condition. The raw **6** was purified by silica gel flash column chromatography with DCM/n-hexane =1/1 (v/v) as elute. The shallow yellow band was collected and yield determined to be 80% (314 mg). ^{1}H-NMR (500 MHz, $CDCl_3$): δ = 8.51 (s, 1H), 8.34-8.32 (d, 2H, J = 10.0Hz,), 8.04-8.03 (d, 2H, J = 5.0 Hz,), 7.59-7.56 (m, 2H), 7.51-7.48 (m, 2H), 6.17 (s, 2H), 4.54 (s, 1H), 3.13-3.12 (d, 2H, J = 5.0 Hz), 2.39-2.36 (m, 2H), 1.82-1.78 (m, 2H), 1.41 ppm (s, 9H). ^{13}C-NMR (125 MHz, $CDCl_3$): δ = 173.4, 155.9, 131.4, 131.0, 129.2, 129.1, 126.7, 126.2, 125.1, 123.9, 58.9, 39.8, 31.1, 28.4, 25.3 ppm. HRMS (ESI) exact mass calculated for $[M + Na]^+$ ($C_{24}H_{27}NO_4 + Na^+$) m/z 416.1838, found m/z 416.1832.

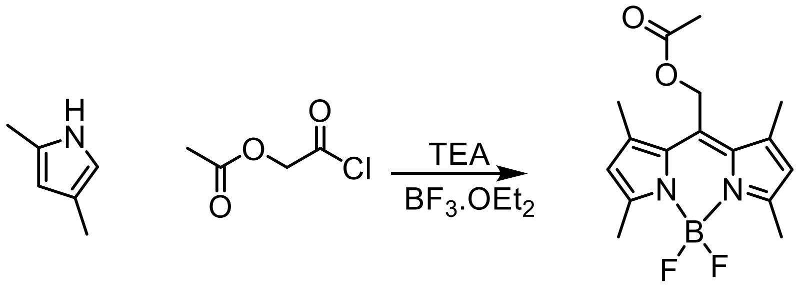

7

Compound 7: 2,4-dimethylpyrrole (980 mg, 10 mmol) and acetoxyacetyl chloride (5.0 mmol) dissolved in dry dichloromethane (DCM) (300 mL) at 0 °C and then stirred for 12 hours. After the reaction, 5 mL $BF_3.OEt_2$ was added dropwise for 20 min at 0 °C and stirred another 4 hours, the

solvents were removed under vacuum condition. The raw **7** was purified by silica gel flash column chromatography with DCM/n-hexane =1/1 (v/v) as elute. The shallow yellow band was collected and yield determined to be 15% (480 mg). ^{1}H-NMR (500 MHz, $CDCl_3$): δ= 6.09 (s, 2H), 2.53 (s, 6H), 2.36 (s, 6H), 2.13 (s, 3H), 1.54 ppm (s, 2H). ^{13}C-NMR (125 MHz, $CDCl_3$): δ= 170.6, 156.6, 141.5, 133.3, 132.7, 122.3, 57.9, 20.6, 13.6, 14.7 ppm. HRMS (ESI) exact mass calculated for $[M + H]^+$ ($C_{16}H_{19}BF_2N_2O_2 + H^+$) m/z 321.1586, found m/z 321.1580.

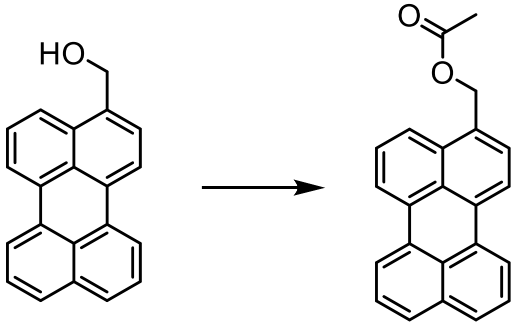

8

Compound 8: 1-benzyl alcohol perylene was first synthesized according to the previous synthesis protocols.[2] 4-benzyl alcohol perylene (140 mg, 0.5 mmol) dissolved in dry dichloromethane (DCM) (30 mL) and pyridine (50 μL) were mixed. Acetyl chloride (1.0 mmol) was dissolved in dry DCM (10 mL) and added dropwise to the mixture over 10 min and then stirred for 2 hours. After the reaction, the solvents were removed under vacuum condition. The raw **8** was purified by silica gel flash column chromatography with DCM/n-hexane =1/2 (v/v) as elute. The yellow band was collected and yield determined to be 95% (150 mg). ^{1}H-NMR (500 MHz, $CDCl_3$): δ= 8.26-8.24 (d, 1H, J = 10.0Hz), 8.22-8.20 (m, 3H), 8.17-8.16 (d, 1H, J = 5.0Hz), 7.72-7.70 (m, 2H), 7.58-7.54 (m, 2H), 7.51-7.48 (m, 2H), 5.51 (s, 2H), 2.14 ppm (s, 3H). ^{13}C-NMR (125 MHz, $CDCl_3$): δ = 171.1, 134.6, 133.0, 132.2, 131.9, 131.1, 130.9, 130.8, 129.0, 128.5, 128.3, 128.2, 128.1, 127.1, 126.6, 126.5, 123.3, 120.6, 120.5, 120.4, 119.6, 64.7, 21.1 ppm. HRMS (ESI) exact mass calculated for $[M + Na]^+$ ($C_{23}H_{16}O_2$+Na^+) m/z 347.1043, found m/z 347.1042.

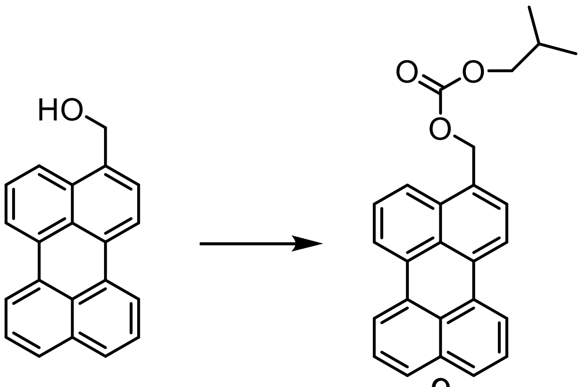

9

Compound 9: 4-benzyl alcohol perylene (140 mg, 0.5 mmol) was dissolved in dry dichloromethane (DCM) (30 mL) and pyridine (50 μL) were mixed. Isobutyl chloroformate (1.0 mmol) was dissolved in dry DCM (10 mL) and added dropwise to the mixture over 10 min and then stirred for 2 hours. After the reaction, the solvents were removed under vacuum condition. The raw **9** was purified by silica gel flash column chromatography with DCM/n-hexane =1/2 (v/v) as elute. The yellow band was collected and yield determined to be 85% (107 mg). ^{1}H-NMR (500 MHz, $CDCl_3$): δ= 8.26-8.25 (d, 1H, J = 5.0 Hz), 8.23-8.21 (m, 2H), 8.18-8.16(d, 1H, J = 10.0Hz), 7.90-7.88 (d, 1H, J = 9.0 Hz), 7.72-7.70 (m, 2H), 7.60-7.56 (m, 2H), 7.51-7.48 (m, 2H), 5.57 (s, 2H), 3.97-3.96(d, J = 5.0 Hz, 2H), 2.00-1.94 (m, 1H), 0.95 (s, 3H), 0.94 ppm (s, 3H). ^{13}C-NMR (125 MHz, $CDCl_3$): δ= 155.4, 134.6, 133.0, 132.4, 131.8, 130.8, 130.3, 129.0, 128.0, 127.2, 126.6, 126.5, 123.3, 120.7, 120.6, 120.4, 119.6, 74.3, 67.9, 27.8, 18.9 ppm. HRMS (ESI) exact mass calculated for $[M + Na]^+$ ($C_{26}H_{23}O_3$+Na^+) m/z 406.1539, found m/z 406.1495.

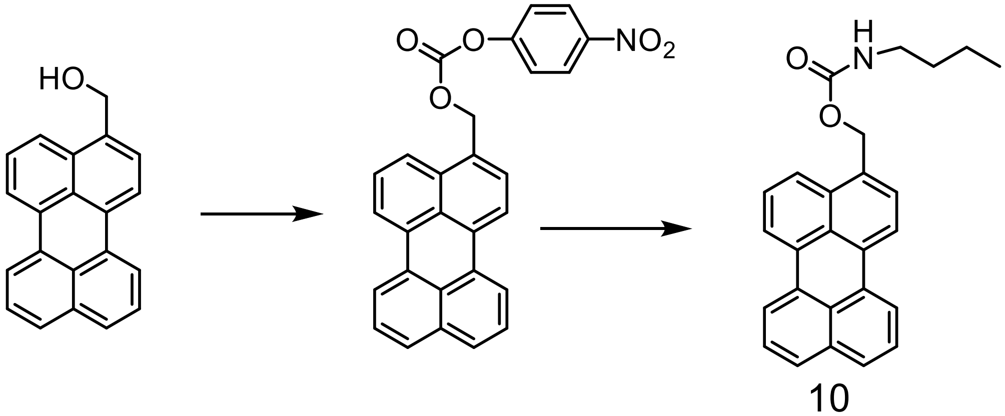

10

Compound 10: 4-benzyl alcohol perylene (140 mg, 0.5 mmol) was dissolved in dry dichloromethane (DCM) (30 mL) and pyridine (50 μL) were mixed. 4-nitrophenyl chloroformate (0.6 mmol) was dissolved in dry DCM (10 mL) and added dropwise to the mixture over 10 min

and then stirred for 2 hours. Next, the butylamine (2.0 mmol) was added and stirred for another 12 hours in room temperature. After the reaction, 50 mL CH_2Cl_2 was added. The solutions were washed with saturated aqueous solution of sodium bicarbonate (30 mL). The organic layer was collected and dried by Na_2SO_4; the solvents were removed under vacuum condition. The raw **10** was purified by silica gel flash column chromatography with DCM/n-hexane =1/1 (v/v) as elute. The shallow yellow band was collected and yield determined to be 45% (91 mg). ^{1}H-NMR (500 MHz, $CDCl_3$): δ= 8.25-8.23 (d, 1H, J =10.0Hz), 8.22-8.19 (m, 2H), 8.16-8.15 (d, 1H, J = 5.0 Hz), 7.89-7.87 (d, 1H, J = 10.0Hz), 7.70-7.69 (d, 2H, J = 5.0Hz), 7.57-7.54 (m, 2H), 7.50-7.47 (m, 2H), 5.50 (s, 2H), 4.74 (s, 1H), 3.24-3.21 (m, 2H), 1.49-1.47 (m, 2H), 1.39-1.32 (m, 2H), 0.94-0.91 ppm (m, 3H). ^{13}C-NMR (125 MHz, $CDCl_3$): δ= 134.6, 133.0, 132.1, 131.8, 131.7, 131.1, 130.9, 129.0, 128.5, 128.1, 128.0, 127.0, 126.6, 123.5, 120.3, 119.7, 65.0, 40.9, 32.0, 19.9, 13.7 ppm. HRMS (ESI) exact mass calculated for $[M + Na]^+$ ($C_{26}H_{23}NO_2 + Na^+$) m/z 404.1626, found m/z 404.1621.

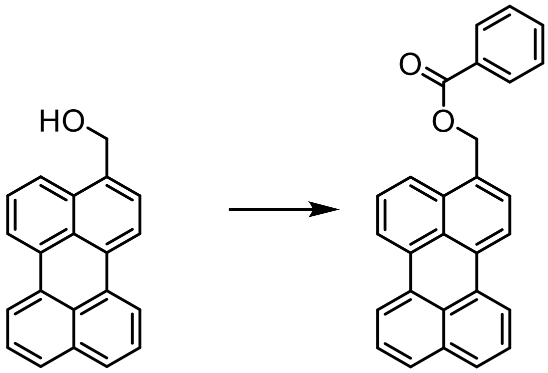

11

Compound 11: 4-benzyl alcohol perylene (140 mg, 0.5 mmol) was dissolved in dry dichloromethane (DCM) (30 mL) and triethylamine (100 μL) were mixed. Benzoyl chloride (0.6 mmol) was dissolved in dry DCM (10 mL) and added dropwise to the mixture over 10 min and then stirred for 12 hours. After the reaction, 50 mL CH_2Cl_2 was added. The solutions were washed with saturated aqueous solution of sodium bicarbonate (30 mL). The organic layer was collected and dried by Na_2SO_4; the solvents were removed under vacuum condition. The raw **11** was purified by silica gel flash column chromatography with DCM/n-hexane =1/1 (v/v) as elute. The shallow yellow band was collected and yield determined to be 80% (154 mg). ^{1}H-NMR (500 MHz, $CDCl_3$): δ= 8.27-8.25 (d, 1H, J = 10.0 Hz), 8.22-8.20 (m, 2H), 8.19-8.16 (m, 2H), 8.09-8.07 (m, 2H), 7.96-7.94 (d, 1H, J = 9.0 Hz), 7.72-7.70 (m, 2H), 7.55-7.53 (d, 2H, J = 10.0 Hz), 7.52-7.49 (m, 2H), 7.44-7.43 (m, 2H), 5.76 ppm (s, 2H). ^{13}C-NMR (125 MHz, $CDCl_3$): δ= 166.6, 134.6, 134.5, 133.1,

133.0, 132.3, 131.9, 131.0, 130.9, 130.6, 130.1, 129.8, 129.1, 128.9, 128.4, 128.3, 128.1,128.0, 127.2, 126.7, 126.6, 123.4, 120.6, 120.5, 120.4, 119.6, 65.3 ppm. HRMS (ESI) exact mass calculated for $[M + Na]^+$ ($C_{28}H_{18}O_2 + Na^+$) m/z 409.1204, found m/z 409.1199.

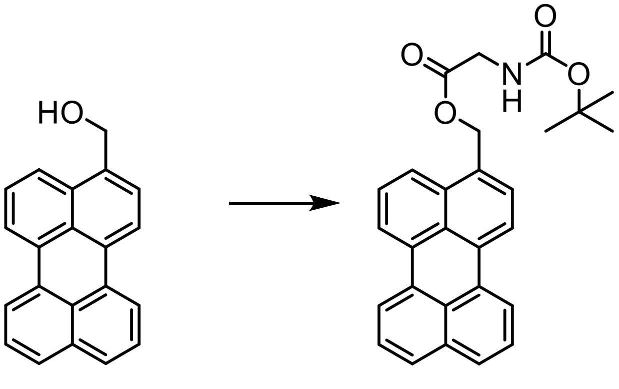

12

Compound 12: 4-benzyl alcohol perylene (140 mg, 0.5 mmol) and Boc-Gly-OH (1.0 mmol) were dissolved in dry dichloromethane (DCM) (30 mL) in 0 °C. Next, the 4-dimethylaminopyridine (DMAP, 0.1 mmol) and *N*, *N*'-dicyclohexylcarbodiimide (DCC, 0.6 mmol) were added to the mixture and then stirred for 12 hours. After the reaction, the solvents were removed under vacuum condition. The raw **12** was purified by silica gel flash column chromatography with DCM/n-hexane =1/1 (v/v) as elute. The shallow yellow band was collected and yield determined to be 78% (171 mg). ^{1}H-NMR (500 MHz, $CDCl_3$): δ = 8.26-8.20 (m, 3H), 8.17-8.15 (d, 1H, J = 10.0 Hz), 7.83-7.81 (d, 1H, J = 10.0Hz), 7.72-7.70 (m, 2H), 7.59-7.54 (m, 2H), 7.51-7.48(m, 2H), 5.59 (s, 2H), 5.01 (s, 1H), 3.99 (s, 2H), 1.44 ppm (s, 9H); ^{13}CNMR (125 MHz, $CDCl_3$) δ = 170.4, 134.6, 133.0, 132.5, 131.9, 131.0, 130.8, 130.2, 129.0, 128.6, 128.5, 128.3, 128.1, 127.3, 126.7, 126.6, 123.2, 120.7, 120.6,120.4, 119.5, 65.5, 42.5, 28.3 ppm. HRMS (ESI) exact mass calculated for $[M + Na]^+$ ($C_{24}H_{25}NO_4 + Na^+$) m/z 462.1681, found m/z 462.1676.

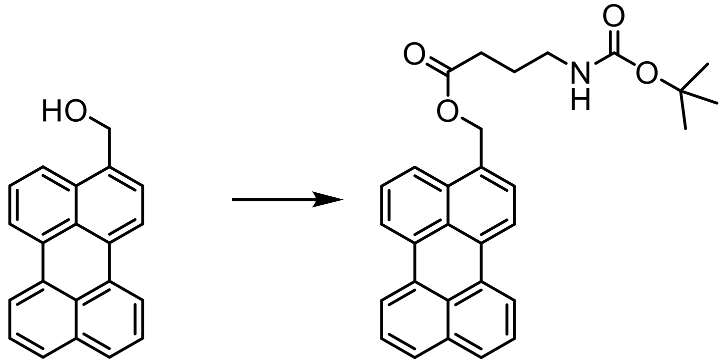

13

Compound 13: 4-benzyl alcohol perylene (140 mg, 0.5 mmol) and Boc-GABA-OH (1.0 mmol) were dissolved in dry dichloromethane (DCM) (10 mL) in 0 °C. Next, the 4-dimethylaminopyridine (DMAP, 0.2 mmol) and *N*,*N*'-dicyclohexylcarbodiimide (DCC, 1.2 mmol) were added to the mixture and then stirred for 12 hours. After the reaction, the solvents were removed under vacuum condition. The raw **13** was purified by silica gel flash column chromatography with DCM/n-hexane =1/1 (v/v) as elute. The shallow yellow band was collected and yield determined to be 69% (156 mg). ^{1}H-NMR (500 MHz, $CDCl_3$): δ = 8.26-8.24 (d, 1H, J = 10.0Hz), 8.23-8.20 (m, 2H), 8.17-8.16 (d, 1H, J = 5.0 Hz), 7.84-7.82 (d, 1H, J = 10.0Hz), 7.72-7.70 (m, 2H), 7.58-7.54 (m, 2H), 7.51-7.48 (m, 2H), 5.21 (s, 2H), 4.58 (s, 1H), 3.16 (s, 2H), 2.45-2.42 (m, 2H), 1.86-1.83 (m, 2H), 1.42 ppm (s, 9H). ^{13}C NMR (125 MHz, $CDCl_3$) δ = 173.2, 155.9, 134.6, 133.0, 132.3, 131.8, 131.1, 130.9, 130.8, 129.0, 128.5, 128.4, 128.2, 128.1, 127.2, 126.7, 126.6, 123.3, 120.6, 120.6, 120.4, 119.6, 64.8, 39.9, 31.6, 28.4, 25.3 ppm. HRMS (ESI) exact mass calculated for $[M + Na]^+$ ($C_{30}H_{29}NO_4 + Na^+$) m/z 490.1994, found m/z 490.1989.

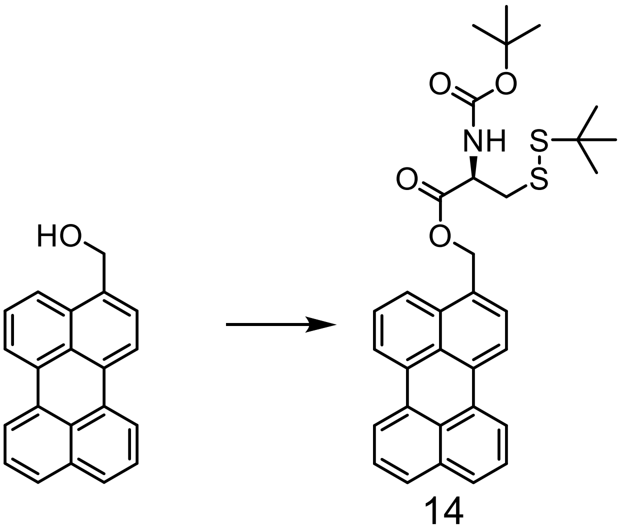

14

Compound 14: 4-benzyl alcohol perylene (140 mg, 0.5 mmol) and Boc-Cys (S*t*Bu)-OH (1.0 mmol) were dissolved in dry dichloromethane (DCM) (10 mL) in 0 °C. Next, the 4-dimethylaminopyridine (DMAP, 0.2 mmol) and *N*,*N*'-dicyclohexylcarbodiimide (DCC, 1.2 mmol) were added to the mixture and then stirred for 12 hours. After the reaction, the solvents were removed under vacuum condition. The raw **14** was purified by silica gel flash column chromatography with DCM/n-hexane =1/1 (v/v) as elute. The yellow band was collected and yield determined to be 65% (186 mg). ^{1}H-NMR (500 MHz, $CDCl_3$): δ = 8.24-8.19 (m, 3H), 8.16-8.13 (m, 1H), 7.83-7.82 (m, 1H), 7.71-7.70 (m, 2H), 7.57-7.48 (m, 4H), 5.58 (s, 2H), 5.43-5.41 (m, 1H),

4.66 (s, 1H), 3.21-3.11 (m, 2H), 1.43 (s, 9H), 1.26 ppm (s, 9H).^{13}C NMR (125 MHz, $CDCl_3$) δ = 170.8, 155.1, 134.6, 133.0, 132.5, 131.8, 131.0, 130.8, 130.1, 129.0, 128.6, 128.4, 128.3, 128.1, 127.2, 126.7, 126.6, 123.3, 120.7, 120.6, 120.4, 119.5, 80.2, 65.9, 48.2, 42.9, 29.7, 28.3 ppm. HRMS (ESI) exact mass calculated for $[M + Na]^+$ ($C_{33}H_{35}NO_4S_2 + Na^+$) m/z 596.1905, found m/z 596.1900.

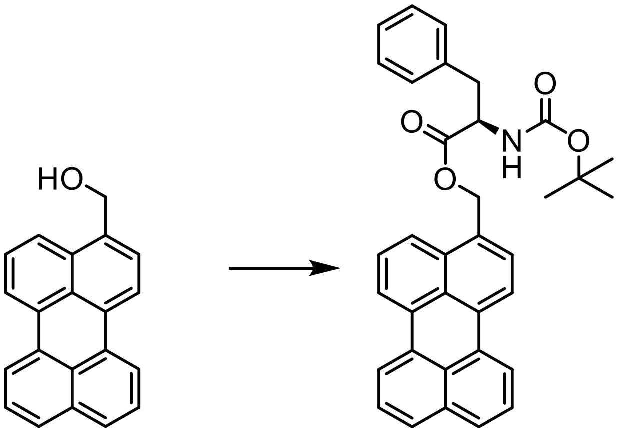

15

Compound 15: 4-benzyl alcohol perylene (140 mg, 0.5 mmol) and Boc-Phen-OH (1.0 mmol) were dissolved in dry dichloromethane (DCM) (10 mL) in 0 °C. Next, the 4-dimethylaminopyridine (DMAP, 0.2 mmol) and *N*,*N*'-dicyclohexylcarbodiimide (DCC, 0.6 mmol) were added to the mixture and then stirred for 12 hours. After the reaction, the solvents were removed under vacuum condition. The raw **15** was purified by silica gel flash column chromatography with DCM/n-hexane =1/1 (v/v) as elute. The shallow yellow band was collected and yield determined to be 65% (163 mg). ^{1}H-NMR (500 MHz, $CDCl_3$): δ = 8.25-8.20 (m, 3H), 8.15-8.13 (d, 1H, J = 10.0 Hz), 7.76-7.70 (m, 3H), 7.55-7.48 (m, 4 H), 7.14 (s, 3H), 6.99 (s, 2H), 5.58-5.49 (m, 2H), 5.02-4.98 (m, 1H), 4.68-4.64 (m, 1H), 3.09-3.04 (m, 2H), 1.40 ppm (s, 9H). ^{13}C NMR (125 MHz, $CDCl_3$) δ = 171.9, 155.1, 135.7,134.6, 133.0, 132.4,131.8, 131.0, 130.8, 130.2, 129.3, 129.0, 128.7, 128.5, 128.3, 128.1, 127.2, 126.9, 126.7, 126.6, 123.2, 120.7, 120.6, 120.4, 119.5, 79.9, 65.4, 54.5, 38.4, 30.3, 28.3 ppm. HRMS (ESI) exact mass calculated for $[M + Na]^+$ ($C_{35}H_{31}NO_4 + Na^+$) m/z 552.2151, found m/z 552.2145.

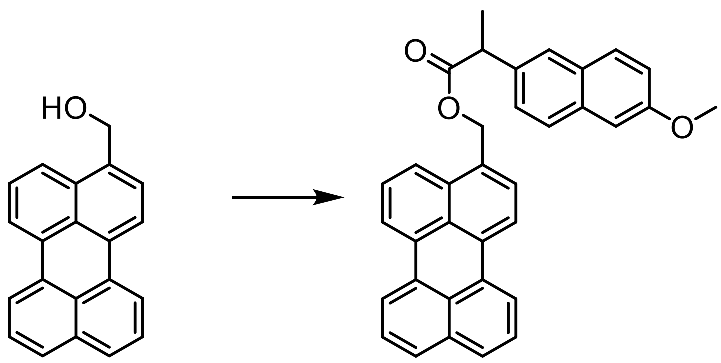

16

Compound 16: 4-benzyl alcohol perylene (140 mg, 0.5 mmol) and naproxen (0.7 mmol) were dissolved in dry dichloromethane (DCM) (10 mL) in 0 ^{o}C. Next, the 4-dimethylaminopyridine (DMAP, 0.2 mmol) and *N*, *N*'-dicyclohexylcarbodiimide (DCC, 0.6 mmol) were added to the mixture and then stirred for 12 hours. After the reaction, the solvents were removed under vacuum condition. The raw **16** was purified by silica gel flash column chromatography with DCM/n-hexane =1/1 (v/v) as elute. The shallow yellow band was collected and yield determined to be 57% (141 mg). ^{1}H-NMR (500 MHz, $CDCl_3$): δ= 8.10-8.07 (m, 2H), 8.05-8.04 (d, 1H, *J* = 5.0Hz), 7.99-7.97 (d, 1H, *J* = 10.0Hz), 7.65-7.58 (m, 5H), 7.53-7.52 (d, 1H, *J* = 5.0Hz), 7.46-7.41 (m, 2H), 7.36-7.34 (m, 2H), 7.22-7.19 (m, 1H), 7.09-7.05 (m, 2H), 5.44 (s, 2H), 3.91-3.90 (m, 1H), 3.87 (s, 3H), 1.66-1.58 ppm (d, *J* = 10.0Hz, 3H). ^{13}C NMR (125 MHz, $CDCl_3$) δ= 174.6,157.6, 135.4, 134.5, 133.7, 132.8, 132.0, 131.5, 131.1, 130.8, 130.7, 129.3, 128.9, 128.4, 128.1, 127.9, 127.1, 126.7, 126.5, 126.4, 126.2, 126.0, 123.3, 120.5, 120.4, 120.2, 119.4, 118.9, 105.5, 65.2, 55.3, 45.5, 18.3 ppm. HRMS (ESI) exact mass calculated for $[M + Na]^+$ ($C_{35}H_{26}O_3 + Na^+$) m/z 517.1780, found m/z 517.1774.

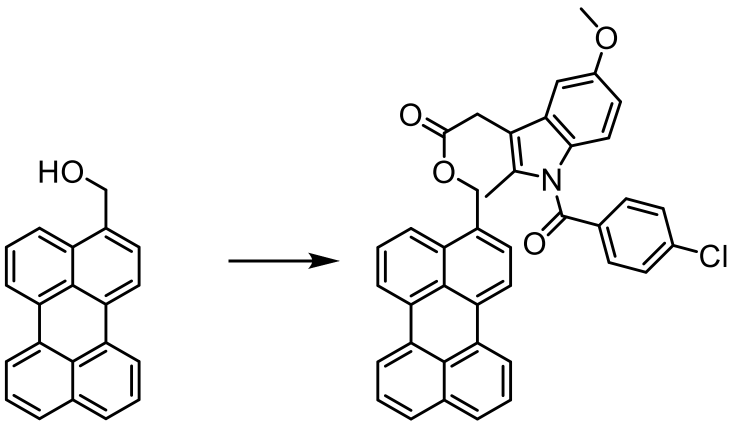

17

Compound 17: 4-benzyl alcohol perylene (140 mg, 0.5 mmol) and indometacin (0.7 mmol) were dissolved in dry dichloromethane (DCM) (10 mL) in 0 °C. Next, the 4-dimethylaminopyridine (DMAP, 0.2 mmol) and *N*, *N*'-dicyclohexylcarbodiimide (DCC, 0.6 mmol) were added to the mixture and then stirred for 12 hours. After the reaction, the solvents were removed under vacuum condition. The raw **17** was purified by silica gel flash column chromatography with DCM/n-hexane =1/1 (v/v) as elute. The shallow yellow band was collected and yield determined to be 53% (164 mg). ^{1}H-NMR (500 MHz, $CDCl_3$): δ = 8.21-8.19 (m, 3H), 8.13-8.11 (d, 1H, J = 10.0Hz), 7.72-7.70 (m, 2H), 7.67-7.65 (d, 1H, J = 10.0Hz), 7.55-7.54 (m, 2H), 7.52-7.49 (m, 3H), 7.39-7.35 (m, 3H), 6.90-6.88 (m, 2H), 6.65-6.63 (m, 1H), 5.52 (s, 2H), 3.72 (s, 2H), 3.66 (s, 3H), 2.29 ppm (s, 3H). ^{13}C NMR (125 MHz, $CDCl_3$) δ = 170.8, 168.3, 156.1, 139.2, 135.8, 134.6, 133.9, 132.9, 132.4, 131.8, 131.1, 131.0, 130.8, 130.7, 130.5, 129.1, 129.0, 128.6, 128.4, 128.3, 128.1, 127.0, 126.7, 126.6, 123.3, 120.7, 120.6, 120.4, 119.5, 115.0, 112.5, 111.9, 101.1, 65.4, 55.5, 30.6, 13.4 ppm. HRMS (ESI) exact mass calculated for $[M + Na]^+$ ($C_{40}H_{28}NO_4Cl + Na^+$) m/z 644.1605, found m/z 644.1609.

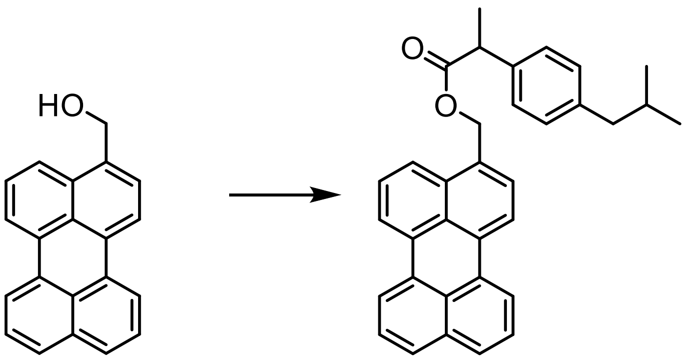

18

Compound 18: 4-benzyl alcohol perylene (140 mg, 0.5 mmol) and ibuprofen (0.7 mmol) were dissolved in dry dichloromethane (DCM) (10 mL) in 0 °C. Next, the 4-dimethylaminopyridine (DMAP, 0.2 mmol) and *N*, *N*'-dicyclohexylcarbodiimide (DCC, 0.6 mmol) were added to the mixture and then stirred for 12 hours. After the reaction, the solvents were removed under vacuum condition. The raw **18** was purified by silica gel flash column chromatography with DCM/n-hexane =1/1 (v/v) as elute. The shallow yellow band was collected and yield determined to be 67% (152 mg). ^{1}H-NMR (500 MHz, $CDCl_3$): δ = 8.21-8.16 (m, 3H), 8.10-8.08 (m, 1H), 7.70-7.68 (d, 2H, J = 10.0Hz), 7.65-7.63 (d, 1H, J = 10.0Hz), 7.50-7.47(m, 2H), 7.44-7.41 (m, 2H), 7.21-7.19 (d, 2H, J = 10.0Hz), 7.10-7.01 (m, 4H), 5.48 (s, 2H), 2.45-2.43 (m, 2H), 1.86-1.82 (m, 1H), 1.53-

1.51 (d, 3H), 0.90 ppm (s, 6H). ^{13}C NMR (125 MHz, $CDCl_3$) δ = 174.7, 140.6, 137.5,134.6, 132.8, 132.0, 131.7, 130.9, 129.5, 129.4, 129.3, 128.9, 128.5, 128.1, 128.0, 127.9, 127.3, 126.9, 126.6, 126.5, 120.4, 120.3, 119.5, 65.0, 45.2, 45.0, 30.2, 22.4, 18.3 ppm. HRMS (ESI) exact mass calculated for $[M + Na]^+$ ($C_{34}H_{30}O_2 + Na^+$) m/z 493.2143, found m/z 493.2138.

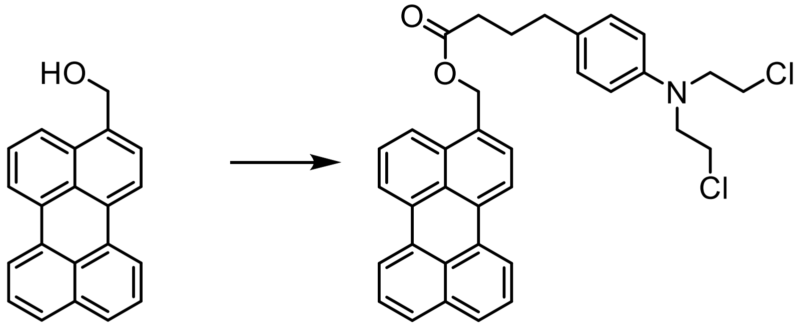

19

Compound 19: 4-benzyl alcohol perylene (140 mg, 0.5 mmol) and chlorambucil (0.7 mmol) were dissolved in dry dichloromethane (DCM) (10 mL) in 0 °C. Next, the 4-dimethylaminopyridine (DMAP, 0.2 mmol) and *N*, *N*'-dicyclohexylcarbodiimide (DCC, 0.6 mmol) were added to the mixture and then stirred for 12 hours. After the reaction, the solvents were removed under vacuum condition. The raw **19** was purified by silica gel flash column chromatography with DCM/n-hexane =1/1 (v/v) as elute. The shallow yellow band was collected and yield determined to be 60% (168 mg). ^{1}H-NMR (500 MHz, $CDCl_3$): δ = 8.26-8.20 (m, 3H), 8.17-8.16 (1H, d, J = 5.0 Hz), 7.86-7.84 (1H, d, J = 10.0Hz), 7.71-7.70 (2H, d, J = 10.0Hz), 7.58-7.55 (m, 2H), 7.52-7.48 (m, 2H), 6.98-6.96 (2H, d, J =10.0Hz), 6.56-6.54 (2H, d, J = 9.0 Hz), 5.52 (s, 2H), 3.67-3.64 (m, 4H), 3.59-3.56 (m, 4H), 2.53-2.50 (m, 2H), 2.40-2.37 (m, 2H), 1.95-1.89 (m, 2H). ^{13}C NMR (125 MHz, $CDCl_3$) δ = 173.5, 144.3, 134.6, 133.0, 131.9, 131.1, 130.9, 130.5, 129.7, 129.1, 128.5, 128.4, 128.2, 128.1, 127.1, 126.7, 126.6, 123.4, 120.6, 120.5, 120.4, 119.6, 112.1, 64.5, 53.6, 40.5, 33.9, 33.7, 26.8 ppm. HRMS (ESI) exact mass calculated for $[M + H]^+$ ($C_{35}H_{31}Cl_2NO_2 + H^+$) m/z 568.1810, found m/z 568.1805.

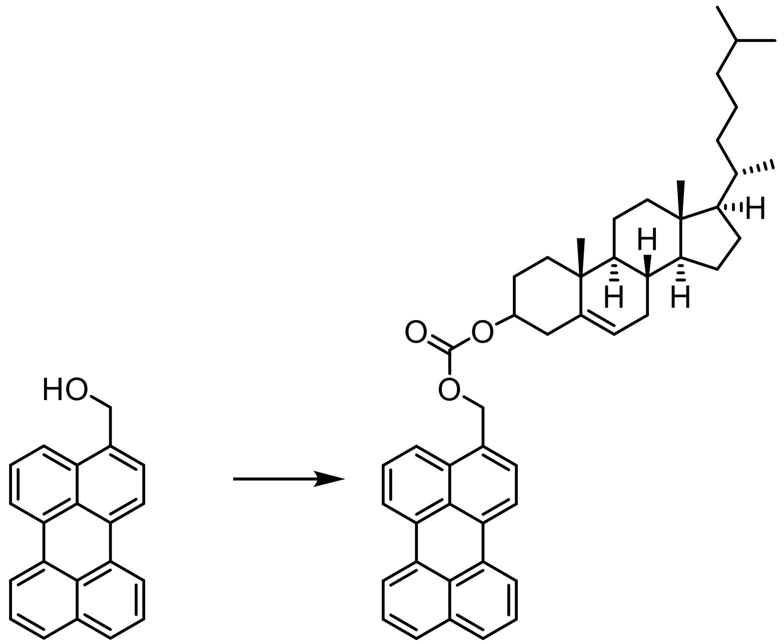

20

Compound 20: 4-benzyl alcohol perylene (140 mg, 0.5 mmol) and pyridine (50 μL) were dissolved in dry dichloromethane (DCM) (10 mL) in 0 °C. Next, the cholesteryl chloroformate. (0.6 mmol) were added to the mixture and then stirred for 12 hours. After the reaction, the solvents were removed under vacuum condition. The raw **20** was purified by silica gel flash column chromatography with DCM/n-hexane =1/1 (v/v) as elute. The shallow yellow band was collected and yield determined to be 21% (72 mg). ^{1}H-NMR (500 MHz, $CDCl_3$): δ = 8.25-8.20 (m, 3H), 8.16-8.15 (d, 1H, J = 5.0 Hz), 7.89-7.87 (d, 1H, J =10.0 Hz), 7.71-7.69 (m, 2H), 7.59-7.55 (m, 2H), 7.51-7.48 (m, 2H), 5.56 (s, 2H), 5.39-5.38 (d, 1H, J =5.0 Hz), 4.55-4.49 (m, 1H), 2.44-2.38 (m, 2H), 2.01-1.95 (m, 3H), 1.88-1.82 (m, 2H), 1.68-1.63 (m, 1H), 1.50-1.43 (m, 5H), 1.36-1.31 (m, 3H), 1.26-1.24 (m, 3H), 1.17-1.04 (m, 8H), 0.99 (s, 4H), 0.92-0.90 (m, 3H), 0.87-0.85 (m, 6H), 0.67 ppm (s, 3H). ^{13}C NMR (125 MHz, $CDCl_3$): δ= 154.6, 139.4, 134.6, 133.0, 132.4, 131.8, 131.1, 130.9, 130.4, 129.0, 128.4, 128.2, 128.1, 127.2, 126.6, 126.5, 123.3, 123.0, 120.7, 120.6, 120.4, 119.6, 78.2, 67.7, 56.7, 56.1, 50.0, 42.3, 39.7, 39.5, 38.0, 36.9, 36.5, 36.2, 35.8, 31.9, 31.8, 28.2, 27.7, 24.3, 23.8, 22.8, 22.6, 21.0, 19.3, 18.7, 11.9 ppm. HRMS (ESI) exact mass calculated for $[M + Na]^+$ ($C_{49}H_{58}O_3 + Na^+$) m/z 717.4284, found m/z 717.4278.

Supplementary Tables

Supplementary Table 1. The photophysical parameters of Sens and PPGs[a].

	λ_{abs}[b]	λ_{em}[c]	ξ (10^4 M^{-1} cm^{-1})[d]	T_1 (eV)[e]	S_1 (eV)[f]	τ_T (μs)[g]
**PtTNP**	687	866	13.2	1.43	–[p]	11.0[h]
**PdTPBP**	630	790	11.0	1.55	–[p]	223.7[i]
**PtOEP**	533	647	7.0	1.92	–[p]	90[j]
**Ir(ppy)$_3$**	377	513	1.7	2.40	–[p]	1.6[k]
**BDP** (**7**)	517	537	7.3	1.49[l]	2.31	–[p]
**Py** (**8**)	440	445	4.0	1.52 [m]	2.78	–[p]
**An** (**2**)	366	411	1.1	1.77 [n]	3.02	–[p]
**Cou** (**1**)	360	460	0.62	2.18[o]	2.52	–[p]

[a] in toluene, 10 μM; [b] maximum absorption wavelength; [c] maximum emission wavelength; [d] molar extinction coefficient; [e] the energy level of triplet excited state (calculated with the maximum emission wavelength of phosphorescence); [f] the energy level of singlet excited state (calculated with the maximum emission wavelength of fluorescence); [g] triplet excited lifetime of Sen; [h] reference 3; [i] reference 4; [j] reference 5; [k] reference 6; [l] reference 7; [m] reference 8; [n] reference 9; [o] reference 10; [p] not applicable.

Supplementary Table 2. A summary of the singlet excited/triplet excited state energy levels of PPGs and Sens

Sens:	PtTNP	PdTPBP	PtOEP	Ir(ppy)$_3$	PPGs:	BDP	Py	An	Cou
S_1 (eV)	–	–	–	–		2.31	2.78	3.02	2.52
T_1 (eV)	1.43	1.55	1.92	2.4		1.49	1.52	1.77	2.18
$2\times T_1$-S_1 (eV)						0.69	0.26	0.53	1.85

Supplementary Table 3. The combination of Sen and PPGs in this study.

	PtTNP	PdTPBP	PtOEP	$Ir(ppy)_3$
BDP	√	√	×	×
Py	√	√	√	×
An	×	×	√	×
Cou	×	×	×	√

"√": a TTAP pair; "×": not a TTAP pair.

Supplementary Table 4. The photolytic reaction for $Ir(ppy)_3$ and Cou (compound **1**).

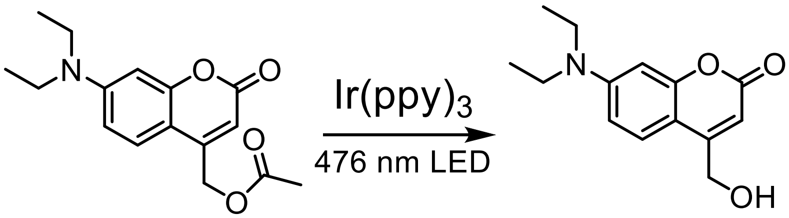

Cou (1)

Entry	Condition	Reaction time	Yield (%)[a]
1	light; air	20 min	4
2	dark, argon;	20 min	No reaction
3	light, Cou (compound **1**) only, argon;	20 min	No reaction
4	light; argon;	20 min	73.6

[a] Measured by HPLC

The photolysis yield of Cou (compound 1) at some condition without $Ir(ppy)_3$ under 365 nm illumination was measured. The photolytic yield is 79.8 %

Supplementary Table 5. The photolytic reaction for PtOEP and An (compound **2**).

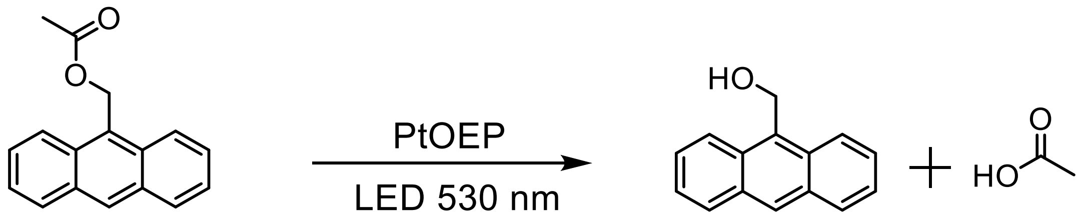

Entry	Condition	Reaction time	Yield (%) [a]
1	Light, air	1 h	Trace
2	Dark, argon	1 h	No reaction
3	An (compound **2**) only, light, argon	1 h	No reaction
4	Light, argon	1 h	83.2

[a] Measured by HPLC.

Supplementary Table 6. The Photolytic reaction for PtOEP and Py (compound **8**).

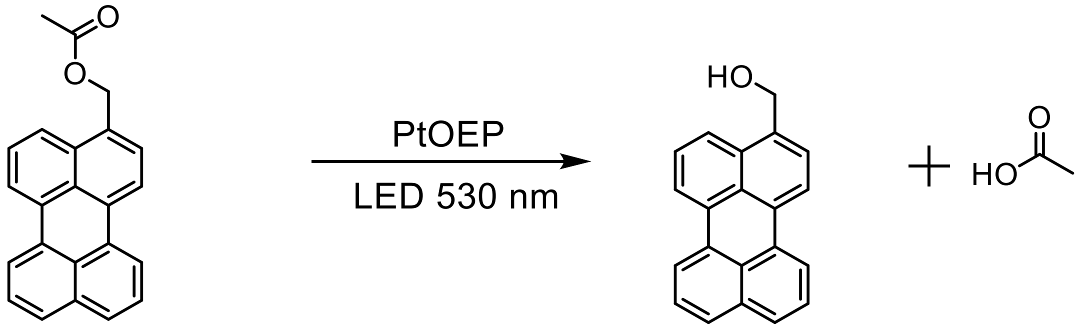

Entry	Condition	Reaction time	Yield (%) [a]
1	Light, air	1 h	2
2	Dark, argon	1 h	No reaction
3	Py (compound **8**) only, light, argon	1 h	No reaction
4	Light, argon	1 h	70.2
5	Light, argon, 520 nm long pass filter	1 h	61.3

[a] Measured by HPLC.

Supplementary Table 7. The photolytic reaction for PdTPBP: Py (compound **8**).

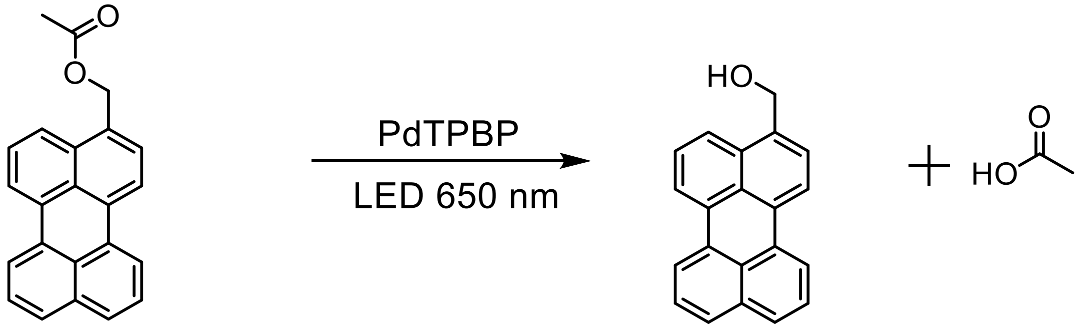

Entry	Condition	Reaction time	Yield (%) [a]
1	Light, air	1 h	No reaction
2	Dark, argon	1 h	No reaction
3	Py (compound **8**) only, light, argon	1 h	No reaction
4	Light, argon	1 h	87.1

[a] Measured by HPLC.

Supplementary Table 8. The photolytic reaction for PdTPBP: BDP (compound **7**).

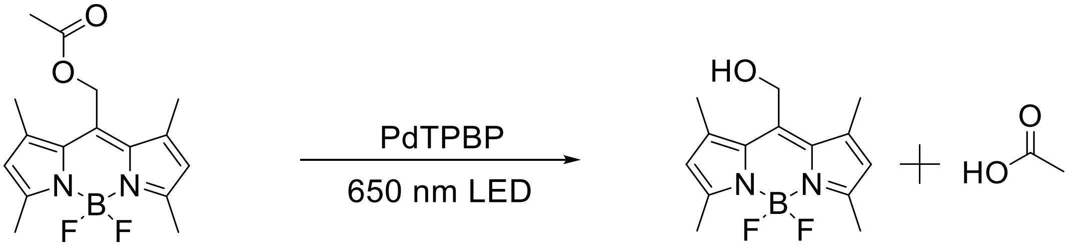

Entry	Condition	Reaction time	Yield (%) [a]
1	Light, air	1 h	No reaction
2	Dark, argon	1 h	No reaction
3	BDP (compound **7**) only, light, argon	1 h	No reaction
4	Light, argon	1 h	76.0

[a] Measured by HPLC.

Supplementary Table 9. The photolytic reaction for PtTNP: Py (compound **8**).

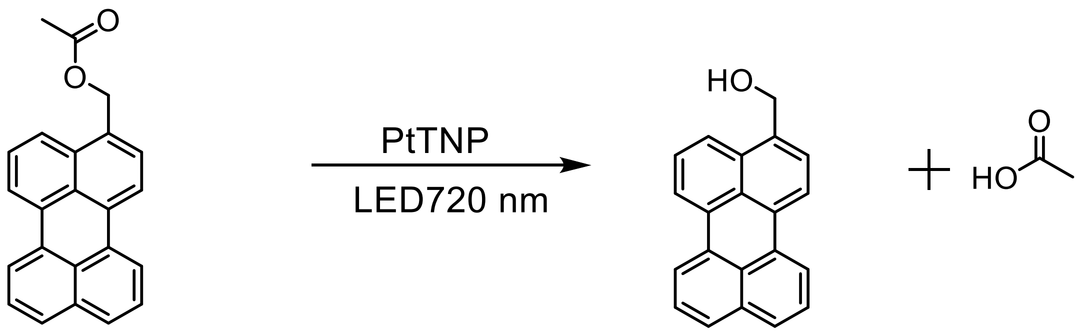

Entry	Condition	Reaction time	Yield (%) [a]
1	Light, air	1 h	No reaction
2	Dark, argon	1 h	No reaction
3	Py (compound **8**) only, light, argon	1 h	No reaction
4	Light, argon	1 h	14.7
5	Light, argon	2 h	24.3 %

[a] Measured by HPLC.

Supplementary Table 10. The photolytic reaction for PtTNP: BDP (compound **7**).

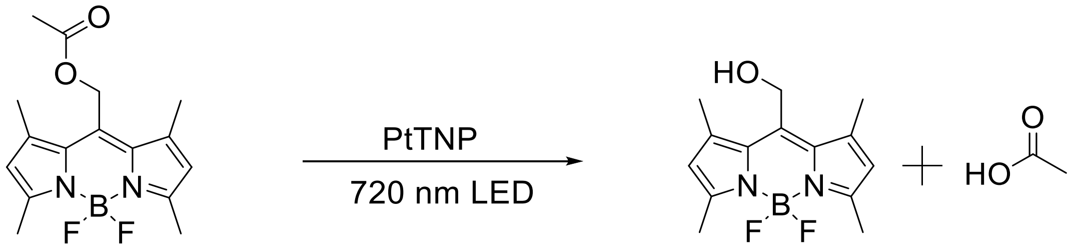

Entry	Condition	Reaction time	Yield (%) [a]
1	Light, air	1 h	No reaction
2	Dark, argon	1 h	No reaction
3	BDP (compound **7**) only, light, argon	1 h	No reaction
4	Light, argon	1 h	17.7

[a] Measured by HPLC.

Supplementary Table 11. The photolytic reactions for Py (compound **8**) at room light and blue light illumination.

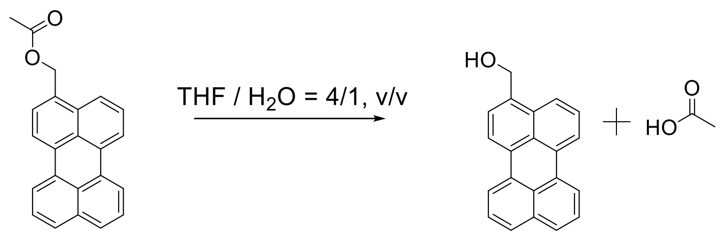

Entry	Condition	Reaction time	Yield (%) [a]
1	Py (compound **8**) only, air, dark	60 min	No reaction
2	Py (compound **8**) only, air, room light	60 min	No reaction
3	PdTPBP, Py (compound **8**), air, room light	60 min	No reaction
4	PdTPBP, Py (compound **8**), argon, room light	60 min	No reaction
5	Py (compound **8**) only, air, blue light (445 nm)	60 min	59.0

[a] HPLC yield.

Supplementary Table 12. The photocleavage quantum yield (**Φ_p**) and photolytic efficiency of TTAP ($\boldsymbol{\varepsilon_2} \times \boldsymbol{\Phi_p} \times \Phi_{TTET} \times \Phi_{TTA}$).

	**Φ_p** [a]	$\boldsymbol{\varepsilon_2} \times \boldsymbol{\Phi_p} \times \Phi_{TTET} \times \Phi_{TTA}$ ***TTAP*** (($\boldsymbol{\eta}_{(TTAP)}$))
BDP (compound **7**)	9.9×10^{-4} [b]	12.3[c]/0.53[d]
Py (compound **8**)	0.093[e]	7.1[d]<br>679[c]<br>708[f]
An (compound **2**)	0.089[g]	1255 [f]
Cou (compound **1**)	0.13[h]	—

[a] photocleavage quantum yield of PPGs; [b] reference 11; [c] PdTPBP as sensitizer; [d] PtTNP as photosensitizer; [e] reference 12; [f] PtOEP as sensitizer; [g] reference 13; [h] reference 14.

Supplementary Table 13. Stern−Volmer quenching constant, bimolecular quenching constant of different TTAP pairs and triplet excited lifetime of photosensitizer (τ_T).

**Pair:**	**PdTPBP: BDP**	**PdTPBP:Py**	**PtOEP: Py**	**PtOEP:An**	**$Ir(ppy)_3$: Cou**
k_{sv} (**M^{-1}**) [a]	2.40×10^5	2.43×10^5	2.62×10^5	2.19×10^5	3.13×10^3
k_q (**$M^{-1}S^{-1}$**) [b]	1.08×10^9	1.09×10^9	2.9×10^9	2.43×10^9	2.34×10^9
**Photosensitizer**	PdTPBP	PtOEP		$Ir(ppy)_3$	
τ_T (μs)	222	91		1.5	

[a] Stern−Volmer quenching constant; [b] bimolecular quenching constant, $k_{sv} = k_q\tau_T$. BDP (compound **7**), Py (compound **8**), An (compound **2**) and Cou (compound **1**)

Supplementary Table 14. The photolytic reaction for PdTPBP: compound **19** under far red LED irradiation.

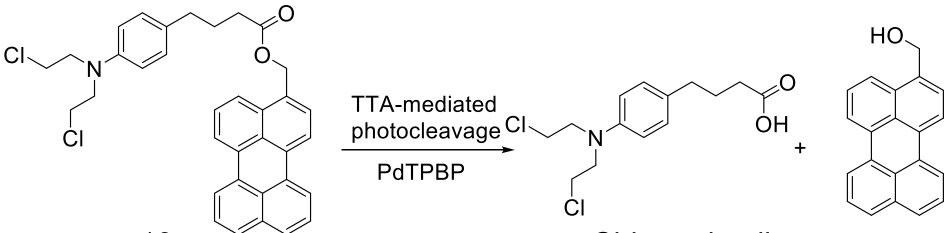

19 Chlorambucil

Entry	Condition	Reaction time (h)	Yield [a]
1	Compound **19** only; red light;	2	None
2	Compound **19** only; argon; red light, 20 mW/cm^2	2	None
3	PdTPBP, compound **19**; argon; red light, 20 mW/cm^2	0.5	47
4	PdTPBP, compound **19**; argon; red light, 10 mW/cm^2	1	26
5	PdTPBP, compound **19**; argon; red light, 20 mW/cm^2	1	72.8
6	PdTPBP, compound **19**; argon; red light, 20 mW/cm^2	2	67.0
7	PdTPBP, compound **19**; air, red light, 20 mW/cm^2	2	7
8	PdTPBP, compound **19**; argon, dark;	2	None

[a] HPLC yield.

Supplementary Table 15. The photolytic reaction for PtTNP: pro-chlorambucil (compound **19**) under 720 nm LED irradiation.

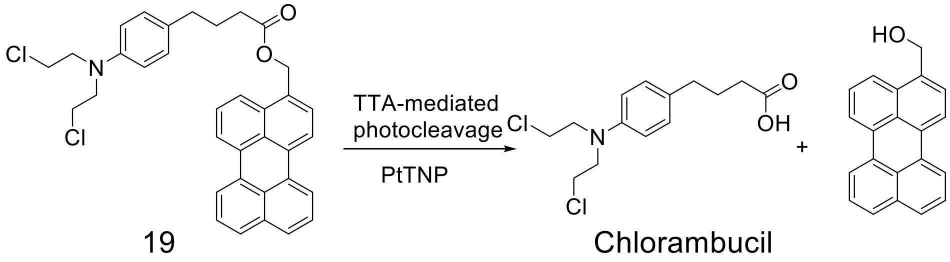

Entry	Condition	Reaction time (h)	Yield [a]
1	Compound **19** only; 720 nm LED;	1	No reaction
2	Compound **19** only; argon; 720 nm LED;	1	No reaction
3	PtTNP, compound **19**; argon; 720 nm LED;	1	30.4
4	PtTNP, compound **19**; air, 720 nm LED;	1	No reaction
6	PtTNP, compound **19**; argon, dark;	1	No reaction

[a]HPLC yield.

Supplementary Table 16. Blood biochemistry and complete blood panel analysis of mice.[a]

	Normal range	Control	3 h	6 h	12 h	24 h	48 h	96 h
WBC (K/μL)	1.8-10.7	6.42	9.78	10.08	8.90	6.22	6.04	6.4
NE (K/μL)	0.1-2.4	2.29	2.00	2.07	1.46	2.27	1.6	0.12
LY (K/μL)	0.9-9.3	3.59	6.55	6.70	4.82	3.38	4.08	4.0
MO (K/μL)	0.0-0.4	0.39	0.16	0.28	0.38	0.28	0.27	0.02
EO (K/μL)	0.0-0.2	0.11	0.05	0.02	0.18	0.08	0.07	0.01
BA (K/μL)	0.0-0.2	0.02	0.01	0.01	0.06	0.01	0.01	0.02
RBC (M/μL)	6.36-9.42	8.83	9.31	8.90	8.95	9.36	9.07	8.76
HGB (g/dL)	11.0-15.1	13.3	13.5	13.7	13.6	13.6	12.8	13.1
HCT (%)	35.1-45.4	44.1	40.6	45.1	43.7	41.3	38.7	36.3
MCV (fL)	45.4-60.3	55.0	54.4	55.7	54.0	54.8	53.7	53.8
MCH (pg)	14.1-19.3	14.5	14.5	16.8	17.7	14.5	14.1	14.6
MCHC (K/μL)	30.2-34.2	31.6	31.7	32.9	33.3	32.5	32.2	30.2
RDW (K/μL)	12.4-27.0	16.8	18.3	17.2	17.7	16.8	17.5	17.4
PLT (K/μL)	592-2972	630	669	649	743	605	671	778
MPV (fL)	5.0-20.0	5.6	6.1	6.2	5.3	5.6	5.7	6.2

[a] The Balb/c mice were *i.v.*-injected with TTAP NPs (100 μg mL^{-1}, 150 μL) and then sacrificed at the various time points (3, 6, 12, 24, 48, 96 hour) after injection. Another four Balb/c mice were used as the control. Before the mice were euthanatized, blood samples (~ 0.5 mL) were collected for blood panel analyses and blood chemistry tests. White blood cell (WBC), Neutrophils (NE), Lymphocytes (LY), Monocytes (MO), Eosinophils (EO), Basophils (BA), Red blood cell (RBC), Hemoglobin (HGB), Hematocrit (HCT), Mean corpuscular volume (MCV), mean corpuscular hemoglobin (MCH), Mean corpuscular hemoglobin concentration (MCHC), Red blood cell distribution width (RDW), Platelet Thrombocyte (PLT), Mean platelet volume (MPV).

Supplementary Figures

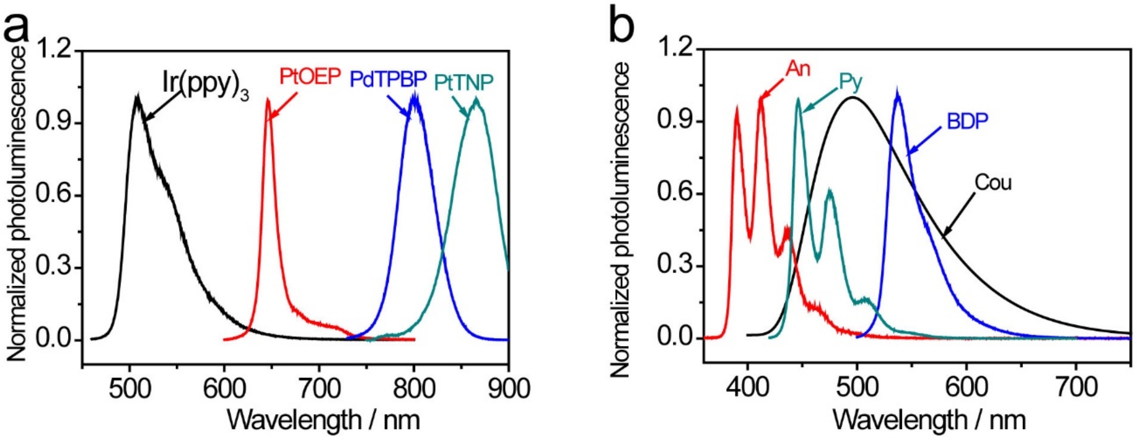

Supplementary Figure 1. (a) Normalized photoluminescence emission spectra of Ir(ppy)$_3$ (λ_{ex} = 410 nm), PtOEP (λ_{ex} = 530 nm), PdTPBP (λ_{ex} = 630 nm) and PtTNP (λ_{ex} = 700 nm); (b) Normalized photoluminescence emission spectra of Cou (compound **1**, λ_{ex} = 360 nm), An (compound **2**, λ_{ex} = 360 nm), Py (compound **8**, λ_{ex} = 410 nm) and BDP (compound **7**, λ_{ex} = 470 nm).

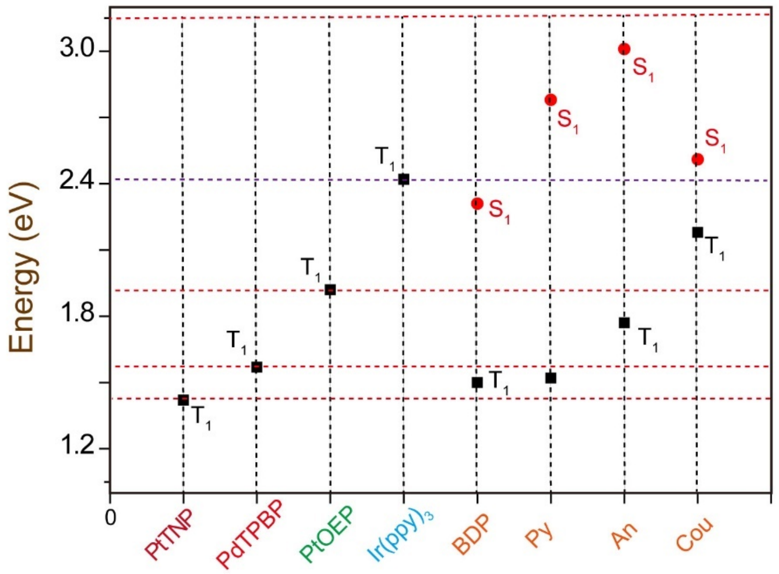

Supplementary Figure 2. A plot comparison of triple excited state and single excited state of Sens and PPGs.

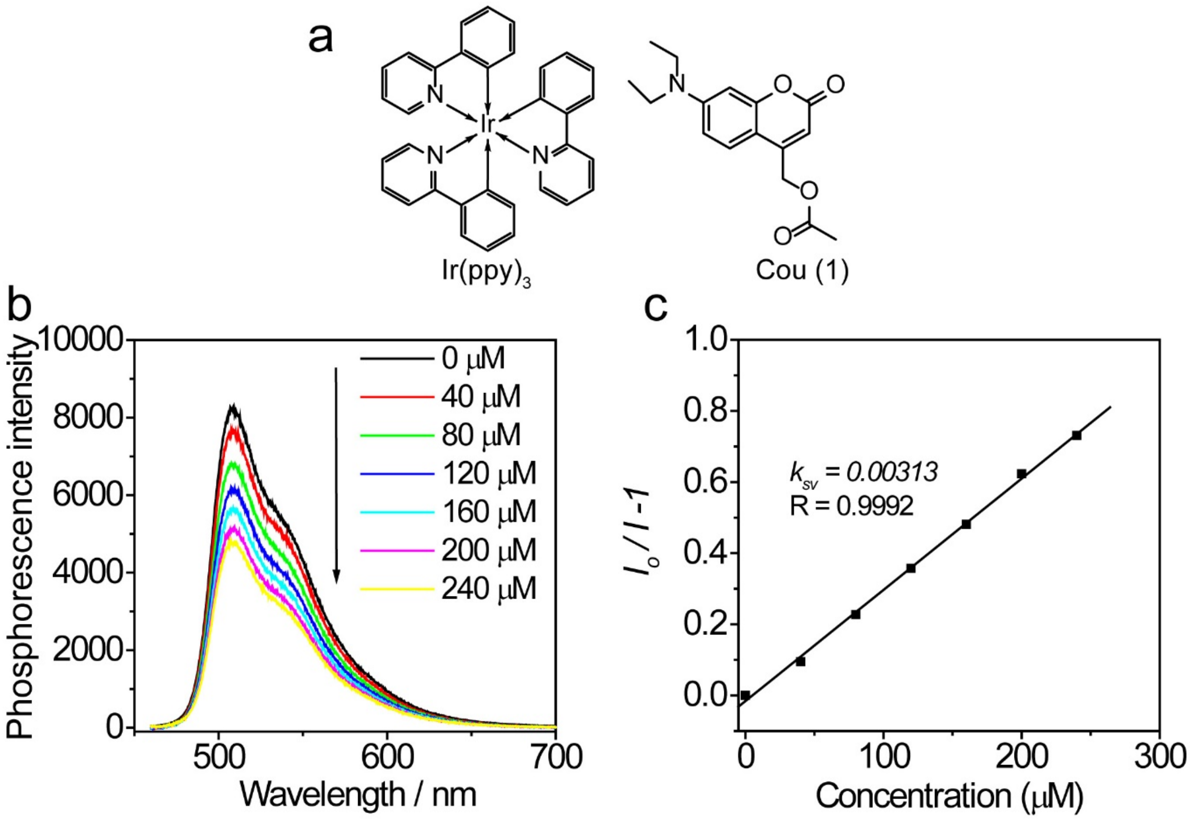

Supplementary Figure 3. (a) Molecular structures of $Ir(ppy)_3$ and Cou (compound **1**); (b) phosphorescence intensity quenching of $Ir(ppy)_3$ (10 μM) in the presence of different concentration of Cou (compound **1**), λ_{ex} = 410 nm; (c) Stern-Volmer plots generated from the phosphorescence intensity quenching of $Ir(ppy)_3$ as a function of Cou (compound **1**) concentration.

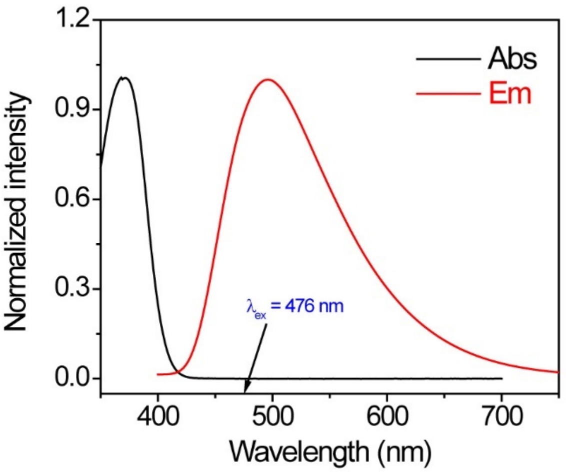

Supplementary Figure 4. Normalized UV-vis absorption and emission spectra of Cou (compound **1**).The fluorescence emission wavelength of compound **1** is very broad from 420 nm to 700 nm (peak at 498 nm), overlapping the excitation wavelength used for Ir(ppy)$_3$ photosensitizer (476 nm). Therefore, in this case, it is unlikely to separate excitation light from our upconverted luminescence to obtain upconversion spectra.

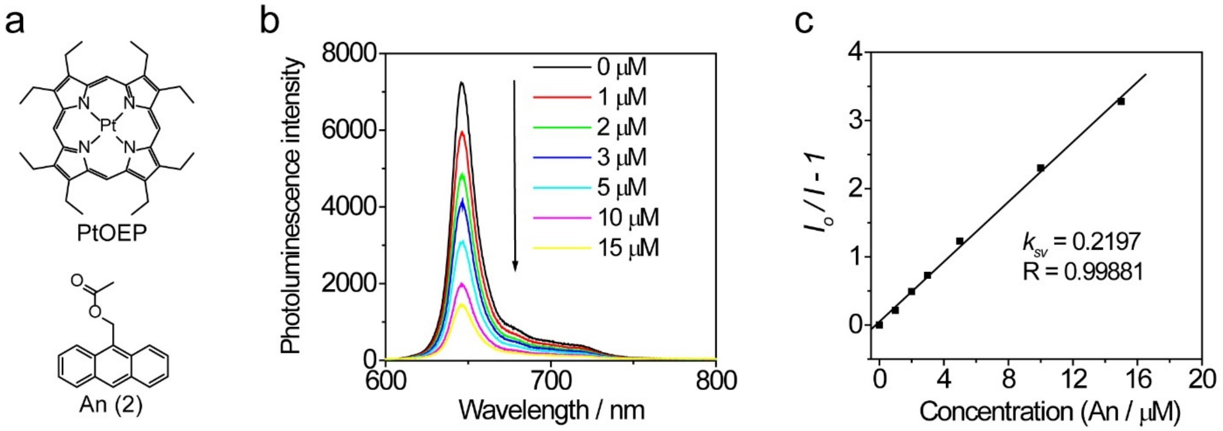

Supplementary Figure 5. (a) Molecular structures of PtOEP and An (compound **2**); (b) phosphorescence intensity quenching of PtOEP (10 μM) in the presence of different concentration of An (compound **2**), λ_{ex} = 530 nm; (c) Stern-Volmer plots generated from the phosphorescence

intensity quenching of PtOEP as a function of An (compound **2**) concentration. In toluene.

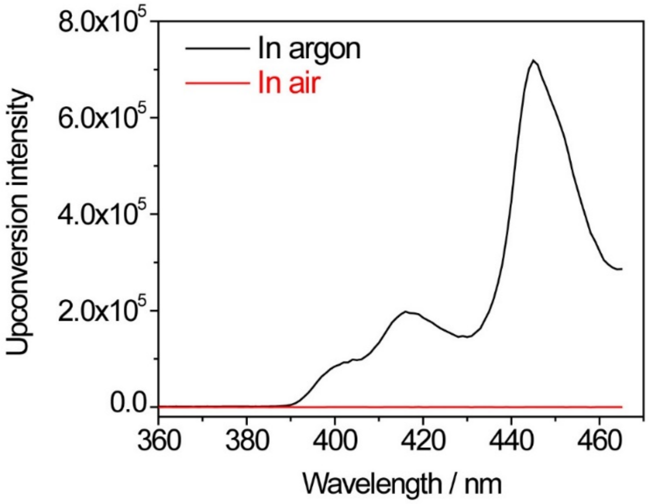

Supplementary Figure 6. Upconversion emission spectra of PtOEP (10 μM) and An (compound **2**) (500 μM). long pass filter (>520 nm) was used to cut off the short-wavelength, λ_{ex} = 530 nm, 20 mW cm^{-2}, in toluene.

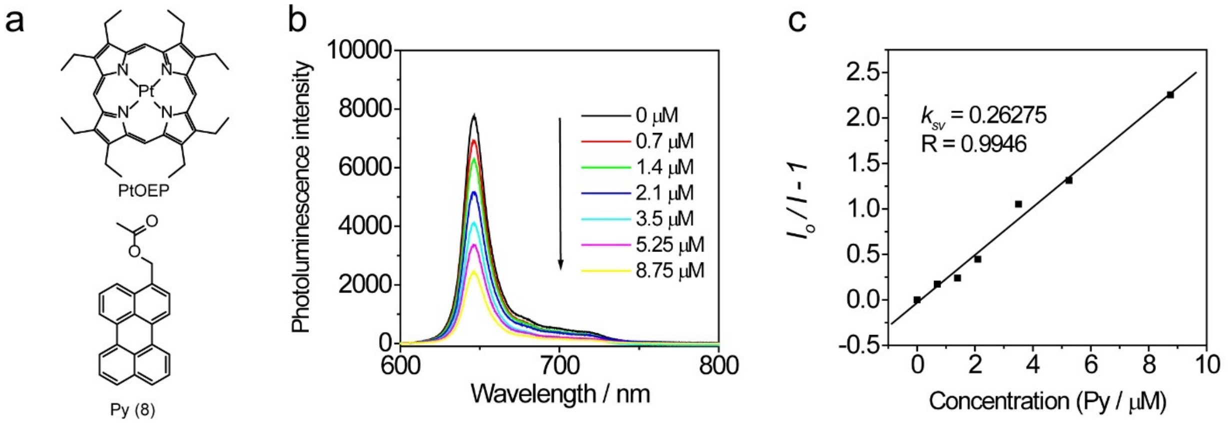

Supplementary Figure 7. (a) Molecular structures of PtOEP and Py (compound **8**); (b) phosphorescence intensity quenching of PtOEP (10 μM) in the presence of different concentration of Py (compound **8**), λ_{ex} = 530 nm; (c) Stern-Volmer plots generated from the phosphorescence intensity quenching of PtOEP as a function of Py (compound **8**) concentration, in toluene.

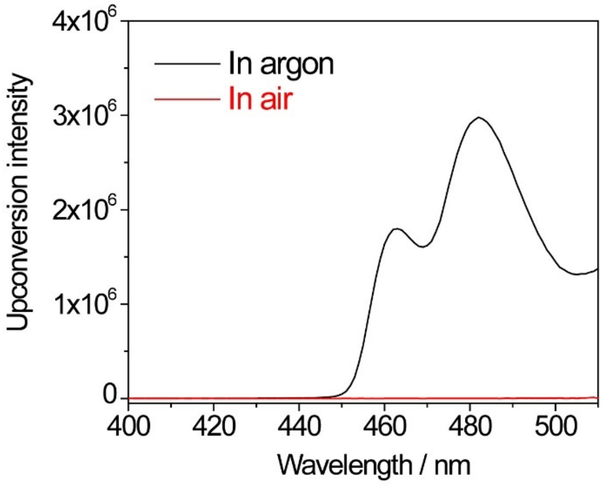

Supplementary Figure 8. Upconversion emission spectra of PtOEP (10 μM) and Py (compound **8**) (500 μM) in air and in argon. Long pass filter (>520 nm) was used to cut off the short-wavelength, in toluene, λ_{ex} = 530 nm, 20 mW cm^{-2}.

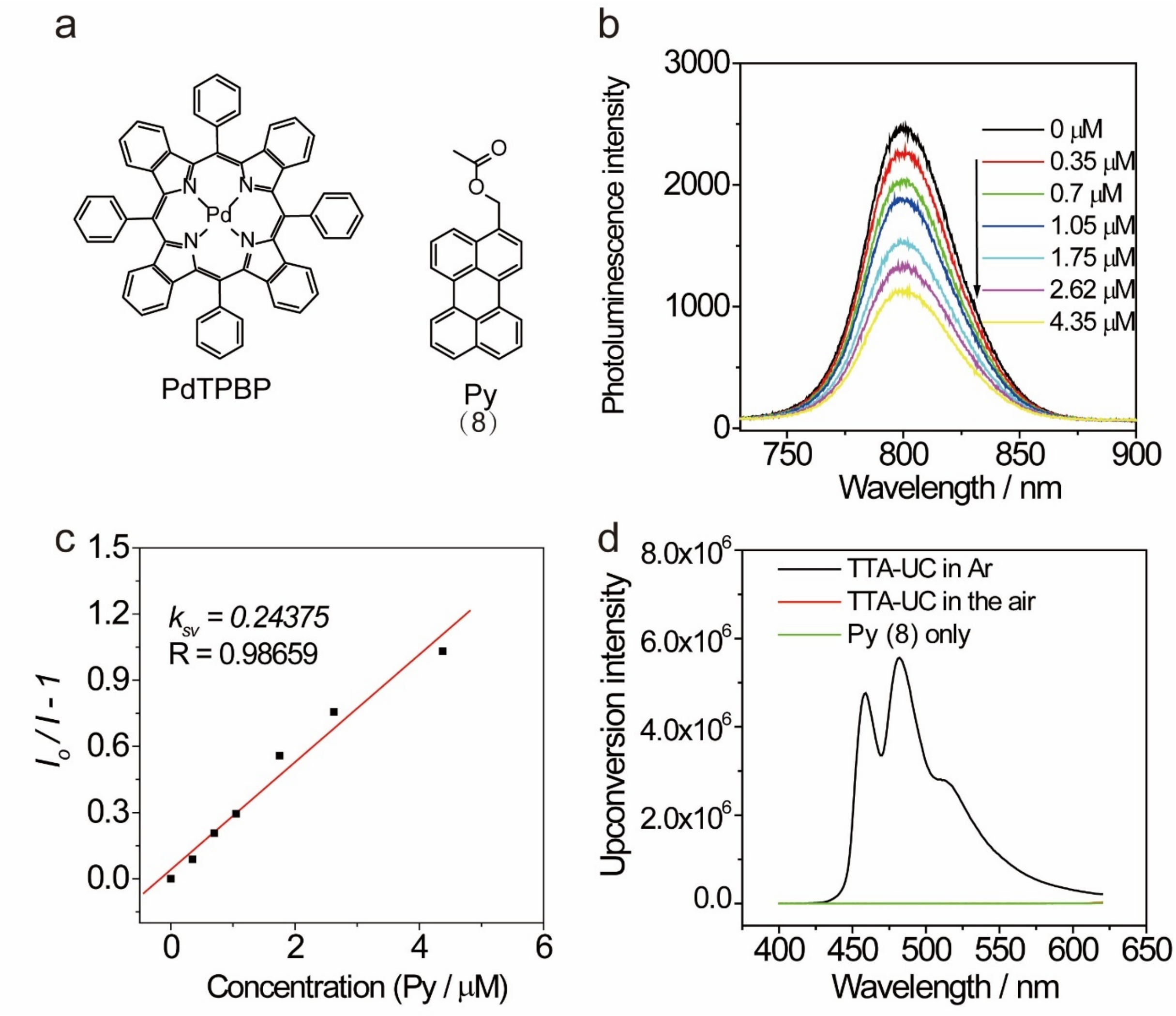

Supplementary Figure 9. (a) Molecular structures of PdTPBP and Py (compound **8**); (b) phosphorescence intensity quenching of PdTPBP (10 μM) in the presence of different concentration of Py (compound **8**), λ_{ex} = 635 nm; (c) Stern-Volmer plot generated from the phosphorescence intensity quenching of PdTPBP as a function of Py (compound **8**) concentration; (d) the upconversion emission spectra of PdTPBP and Py (compound **8**) paired in the air, in argon and in the absence of PdTPBP, λ_{ex} = 650 nm, 100 mW cm^{-2}, in toluene.

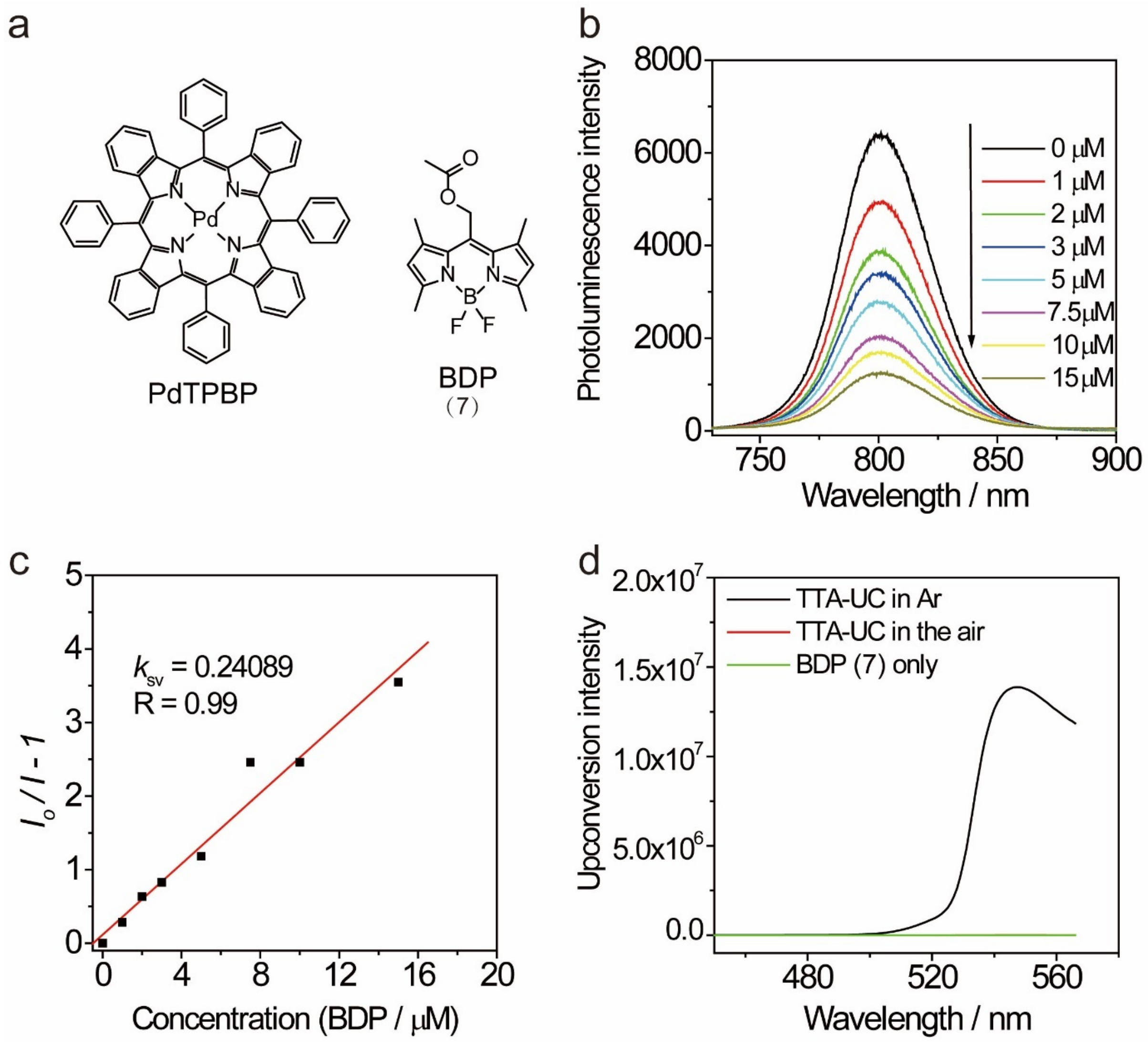

Supplementary Figure 10. (a) Molecular structures of PdTPBP and BDP (compound **7**); (b) phosphorescence intensity quenching of PdTPBP (10 μM) in the presence of different concentration of BDP (compound **7**), λ_{ex} = 635 nm; (c) Stern-Volmer plot generated from the phosphorescence intensity quenching of PdTPBP as a function of BDP (compound **7**) concentration; (d) the upconversion emission spectra of PdTPBP and BDP (compound **7**) paired in the air, in argon and in the absence of PdTPBP, λ_{ex} = 650 nm, 100 mW cm^{-2}, in toluene.

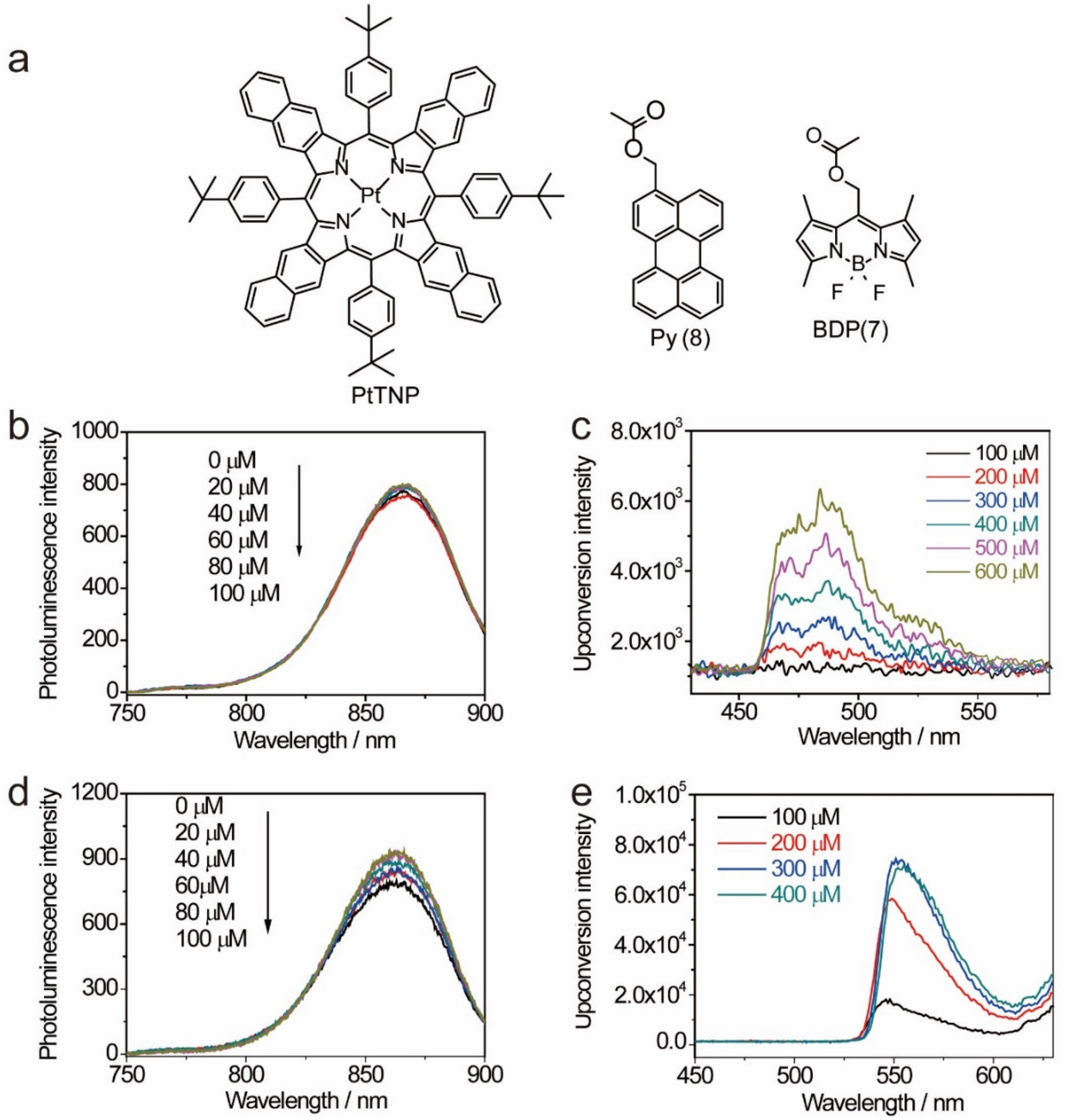

Supplementary Figure 11. (a) Molecular structures of PtTNP, Py (compound **8**) and BDP (compound **7**); (b) phosphorescence intensity quenching of PtTNP (10 μM) in the presence of different concentration of Py (compound **8**), λ_{ex} = 700 nm; (c) The upconversion emission spectra of PtTNP and Py (compound **8**) in the different concentration of Py (compound **8**), λ_{ex} = 720 nm, 100 mW cm^{-2}, in toluene; (d) phosphorescence intensity quenching of PtTNP (10 μM) in the presence of different concentration of BDP (compound **7**), λ_{ex} = 700 nm; (e) the upconversion emission spectra of PtTNP and BDP (compound **7**) in the different concentration of BDP (compound **7**), λ_{ex} = 720 nm, 100 mW cm^{-2}, in toluene.

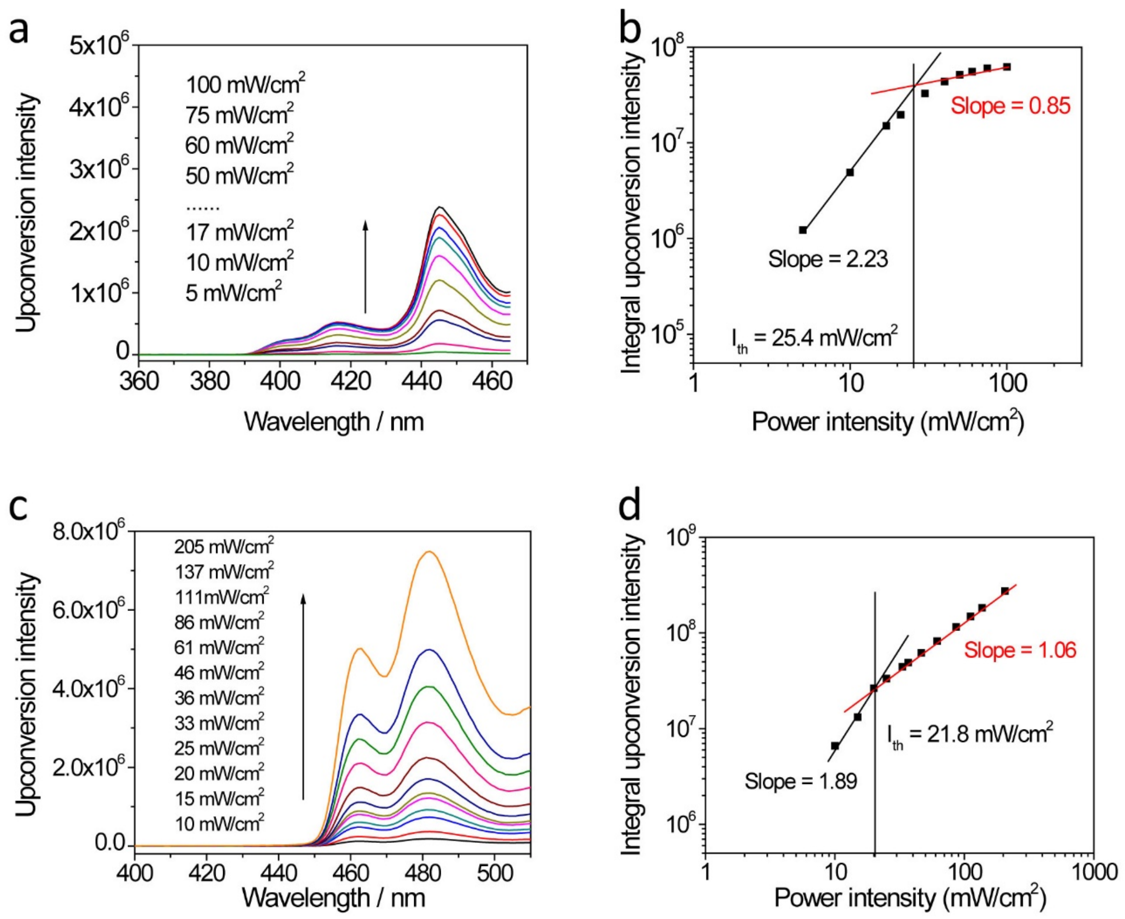

Supplementary Figure 12. (a) The upconversion emission spectra of the TTA-UC system of PtOEP (10 μM) and An **(**compound **2)** (500 μM) under argon with various excitation intensities (λ_{ex} = 530 nm). (b) The dependence of the TTA-UC intensity of the solution of PtOEP and An (compound **2)** on the various incident power density. The results fit the lines that have slopes of 2.23 (black, below) and 0.85 (red, above) in the low- and high-power regions, respectively. I_{th} is 25.4 mW cm^{-2}. (c) Upconversion emission spectra of the TTA-UC system of PtOEP (10 μM) and Py (compound **8)** (500 μM) under argon with various excitation intensities (λ_{ex} = 530 nm). (d) The dependence of the TTA-UC intensity of the solution of PtOEP and Py (compound **8)** on the various incident power density. The results fit the lines that have slopes of 1.89 (black, below) and 1.06 (red, above) in the low- and high-power regions, respectively. I_{th} is 21.8 mW cm^{-2}.

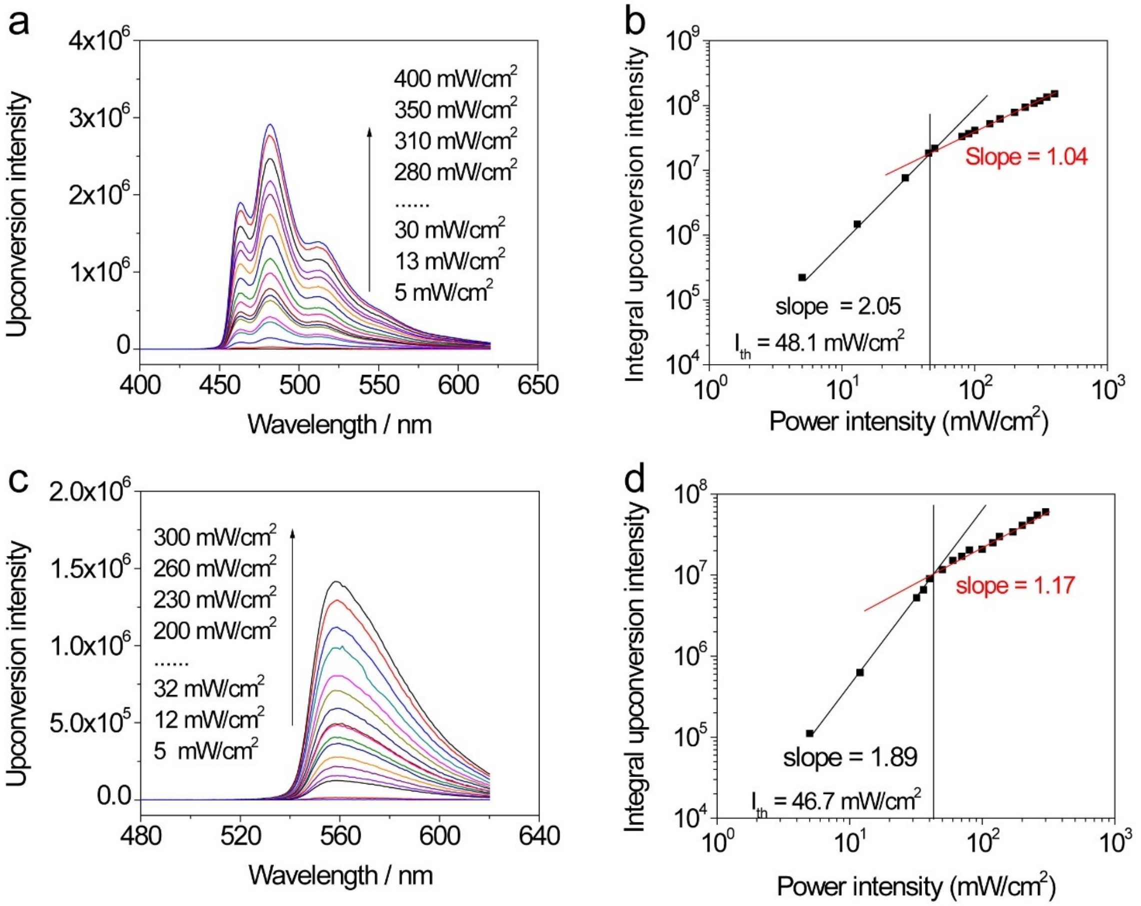

Supplementary Figure 13. (a) Upconversion emission spectra of the TTA-UC system of PdTPBP (10 μM) and Py (compound **8)** (500 μM) under argon with various excitation intensities (λ_{ex} = 650 nm). (b) The dependence of the TTA-UC intensity of the solution of PdTPBP and Py (compound **8)** on the various incident power density. The results fit the lines that have slopes of 2.05 (black, below) and 1.04 (red, above) in the low- and high-power regions, respectively. I_{th} is 48.1 mW cm^{-2}. (c) Upconversion emission spectra of the TTA-UC system of PdTPBP (10 μM) and BDP (compound **7)** (500 μM) under argon with various excitation intensities (λ_{ex} = 650 nm). (d) The dependence of the TTA-UC intensity of the solution of PdTPBP and BDP (compound **7)** on the various incident power density. The results fit the lines that have slopes of 1.89 (black, below) and 1.17 (red, above) in the low- and high-power regions, respectively. I_{th} is 46.7 mW cm^{-2}.

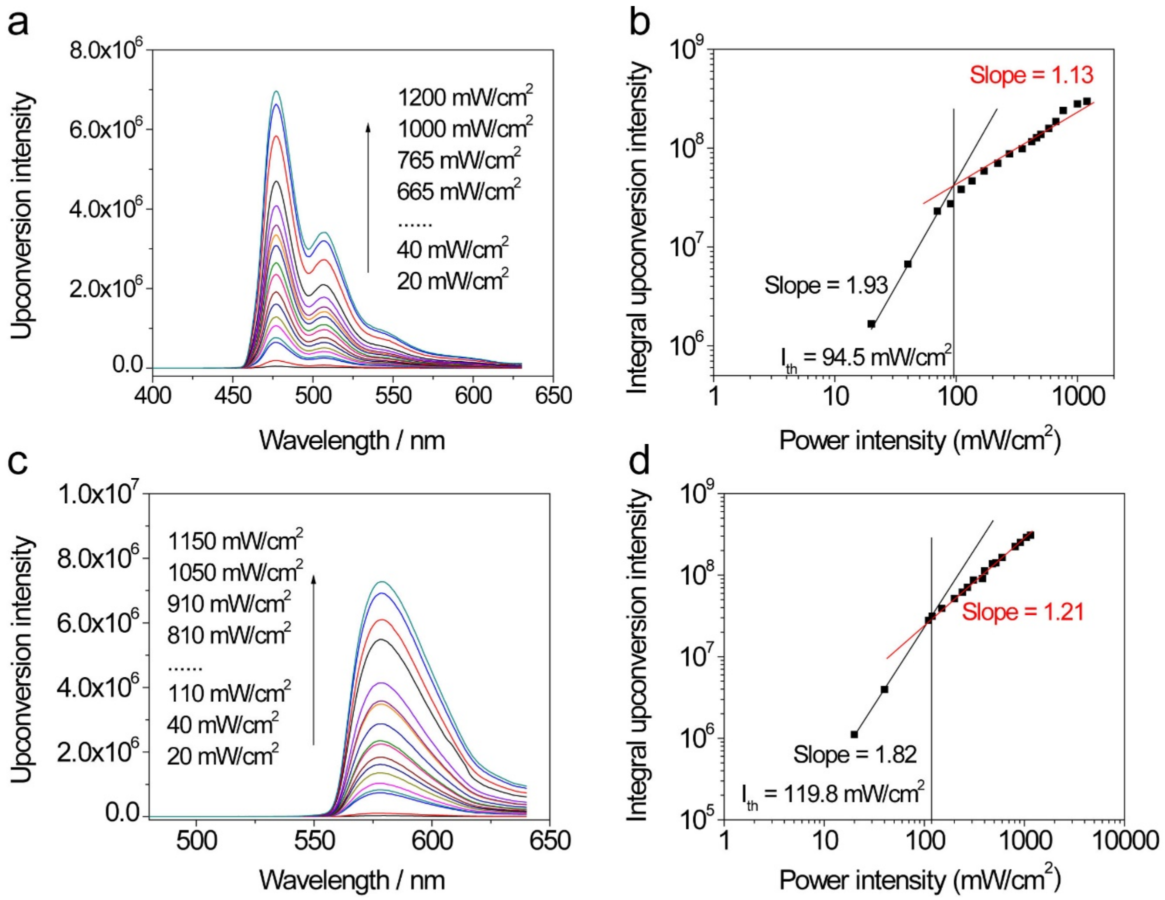

Supplementary Figure 14. (a) Upconversion emission spectra of the TTA-UC system of PtTNP (10 μM) and Py (compound **8**) (10 mM) under argon with various excitation intensities (λ_{ex} = 650 nm). (b) The dependence of the TTA-UC intensity of the solution of PtTNP and Py (compound **8)** on the various incident power density. The results fit the lines that have slopes of 1.93 (black, below) and 1.13 (red, above) in the low- and high-power regions, respectively, I_{th} is 94.5 mW cm^{-2}. (c) Upconversion emission spectra of the TTA-UC system of PtTNP (10 μM) and BDP (compound **7**) (10 mM) under argon with various excitation intensities (λ_{ex} = 650 nm). (d) The dependence of the TTA-UC intensity of the solution of PtTNP and BDP (compound **7)** on the various incident power density. The results fit the lines that have slopes of 1.82 (black, below) and 1.21 (red, above) in the low- and high-power regions, respectively, I_{th} is 119.8 mW cm^{-2}.

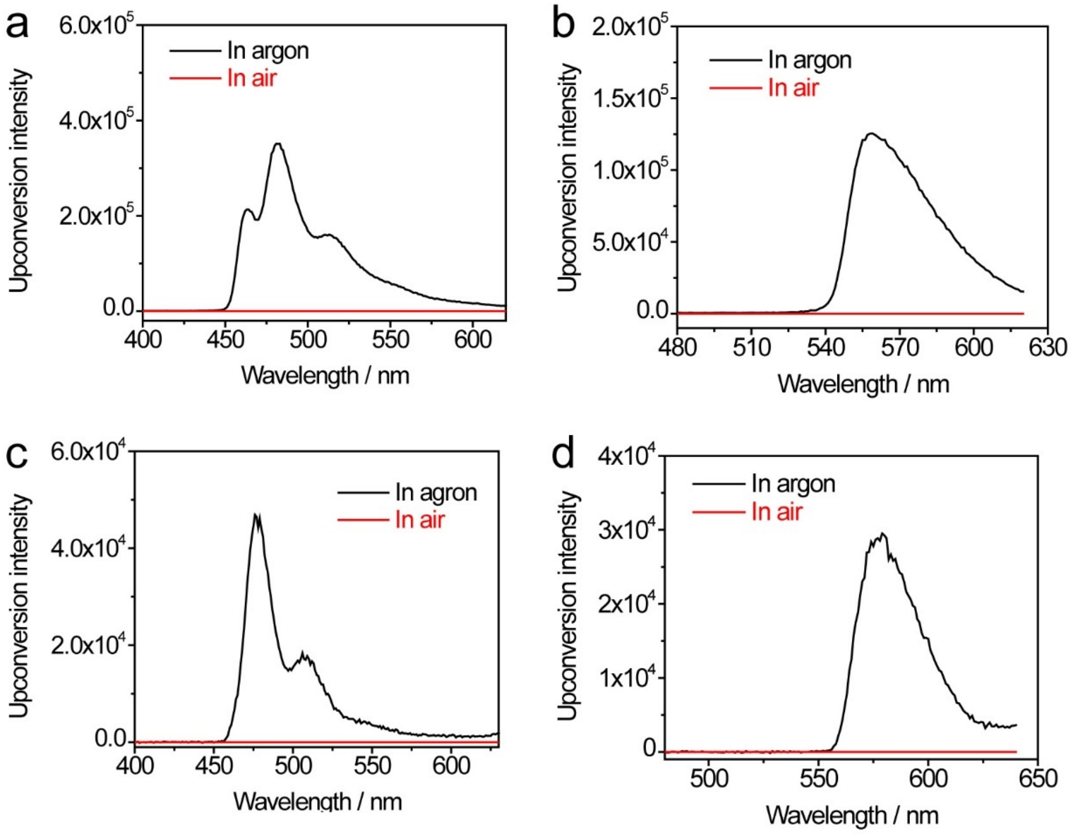

Supplementary Figure 15. Upconversion emission spectra of (a) PdTPBP (10 μM) and Py (compound **8**) (500 μM), (b) PdTPBP (10 μM) and BDP (compound **7**) (500 μM), (c) PtTNP (10 μM) and Py (compound **8**) (10 mM), (d) PtTNP (10 μM) and BDP (compound **7**) (10 mM), λ_{ex} = 650 nm, 20 mW cm^{-2}, in toluene.

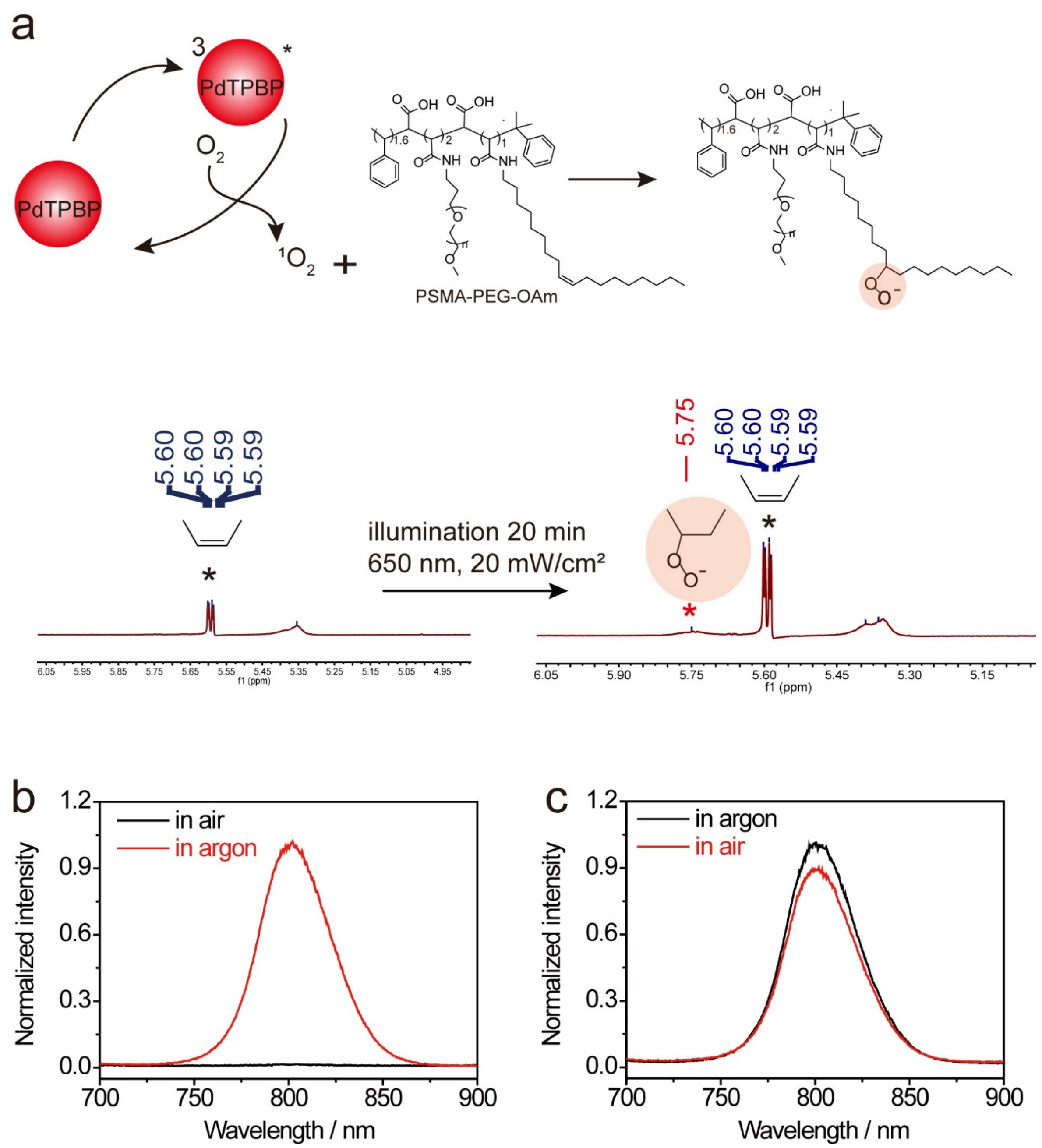

Supplementary Figure 16. (a) Upper: A schematic diagram of the mechanism of PSMA-PEG-OAm scavenge oxygen and molecular structure of PSMA-PEG-OAm. Bottom: ^{1}H NMR monitor PSMA-PEG-OAm reacting with singlet oxygen. Black star is double bond, red star is products after double bond oxidation. (b) Normalized phosphorescence spectra of PdTPBP in toluene, in air and in argon, λ_{ex} = 630 nm. (c) Normalized phosphorescence spectra of PdTPBP NPs in PBS

buffer, in air and in argon, λ_{ex} = 630 nm.For the polymer of PSMA-PEG-OAm, it is noteworthy that the hydrophilic properties of PEG and carboxylic acids of the polymer enhance the biocompatibility of the nanoparticle. The unsaturated alkene sidechains of such amphiphilic polymers act as active decoys to react with and exhaust the oxygen from the external environment. We determined the molecular structure change of PSMA-PEG-OAm under light irradiation by nuclear magnetic resonance (NMR). Along with the illumination, the photosensitizer converts di-oxygen into singlet oxygen, which reacts with unsaturated double bonds, thus generating new peaks at 5.75 ppm (a).. Moreover, since phosphorescence is extremely sensitive to di-oxygen, the phosphorescence of PdTPBP is further used to validate the oxygen scavenger role of PAA-OAm (b). The phosphorescence of PSMA-PEG-OAm encased PdTPBP nanoparticles (PdTPBP NPs) was measured in air and argon (c). The phosphorescence intensity in air was found to be quite similar to that under argon. These results demonstrated that PSMA-PEG-OAm can provide a hypoxia microenvironment to reduce oxygen quenching of the triplet state of PdTPBP.

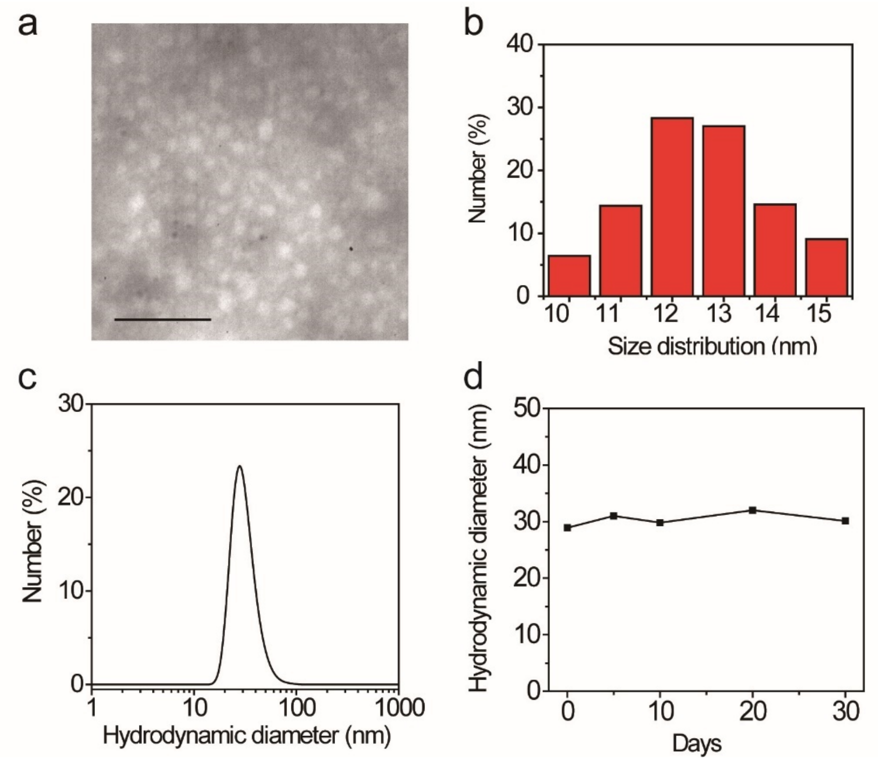

Supplementary Figure 17. (a) TEM imaging of TTAP NPs, scar bar is 100 nm; (b) size distribution of TTAP NPs; (c) hydrodynamic diameter of TTAP NPs in PBS via DLS; (d) colloidal storage stability of TTAP NPs in PBS.

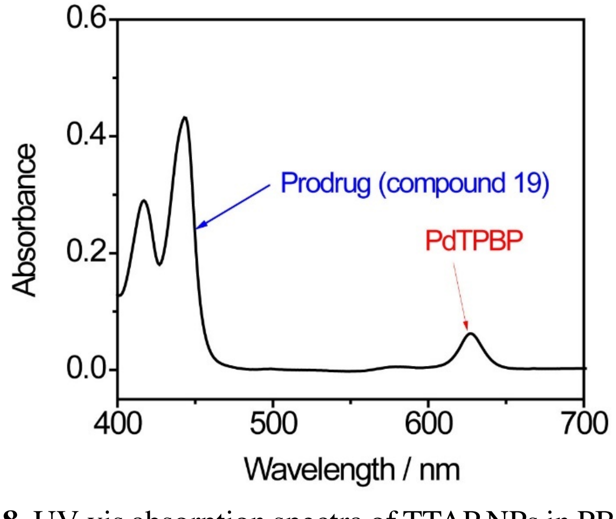

Supplementary Figure 18. UV-vis absorption spectra of TTAP NPs in PBS buffer. The absorption wavelength at 418, 441 nm comes from prodrug (compound **19**), and 627 nm come from PdTPBP. The result suggests TTAP NPs contain both PdTPBP and prodrug.

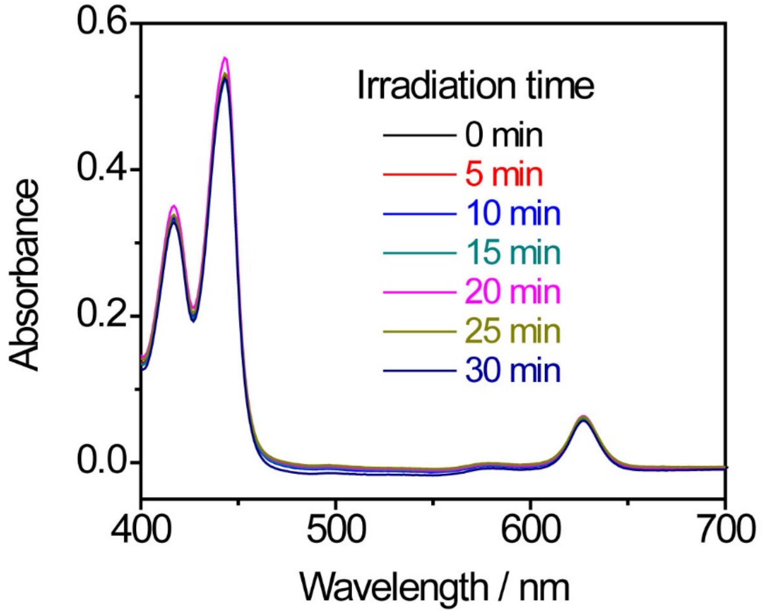

Supplementary Figure 19. UV-vis absorption spectra of TTAP NPs under 650 nm LED (20 mW cm^{-2}) illumination. monitor time (0, 5, 10, 15, 20, 25, 30 min). We did not observe obvious photobleaching, suggesting the TTAP NPs' robust photostability.

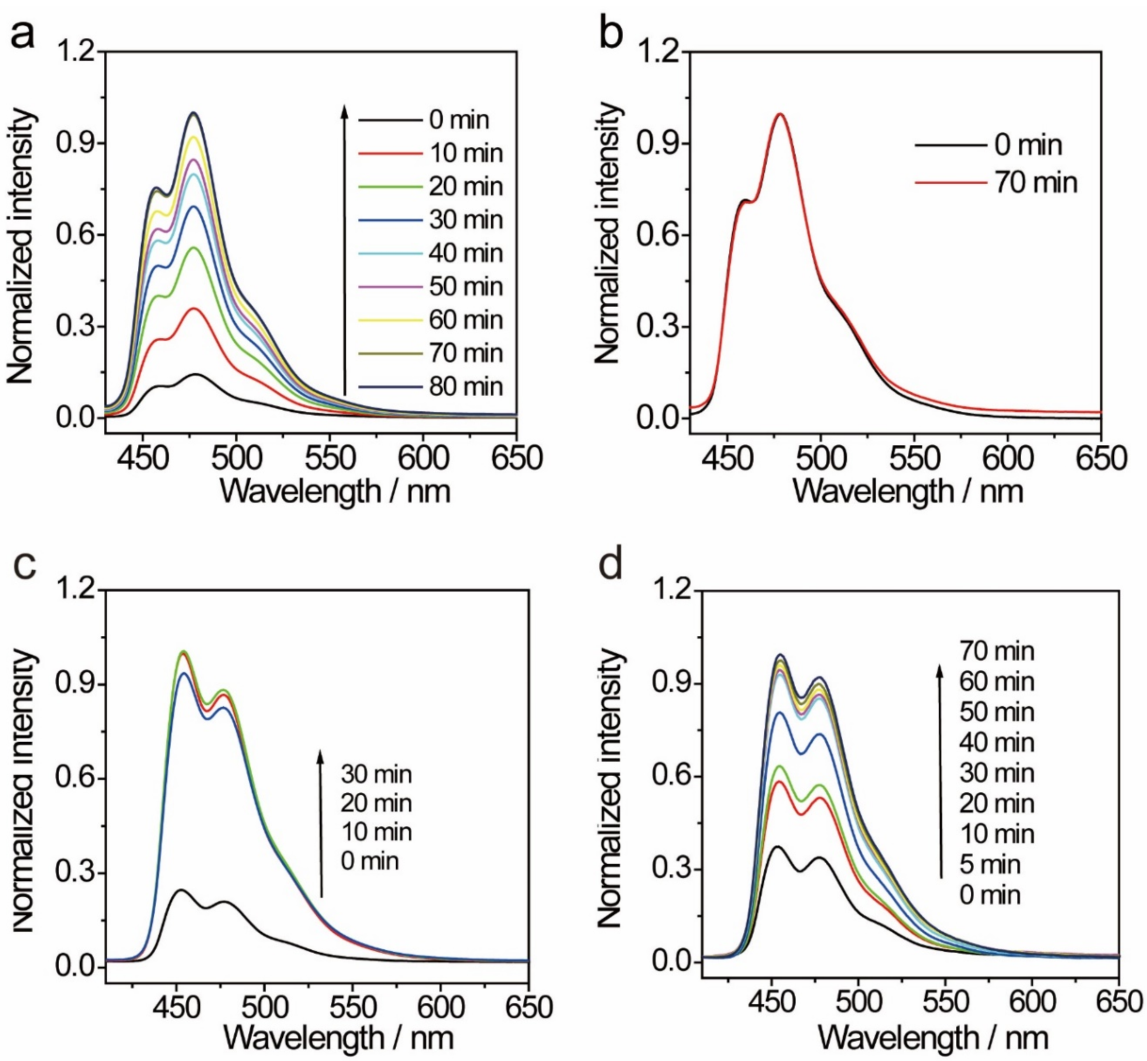

Supplementary Figure 20. Monitoring the photoactivation chlorambucil releasing process of TTAP NPs with the fluorescence spectra of prodrug (compound **19**). (a) Blue light mediated photolytic reaction (455 nm LED, 20 mW cm^{-2}) for Py NPs; (b) Far red light mediated photolytic reaction (650 nm LED, 20 mW cm^{-2}) for Py NPs; (c) Far red light mediated photolytic reaction (650 nm laser, 100 mW cm^{-2}) for TTAP NPs; (d) Far red light mediated photolytic reaction (650 nm LED, 20 mW cm^{-2}) for TTAP NPs.As shown in b, A similar nanoparticle consisting of the prodrug (compound **19**) without the PdTPBP (Py NPs) was prepared as a stringent control. Upon 650 nm illumination of Py NPs for 60 min, no observable uncaged chlorambucil was detected, excluding the possibility of the direct uncaging of chlorambucil by 650 nm light. However, after uncaging via long wavelength light for TTAP NPs, after uncaging, the prodrug (compound **19**) is able to convert into hydrophilic chlorambucil, which can be released from the nanoparticles and kill tumor cells. When we illuminated TTAP NPs with a 650 nm LED(20 mW cm^{-2}), we found

that the prodrug was uncaged, resulting in greater than 48% activation of the prodrug within 30 min, and a maximum photo-release of approximately 64% of the prodrug after 60 min (c-d, and supplementary Figure 21a).

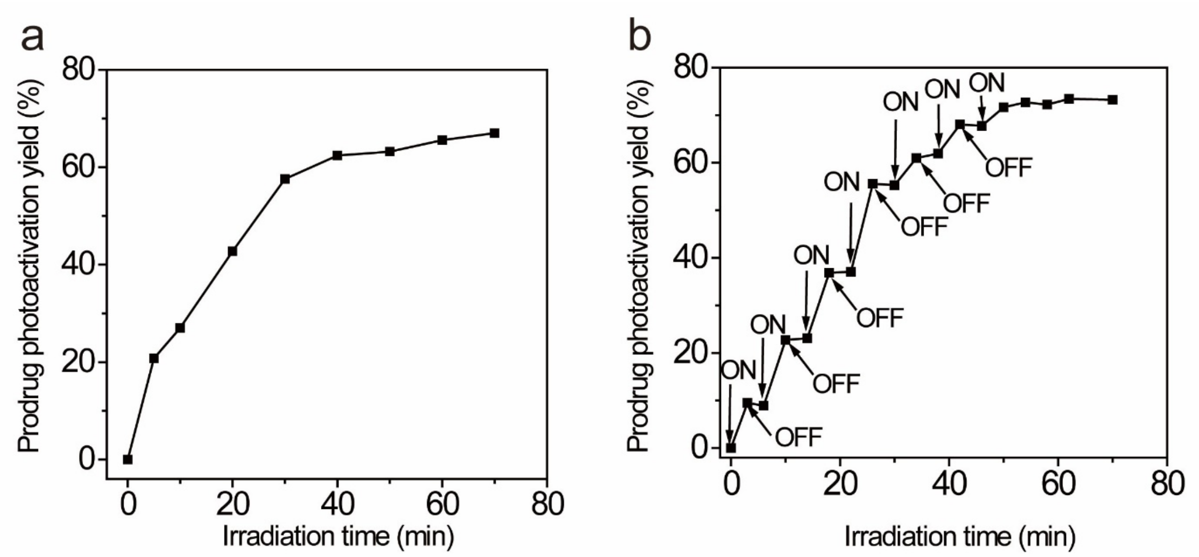

Supplementary Figure 21. (a) The release profiles of chlorambucil from TTAP NPs in PBS buffer with 650 nm LED irradiation, 20 mW cm^{-2}. (b) The photolytic process for prodrug (compound **19**) from TTAP NPs with 650 nm LED irradiation. “ON” and “OFF” indicate the initiation and termination of LED irradiation, respectively; the LED (650 nm, 20 mW cm^{-2}).

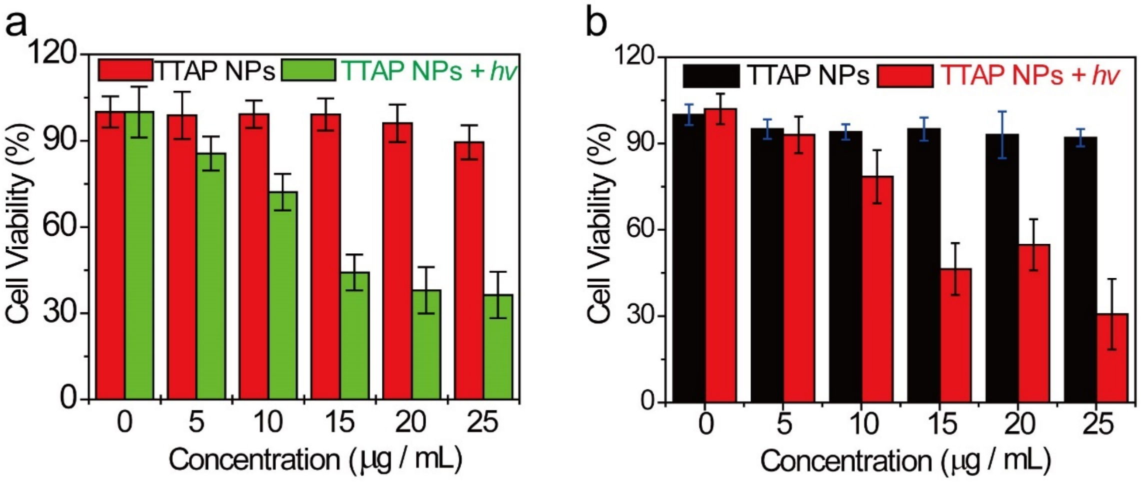

Supplementary Figure 22. (a) MTT assay of Hela cells viability of different concentrations of TTAP NPs and TTAP NPs + *hv*. (b) 4T1 cell viability in different concentrations of TTAP NPs with and without light irradiation. The value for IC_{50} with respect to the Hela and 4T1 cells in the *in vitro* experiment: 17.3 ± 3.1 mg mL^{-1} (Hela cells) and 20.6 ± 2.6 mg mL^{-1} (4T1).

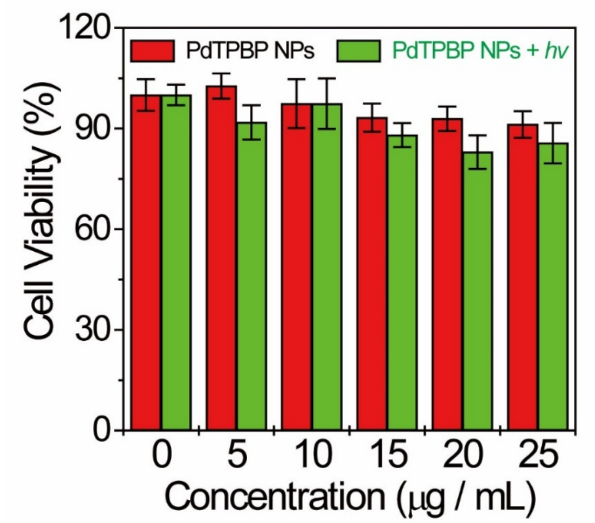

Supplementary Figure 23. Hela cell viability in different concentrations of PdTPBP NPs with and without light irradiation.

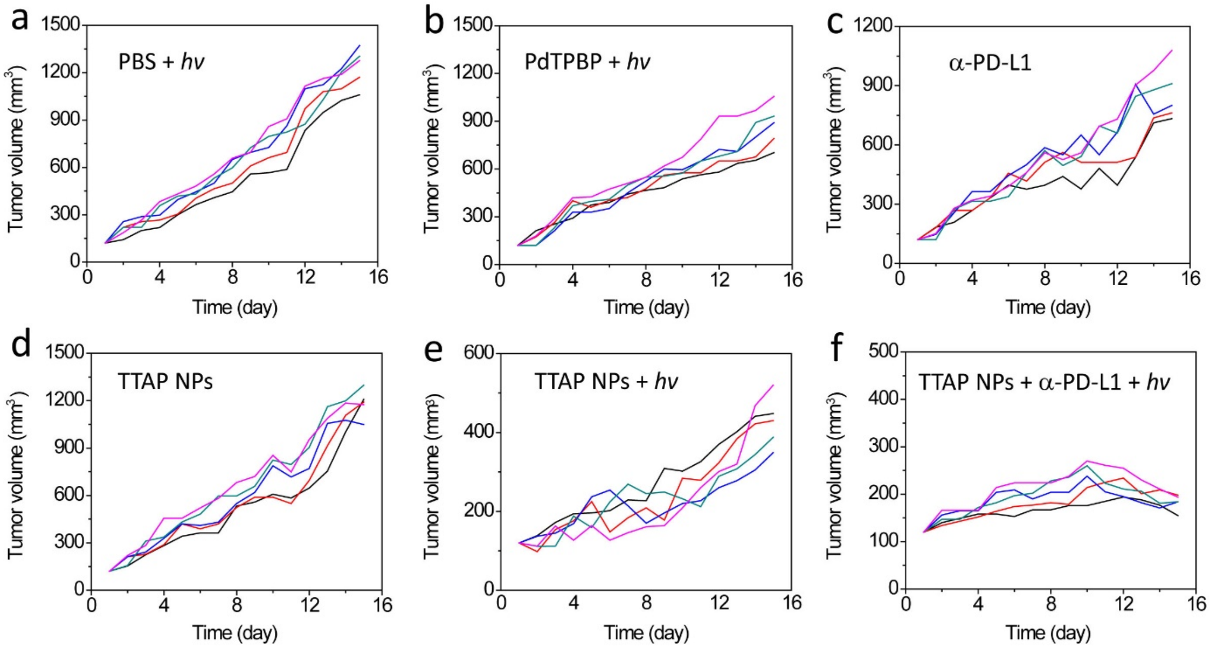

Supplementary Figure 24. Synergistic TTAP NPs and α-PD-L1 for inhibition of 4T1 tumor growth *in vivo*. (a-f) individual tumor growth kinetics in control and treated groups. In group 1 (PBS + *hv*), we did not observe tumor growth inhibition; after 15 days, the tumor volume increased 7.5-fold. This result suggested that low power intensity far red light did not have obvious photothermal effects in regard to cancer therapy. In group 2 (PdTPBP NPs + *hv*), the tumor volume also increased 6.5-fold and we did not observe significant tumor growth inhibition by PdTPBP NPs. This is likely due to the presence of an unsaturated bond on these nanoparticles that can exhaust singlet oxygen to reduce the possible photodynamic effect. In group 4 (TTAP NPs only), the tumor volume also increased 7.0-fold, suggesting a low inherent toxicity of TTAP NPs in the dark. However, in group 5 (TTAP NPs + *hv*), the tumor volume only increased 3.9-fold. These results demonstrated that the anticancer drug was efficiently photolyzed and released for tumor cell killing. In group 3, the injection of α-PD-L1 also led to tumor growth inhibition; a result that is consistent with previously reported results. In contrast, in group 6, the tumor showed a slow

growth rate from days 1 to 6, but post α-PD-L1 injection at 7th, 8th and 9th day, the tumor volume was remarkably suppressed. More significantly, we also measured the left tumor growth rates. In groups 1, 2, and 4, we did not observe tumor inhibition. In group 3, the tumor presented a slower tumor growth rate than seen in group 1, as α-PD-L1 can mitigate the metastasis. However, compared to group 5, group 6 presented significant tumor suppression (Figure 3b). We also measured tumor weight after 15 days' treatments. In group 6, both the right and left tumor showed obvious reduction (Figure 3d). These experimental results clearly demonstrated that the TTAP mediated prodrug photolytic system improves the checkpoint blockade immunotherapy efficacy and promotes abscopal effects.

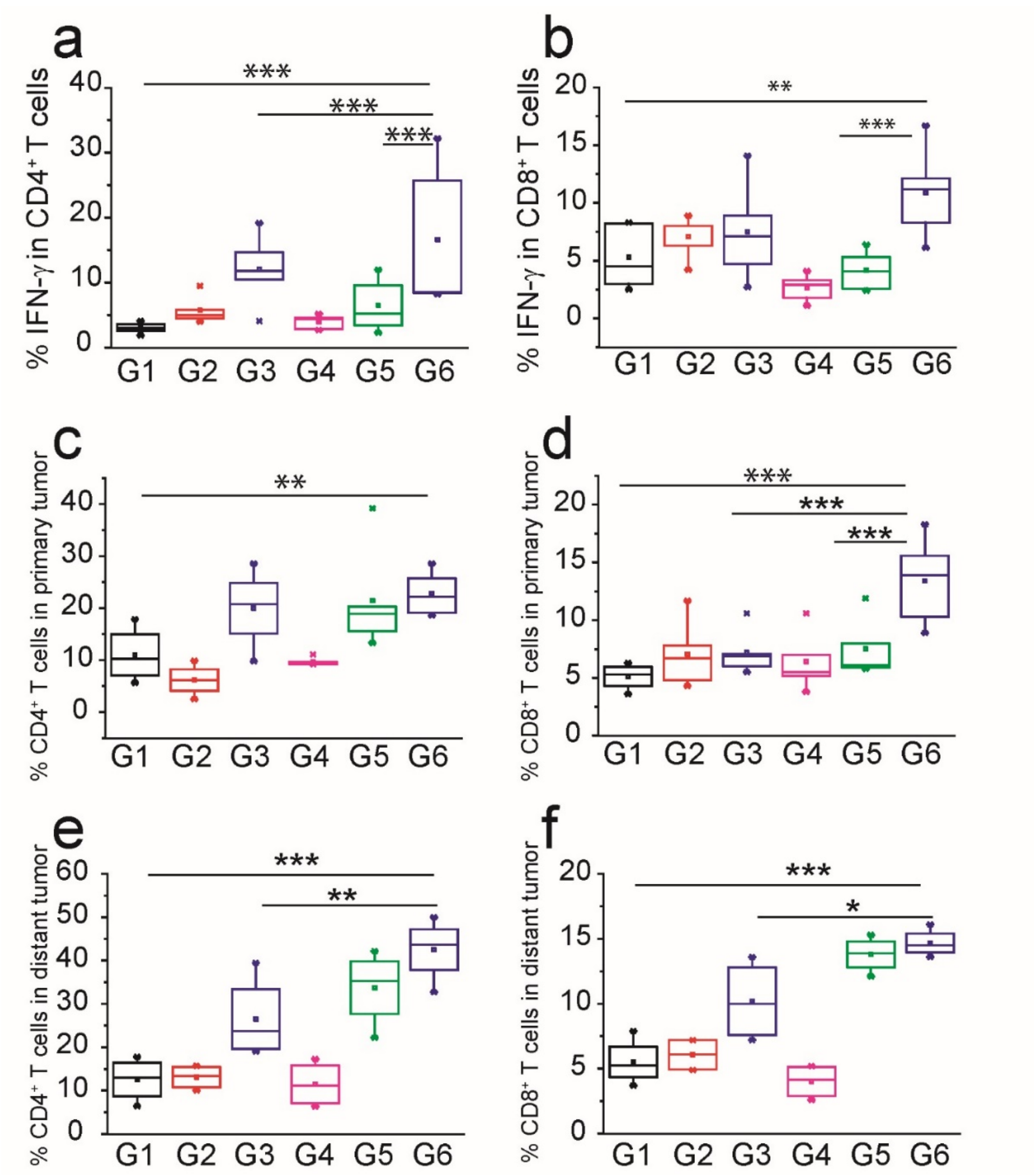

Supplementary Figure 25. Tumor-specific immune responses. (a-b) Splenocytes were stimulated for 4 h with PMA/ionomycin, the frequency of IFN-γ– producing $CD4^+$ and $CD8^+$ T cells was measured by flow cytometry. (c-d) The percentage of tumor-infiltrating (c) $CD4^+$ T cells, (d) $CD8^+$ T cells in the primary tumor. (e-f) The percentage of tumor-infiltrating (e) $CD4^+$ T cells, (f) $CD8^+$ T cells in the distant tumor. Data are expressed as means ± s.d. (n = 5). $^{*}p < 0.05$, $^{**}p < 0.01$, and $^{***}p < 0.001$.

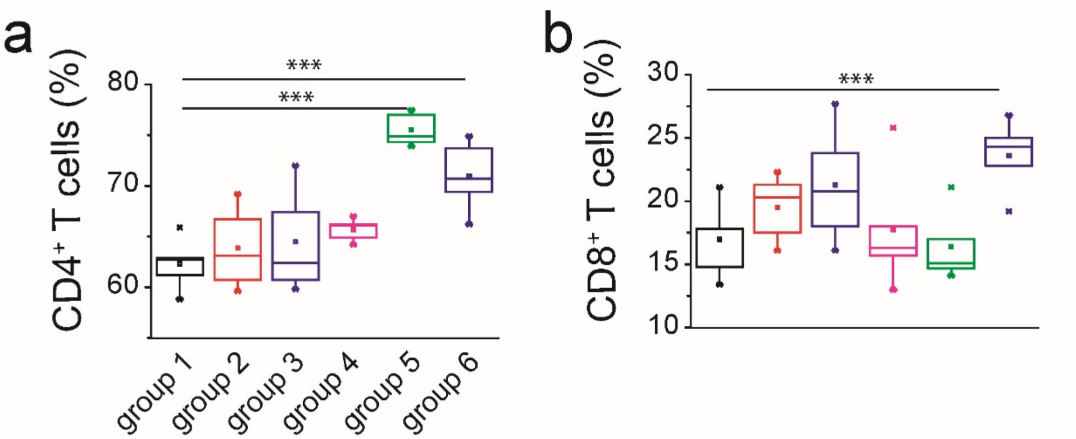

Supplementary Figure 26. The percentage of tumor-infiltrating (a) CD4+ T cells or (b) CD8+ T cells in the spleen. Data are expressed as means ± s.d. (n = 5). $^{*}P < 0.05$, $^{**}P < 0.01$, and $^{***}P < 0.001$.

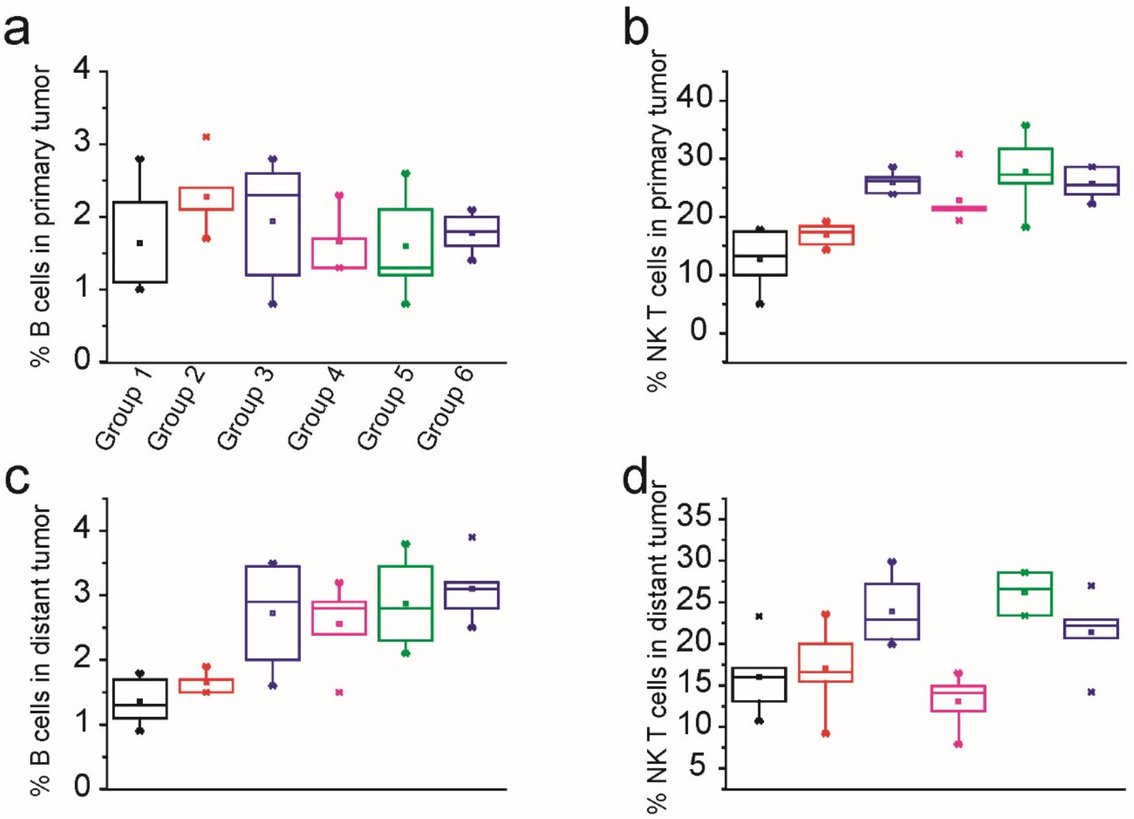

Supplementary Figure 27. The percentage of tumor-infiltrating (a) B cells, (b) NK T cells in the primary tumor, and (c) B cells, (d) NK T cells in the distant tumor. Data are expressed as means ± s.d. (n = 5).

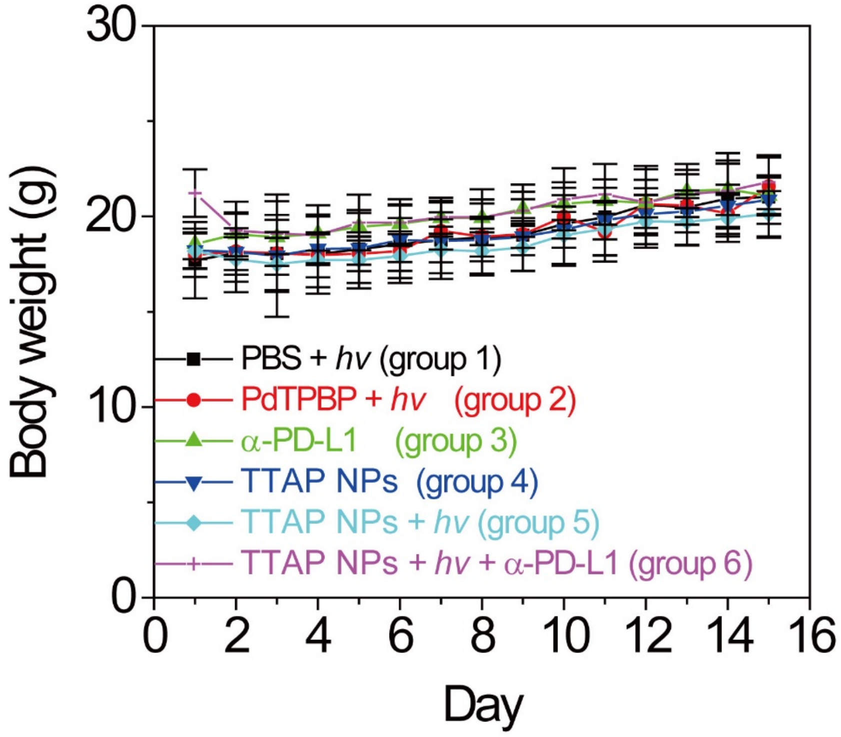

Supplementary Figure 28. The mice body weight change of anti-tumor immunotherapy model.

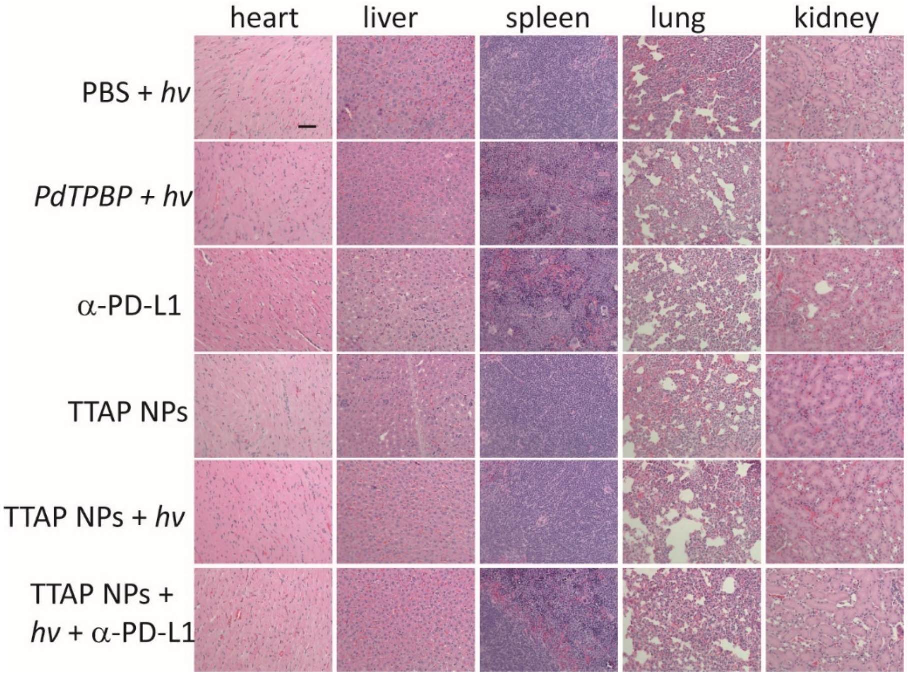

Supplementary Figure 29. H&E staining images of major organs (heart, liver, spleen, lung and kidney) of 4T1 tumor bearing mice after injection of TTAP NPs for 14 days, scale bar represents 100 μm.

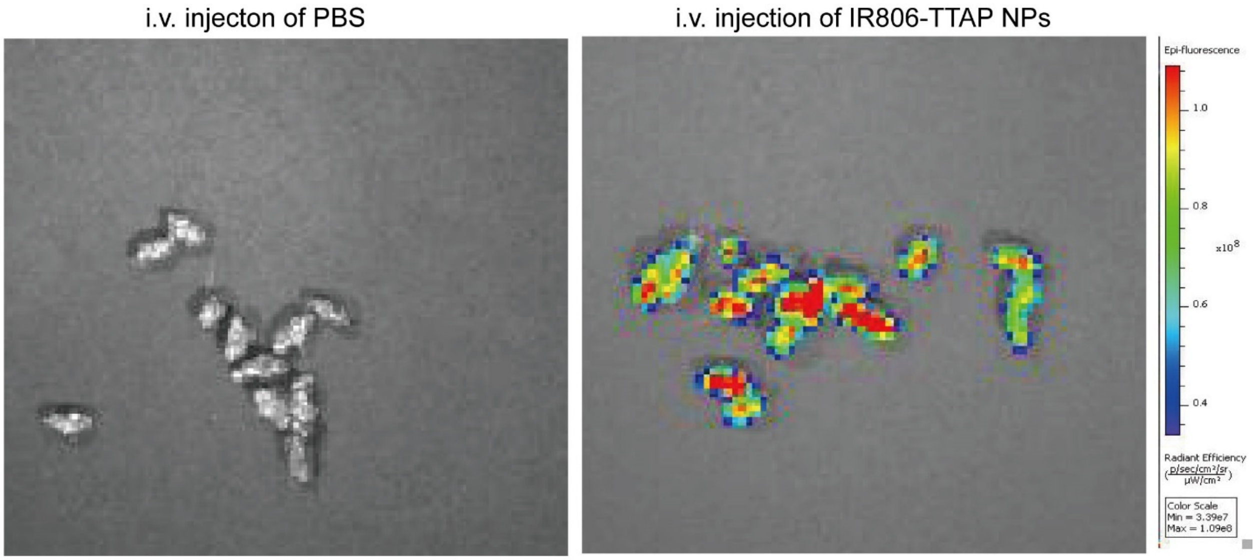

Supplementary Figure 30. IVS imaging of the mice feces for i.v. injection of PBS (control) and i.v. injection of IR806-TTAP NPs.

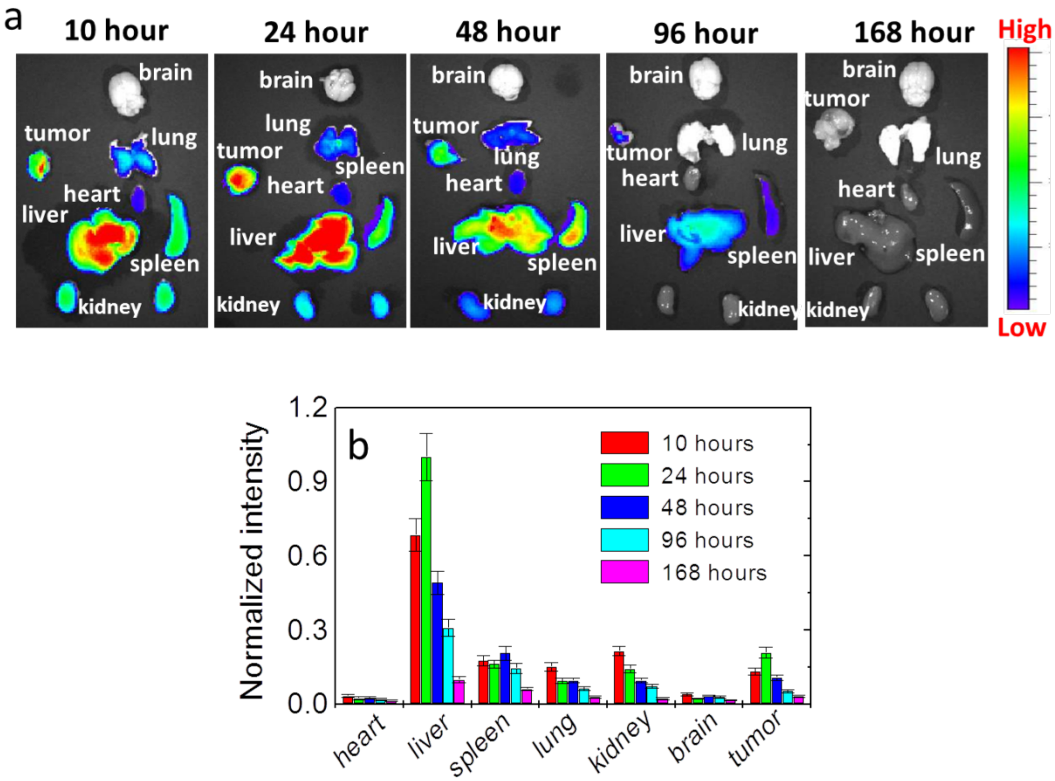

Supplementary Figure 31. *In vivo* distributions and biosafety of IR806-RGD-TTAP-NPs. (a)

Time dependent *ex vivo* NIR fluorescence in major mice organs (heart, liver, spleen, lung, kidney, tumor) including 10, 24, 48, 96 and 168 hours. (b) Normalized fluorescence intensity in mice organs, Values are the means ± s.e.m. (n = 3 mice per group). We can see that ten hours after treatment with the IR806-RGD-TTAP NPs by intravenous injection, the tumor and organs, including the brain, heart, liver, spleen, lung, and kidney were excised from tumor-bearing mice and imaged *ex vivo*. An intense NIR fluorescence was seen in the excised tumor, which indicates efficient IR806-RGD-TTAP NPs accumulation in the tumor. After 48 h, the fluorescence from the liver and the tumor had reduced. By 96 h, the fluorescence of the liver and the tumor had significantly weakened. We quantified the fluorescence intensity of the tumor and each organ from the IR806-RGD-TTAP NPs treated group at different time-points. The fluorescence intensity of the tumor was higher than that in organs at 10 h, except in the liver. However, after 48 h, the fluorescence from the liver was reduced by 50%, but that from the tumor only reduced by 10%. At 96 h, the fluorescence from the liver could hardly be observed. A possible reason for this result might be that IR806-RGD-TTAP NPs is mainly eliminated by macrophage cells in the liver. Meanwhile, the fluorescence was observed at kidney at 10, 24, 48 hours and disappeared at 96 and 168 hours, suggesting the RGD-TTAP NPs may be excluded from the body through kidney.

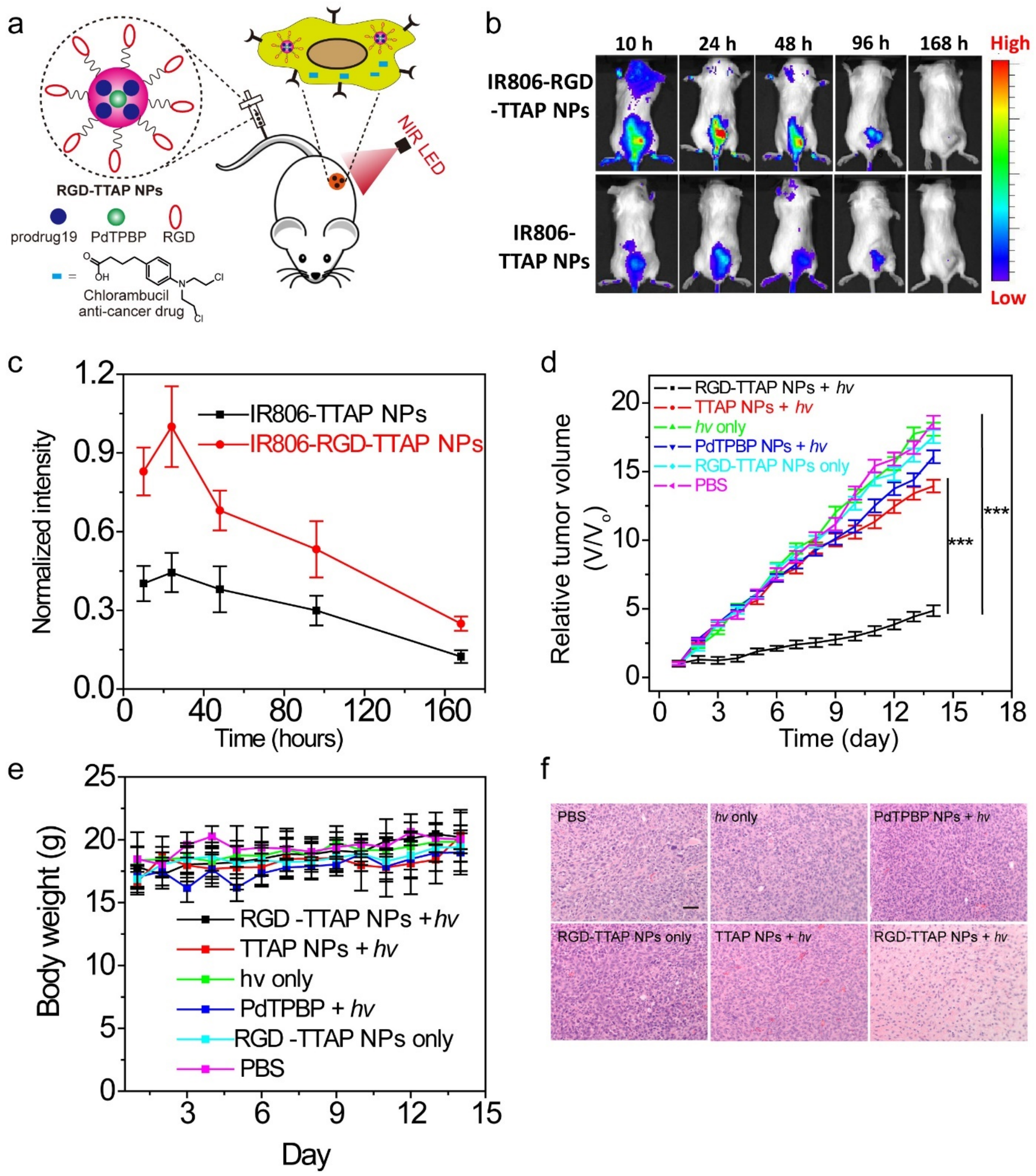

Supplementary Figure 32. (a) Schematic illustration of TTAP NPs mediated targeted tumor therapy. (b) Specific-targeted IR806 fluorescence tumor imaging *in vivo*. (c) Normalized fluorescence intensity of IR806-RGD-TTAP NPs and IR806-TTAP NPs in 4T1 tumors at 10, 24, 48 and 96 h. Data are means ± s.e.m. (n = 3 mice). (d) Tumor growth inhibition by TTAP NPs

mediated photoactivatable prodrug release in 4T1 tumors; light irradiation was performed 24 h after injection of TTAP NPs. Values are means ± s.e.m. (n = 5 mice per group). (e) The mice body weight change of targeted tumor therapy. (d) H&E staining of tumor tissue sections from different treatment groups 10 days after treatment; scale bar represents 50 μm. No tumor growth inhibition or tumor tissue necrosis was observed in the group of mice with LED irradiation only (*hv* only), indicating that irradiation with such low power intensity red light had little photothermal effect. No tumor inhibition was observed for the group with injection of only RGD-TTAP NPs, which showed that RGD-TTAP NPs has negligible dark toxicity. Group TTAP NPs plus light illumination also did not display an obvious therapeutic effect, suggesting that cRGD was important for targeting tumor tissue. PdTPBP NPs plus light illumination also did not show obvious tumor inhibition effects, which suggests that PdTPBP has less photo-toxicity. In contrast, the tumor growth in the treatment RGD-TTAP NPs plus light illumination was remarkably suppressed. H&E staining analysis of the tumor tissue also showed clear necrosis, which indicates that RGD-TTAP NPs can be effectively activated by red LED irradiation for intense anticancer effect.

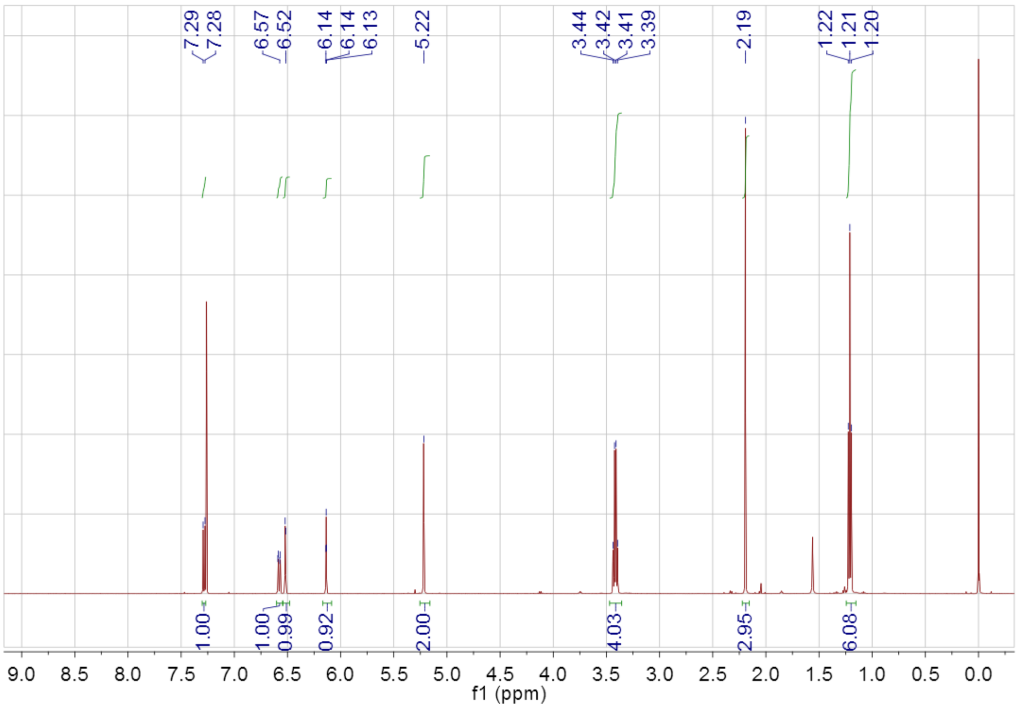

Supplementary Figure 33. ^{1}H NMR of **1** (500 MHz, $CDCl_3$).

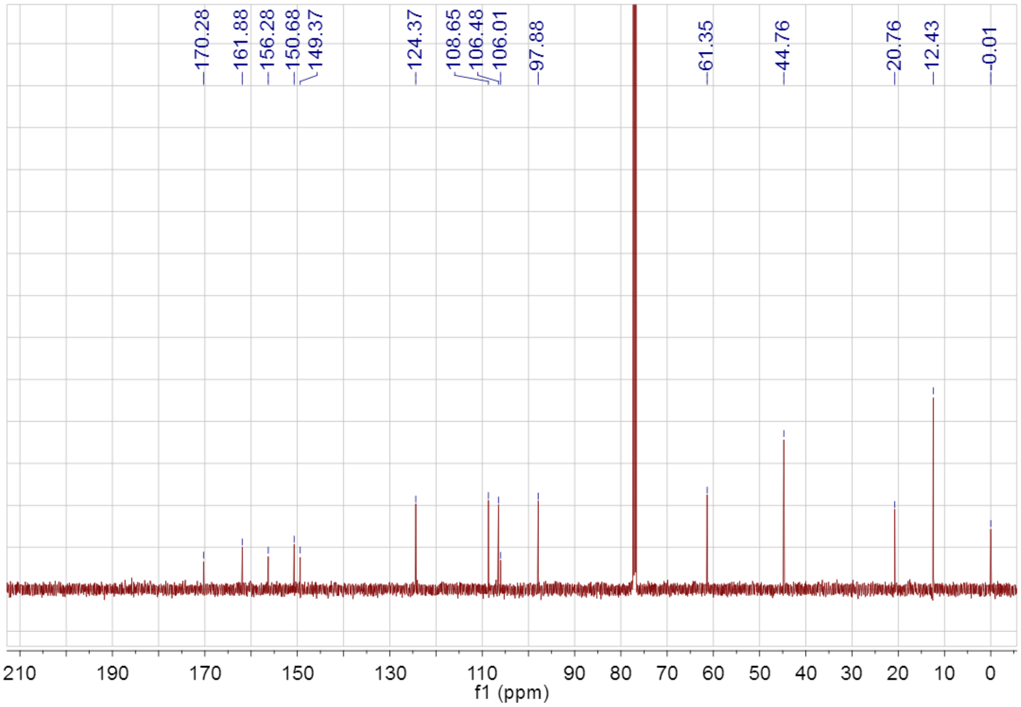

Supplementary Figure 34. ^{13}C NMR of **1** (125 MHz, $CDCl_3$).

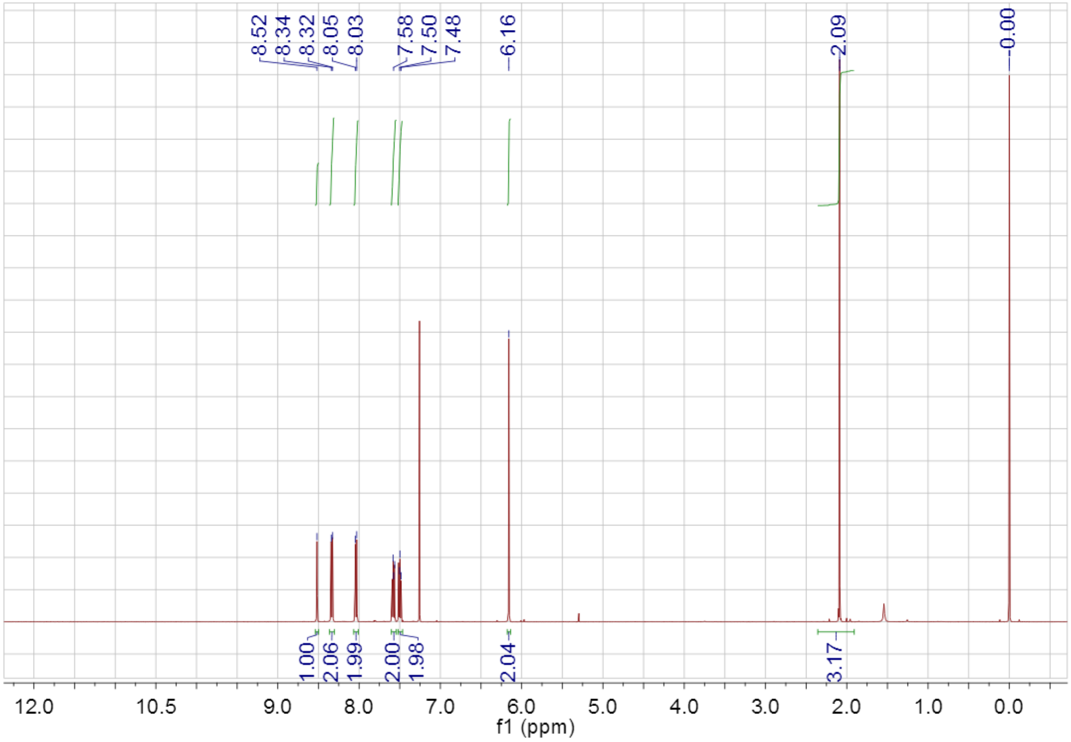

Supplementary Figure 35. ^{1}H NMR of **2** (500 MHz, $CDCl_3$).

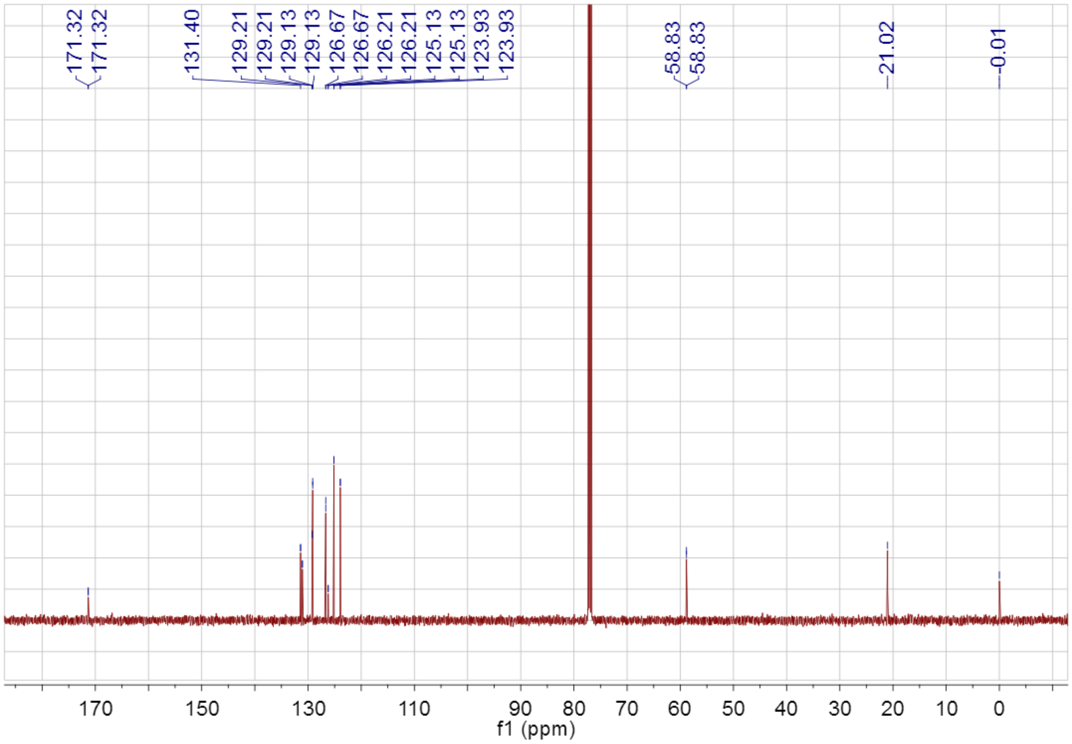

Supplementary Figure 36. ^{13}C NMR of **2** (125 MHz, $CDCl_3$).

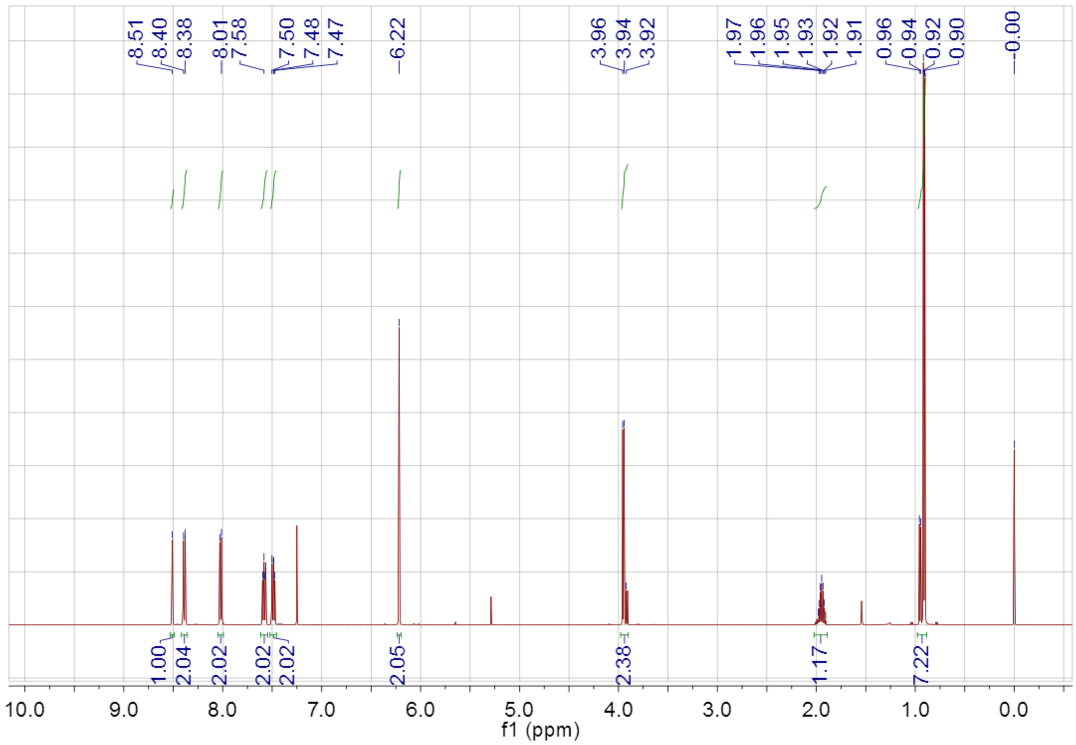

Supplementary Figure 37. ^{1}H NMR of **3** (500 MHz, $CDCl_3$).

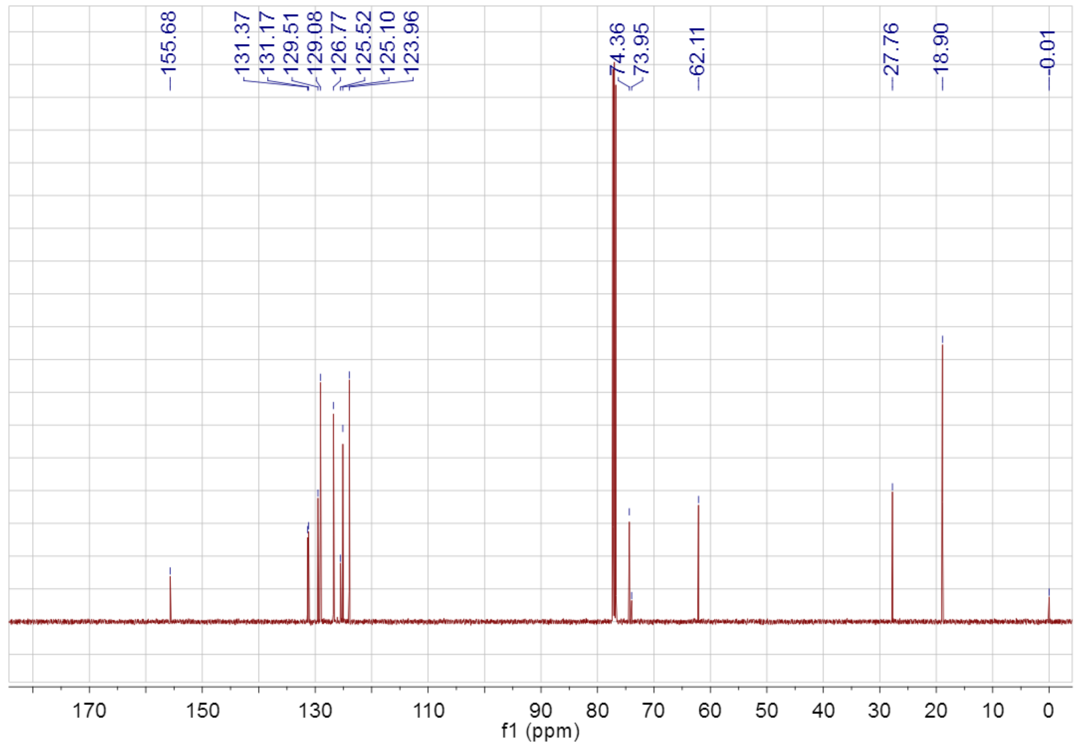

Supplementary Figure 38. ^{13}C NMR of **3** (125 MHz, $CDCl_3$).

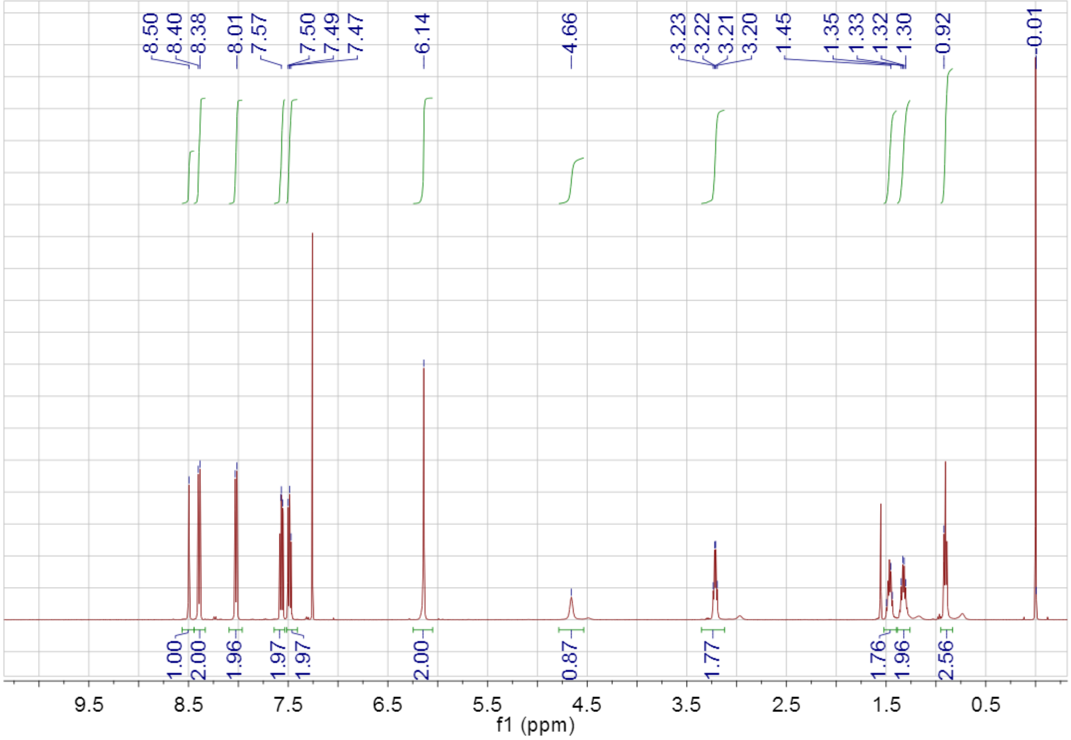

Supplementary Figure 39. ^{1}H NMR of **4** (500 MHz, $CDCl_3$).

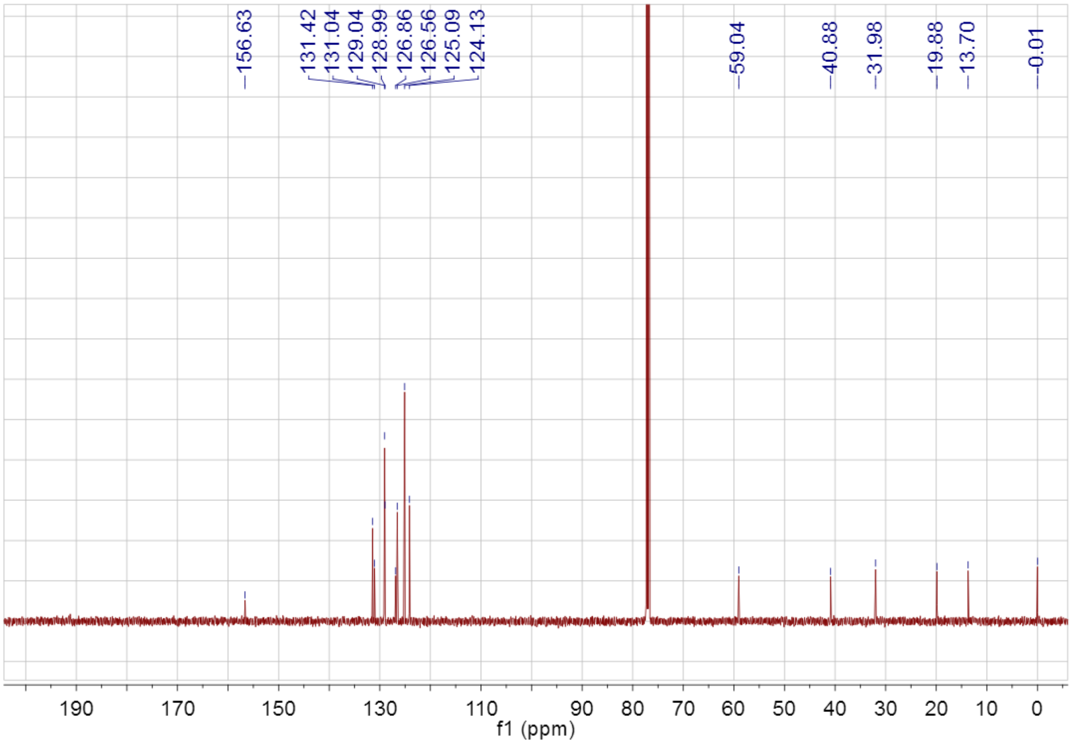

Supplementary Figure 40. ^{13}C NMR of **4** (125 MHz, $CDCl_3$).

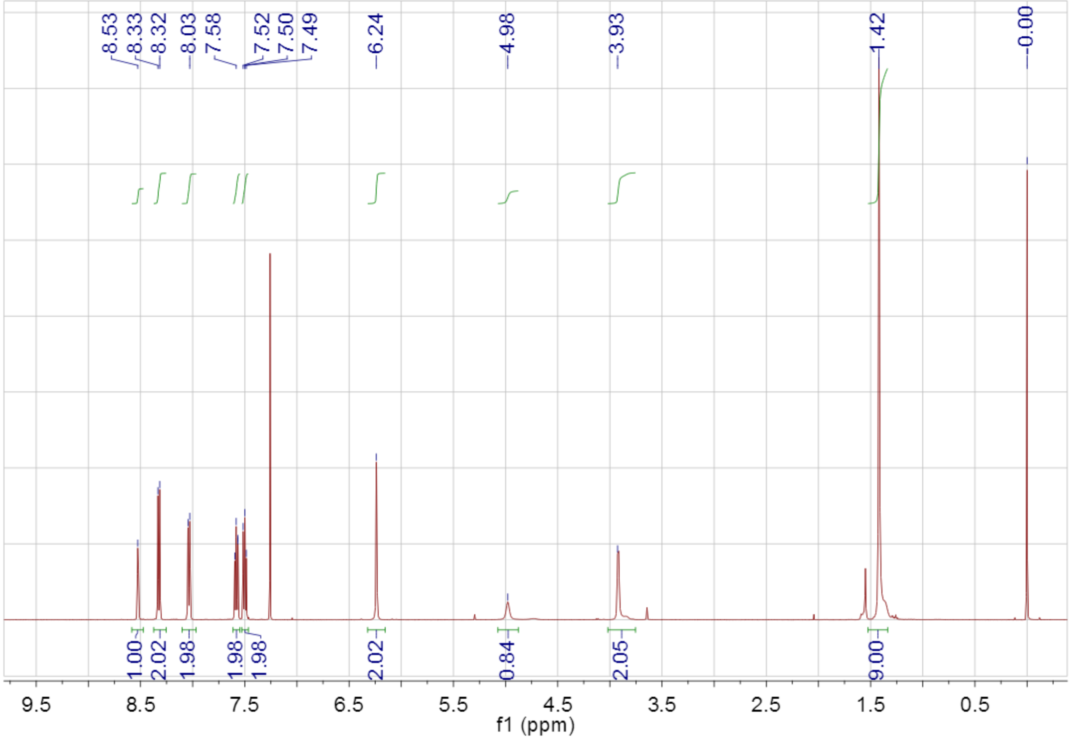

Supplementary Figure 41. ^{1}H NMR of **5** (500 MHz, $CDCl_3$).

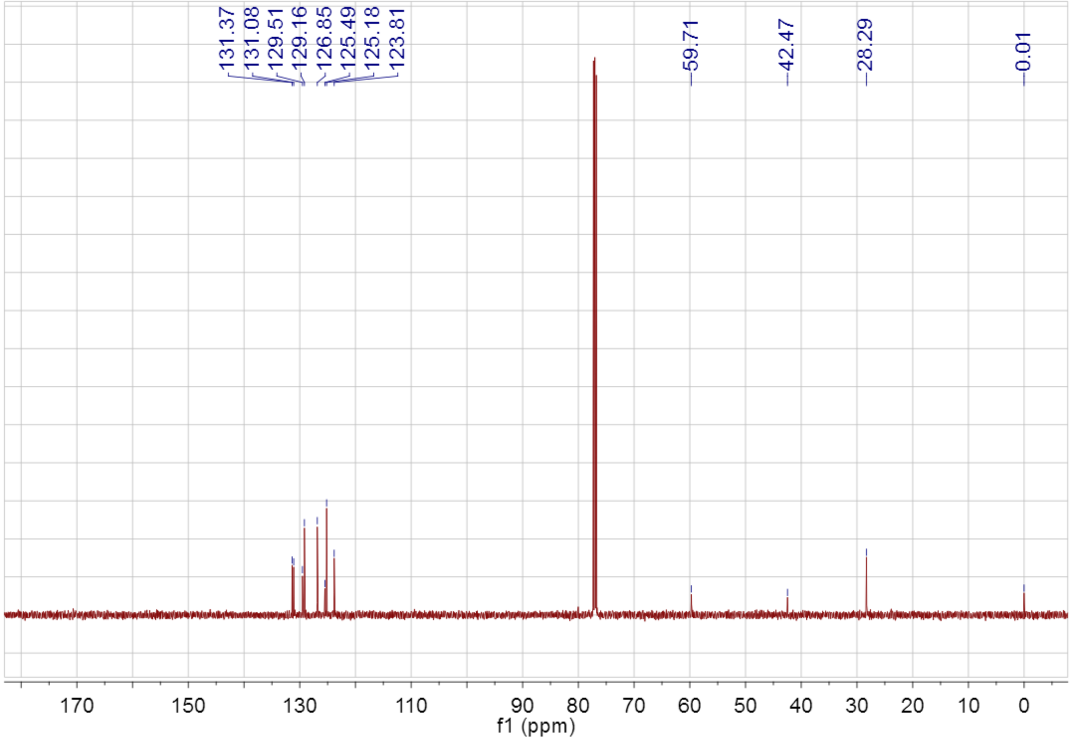

Supplementary Figure 42. ^{13}C NMR of **5** (125 MHz, $CDCl_3$).

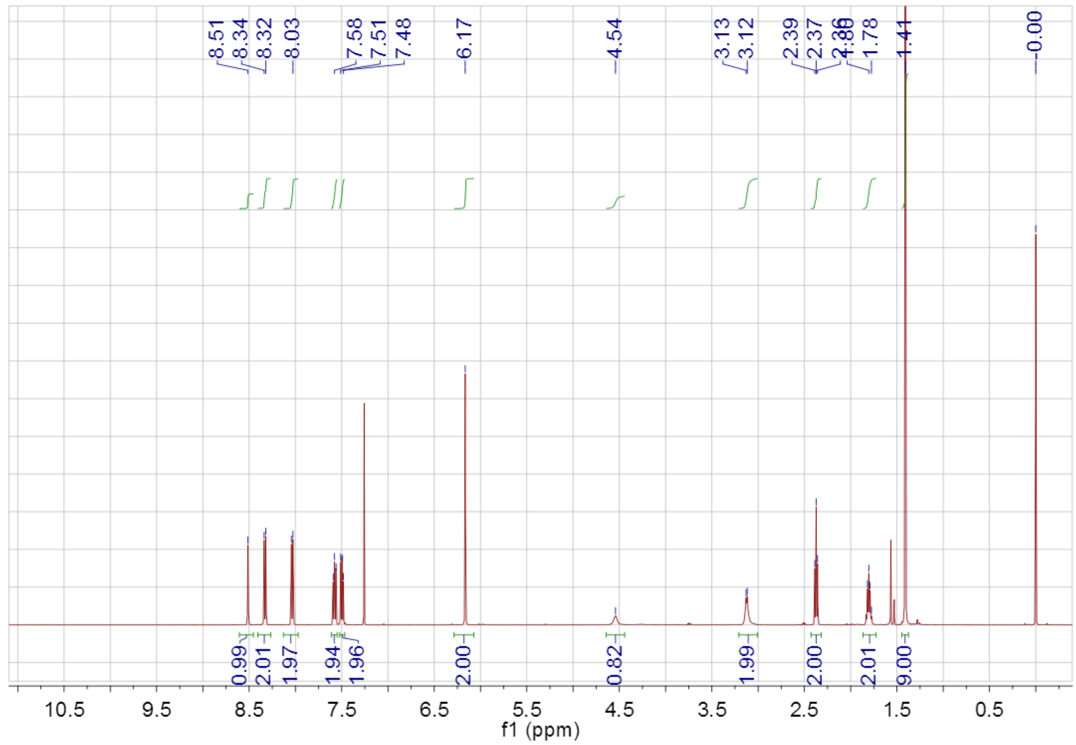

Supplementary Figure 43. ^{1}H NMR of **6** (500 MHz, $CDCl_3$).

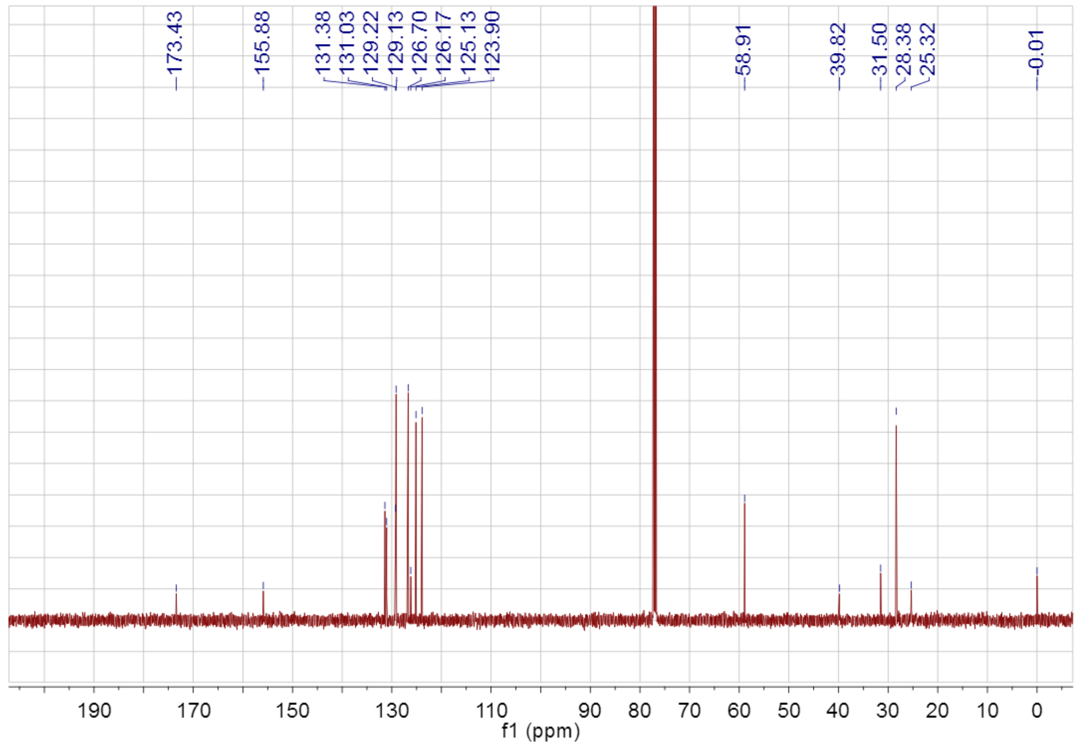

Supplementary Figure 44. ^{13}C NMR of **6** (125 MHz, $CDCl_3$).

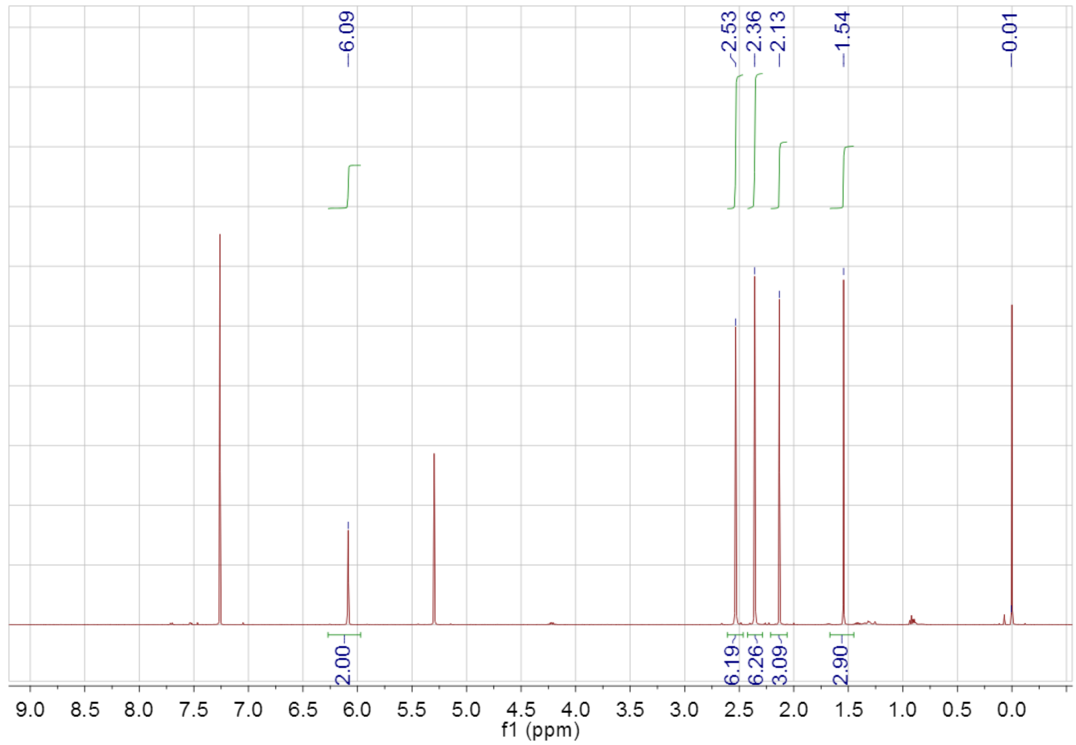

Supplementary Figure 45. ^{1}H NMR of **7** (500 MHz, $CDCl_3$).

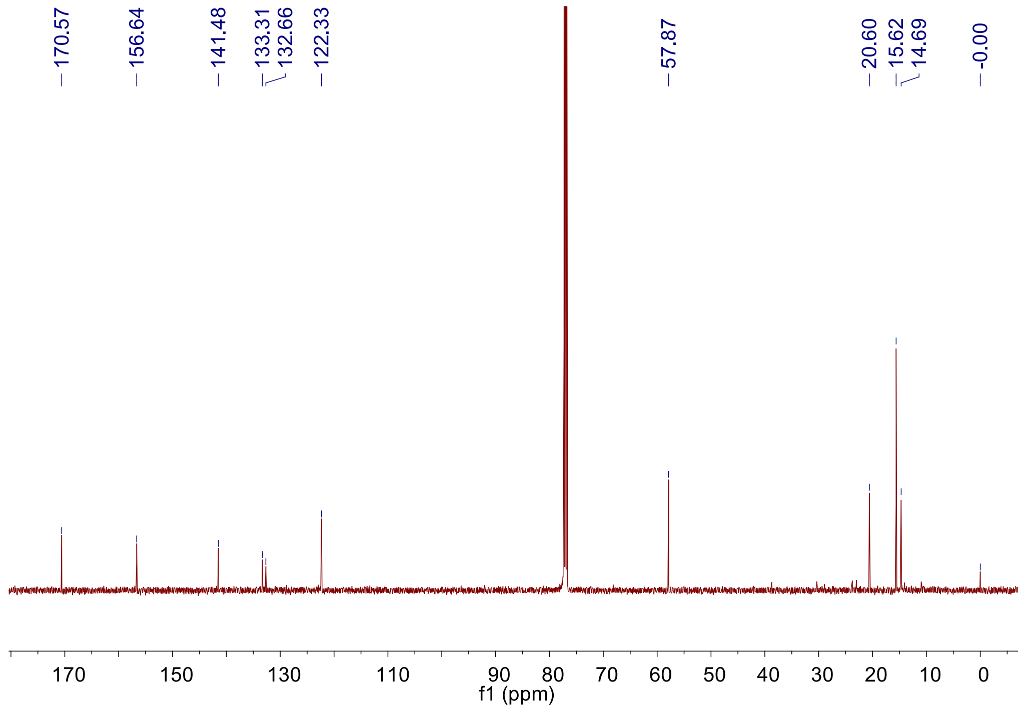

Supplementary Figure 46. ^{13}C NMR of **7** (125 MHz, $CDCl_3$).

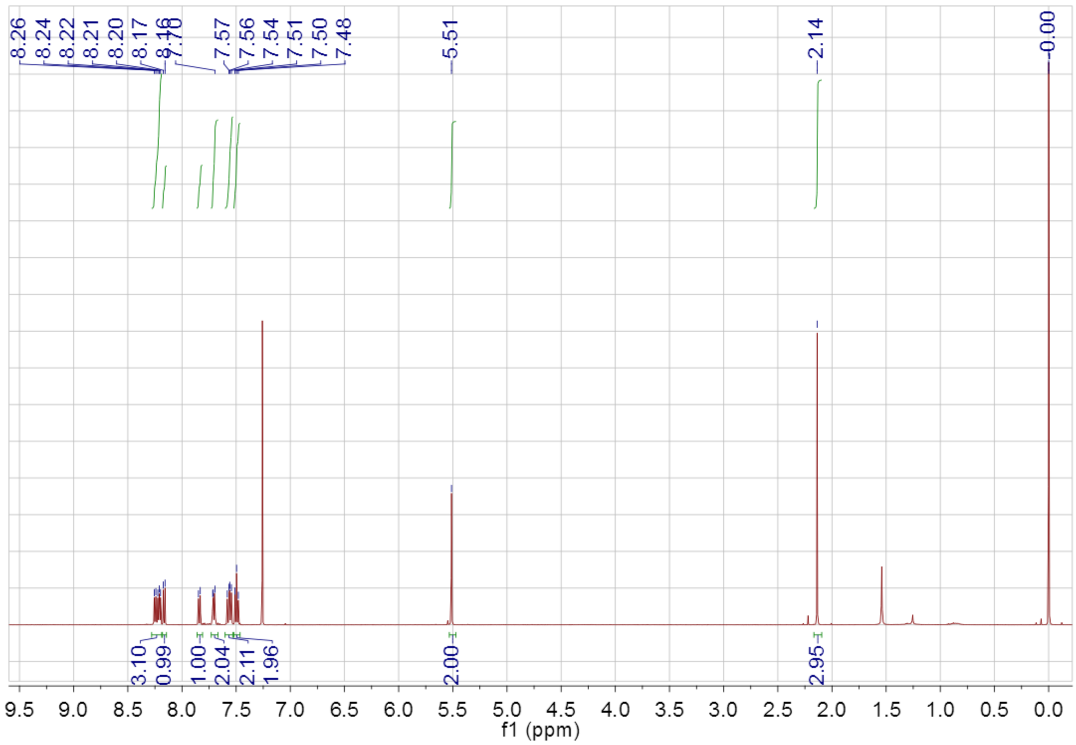

Supplementary Figure 47. ^{1}H NMR of **8** (500 MHz, $CDCl_3$).

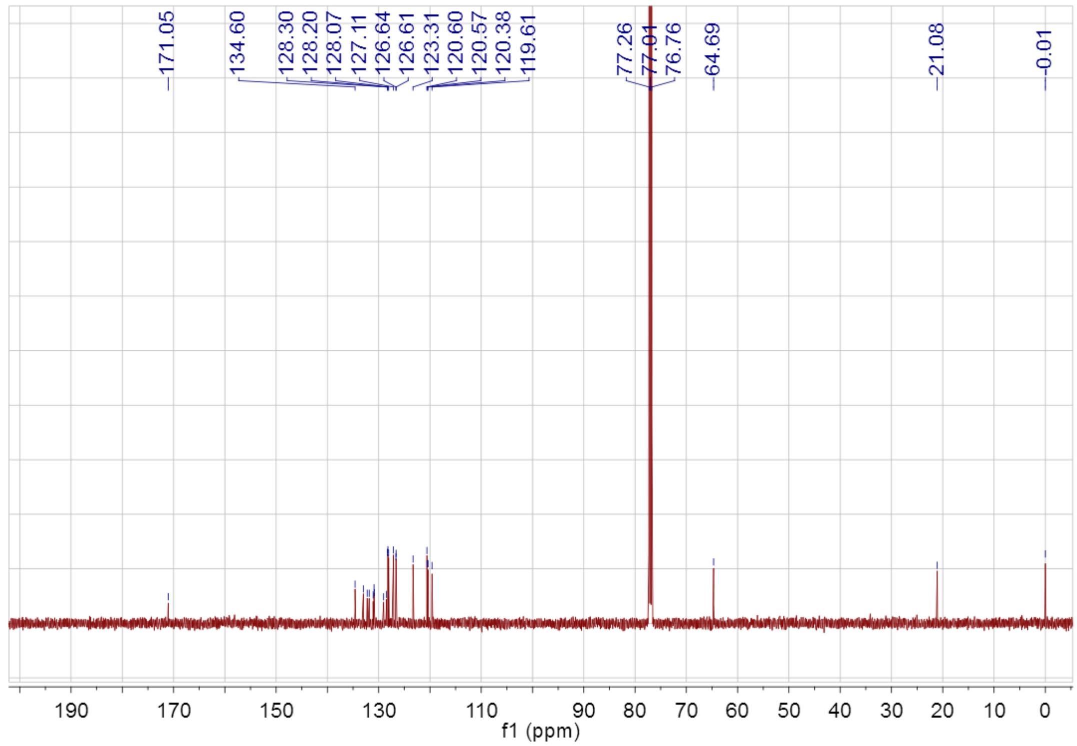

Supplementary Figure 48. ^{13}C NMR of **8** (125 MHz, $CDCl_3$).

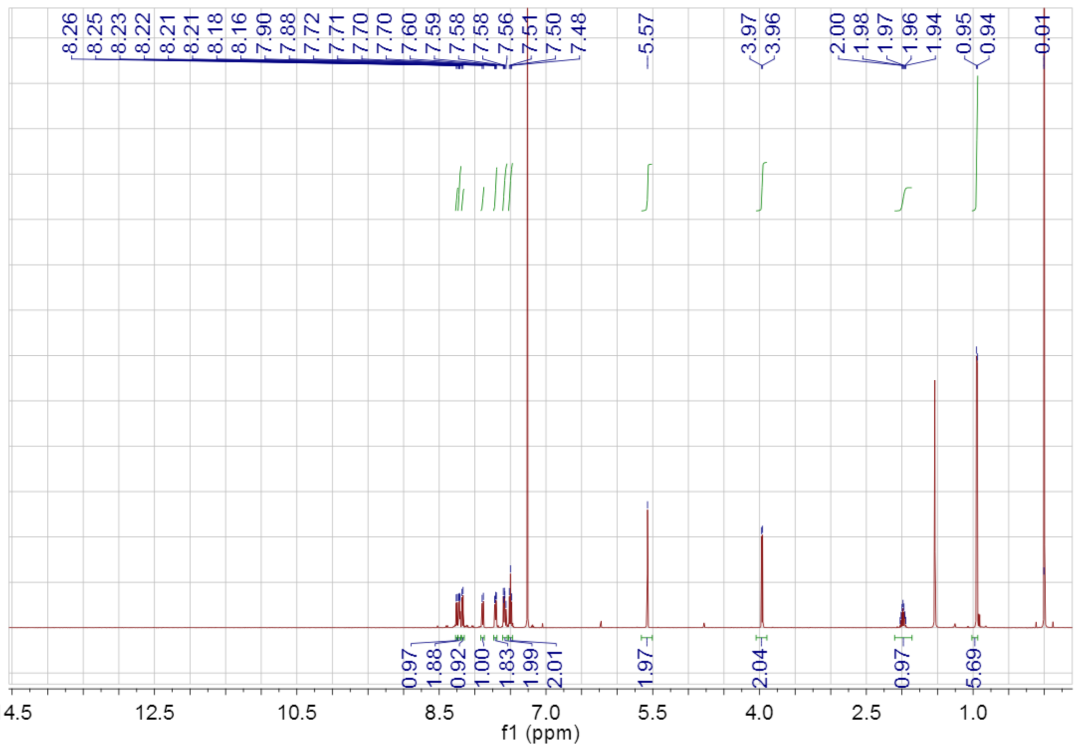

Supplementary Figure 49. ^{1}H NMR of **9** (500 MHz, $CDCl_3$).

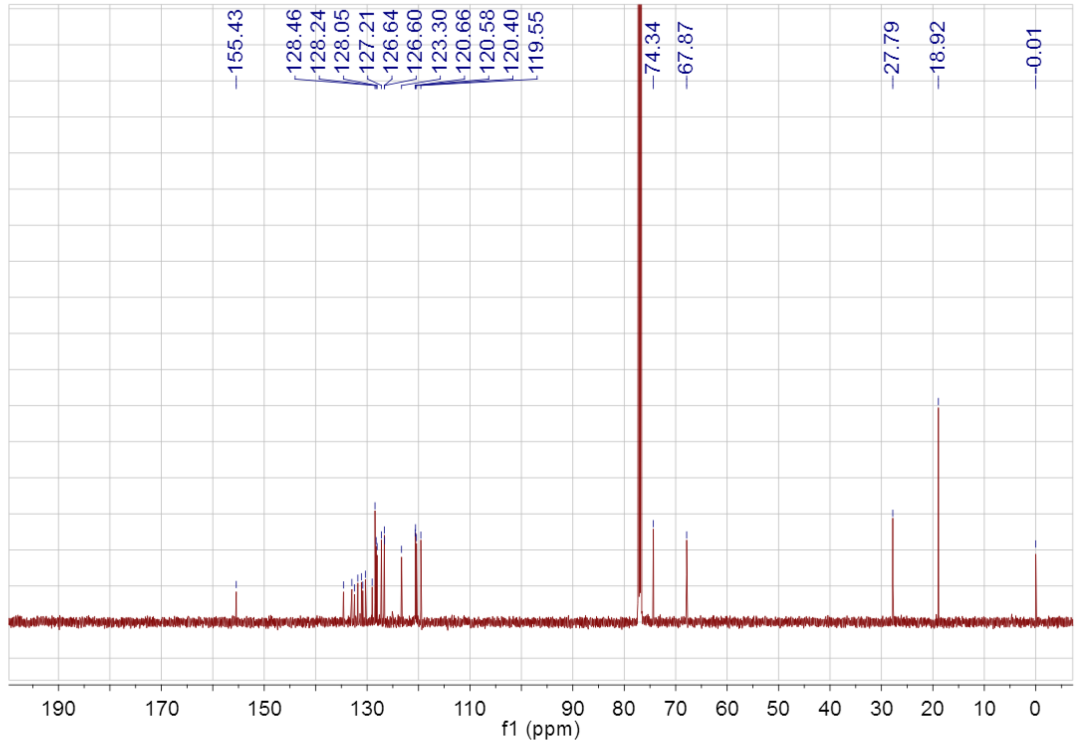

Supplementary Figure 50. ^{13}C NMR of **9** (125 MHz, $CDCl_3$).

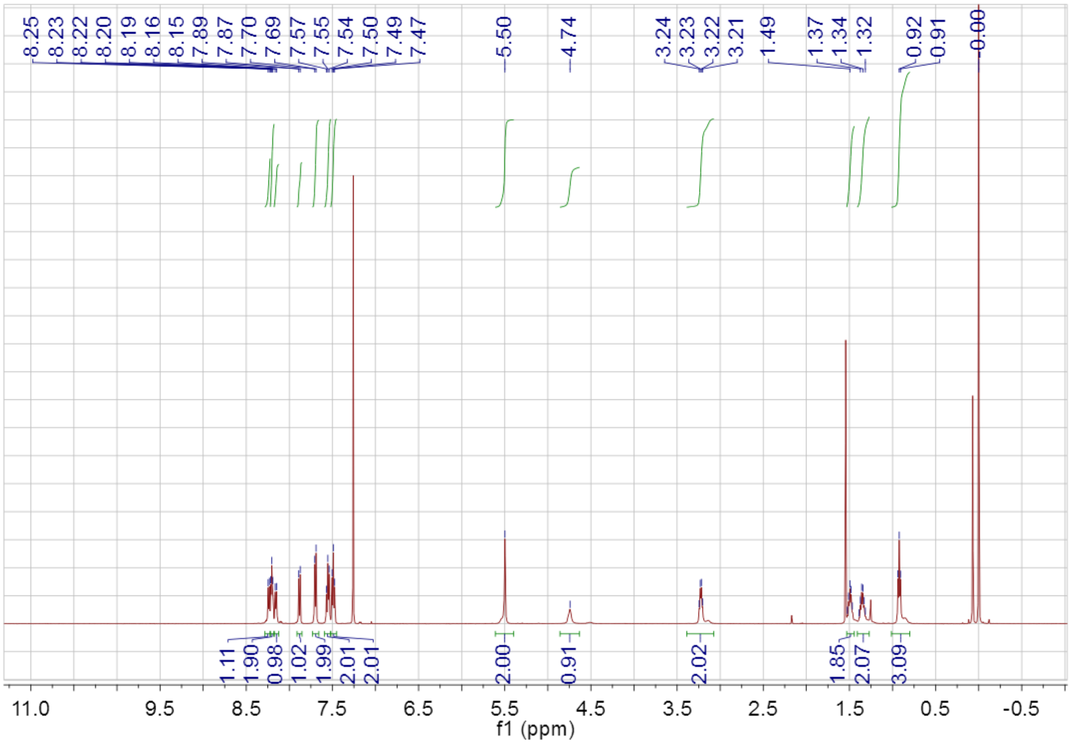

Supplementary Figure 51. ^{1}H NMR of **10** (500 MHz, $CDCl_3$).

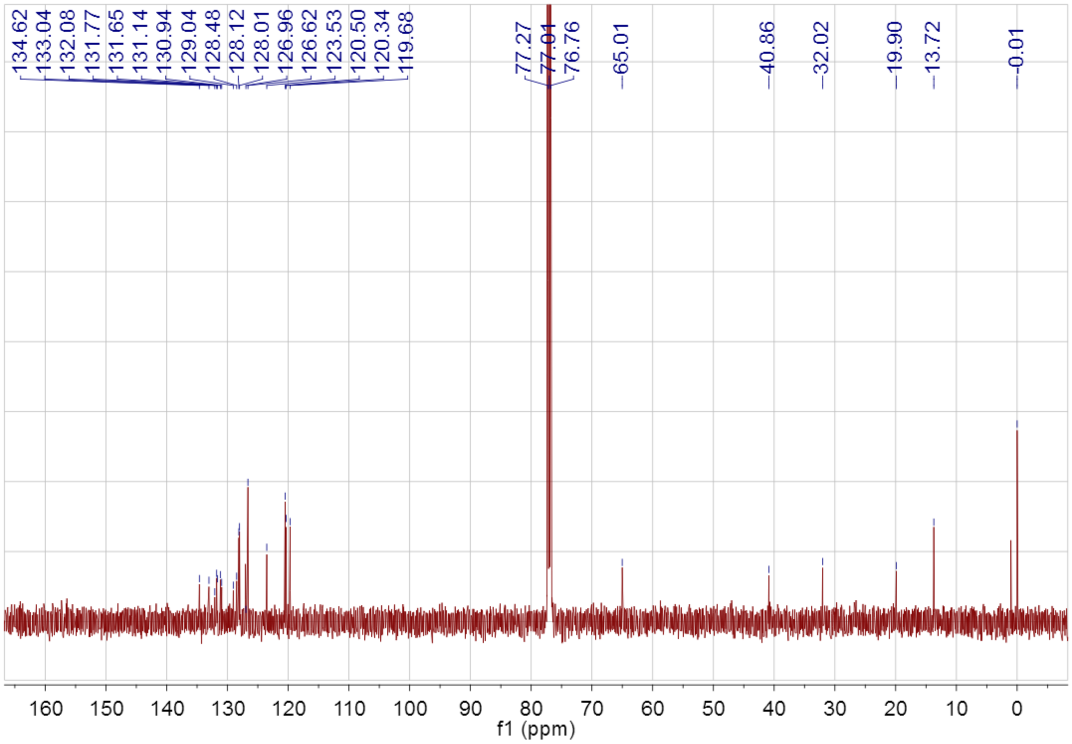

Supplementary Figure 52. ^{13}C NMR of **10** (125 MHz, $CDCl_3$).

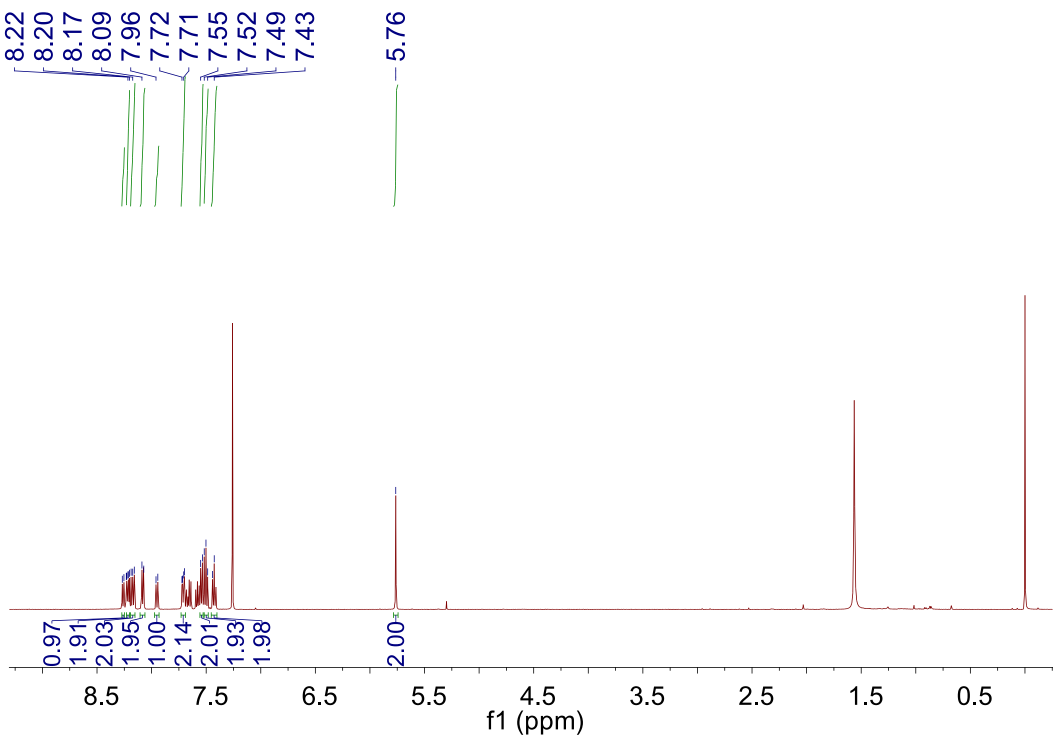

Supplementary Figure 53. ^{1}H NMR of **11** (500 MHz, $CDCl_3$).

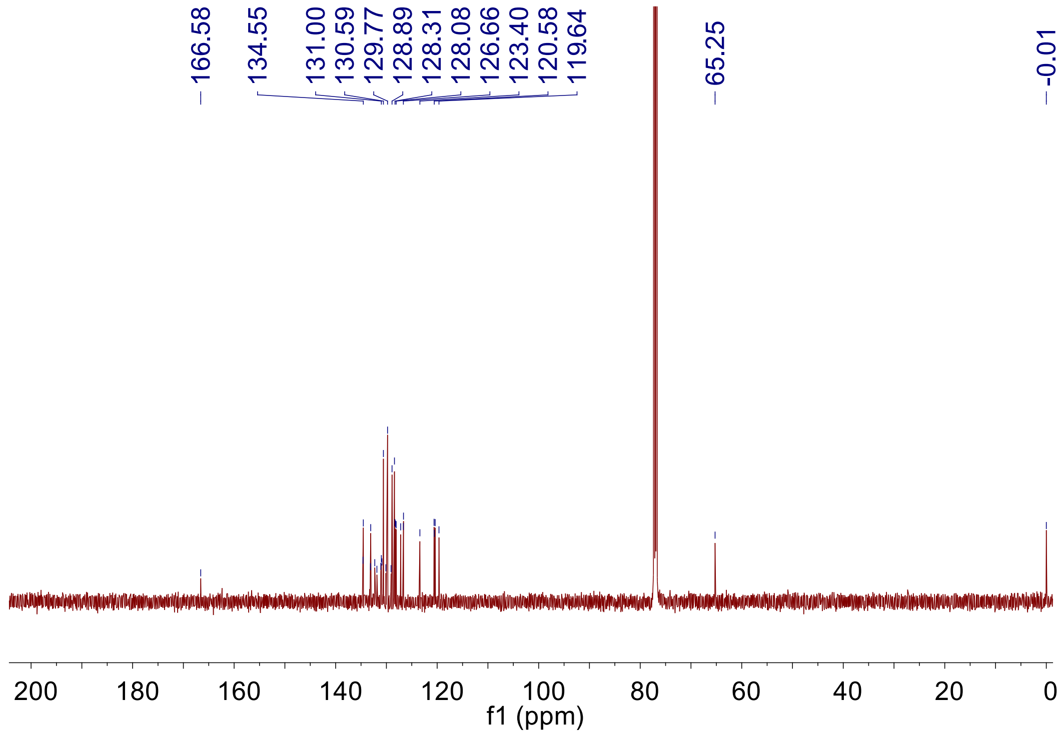

Supplementary Figure 54. ^{13}C NMR of **11** (125 MHz, $CDCl_3$).

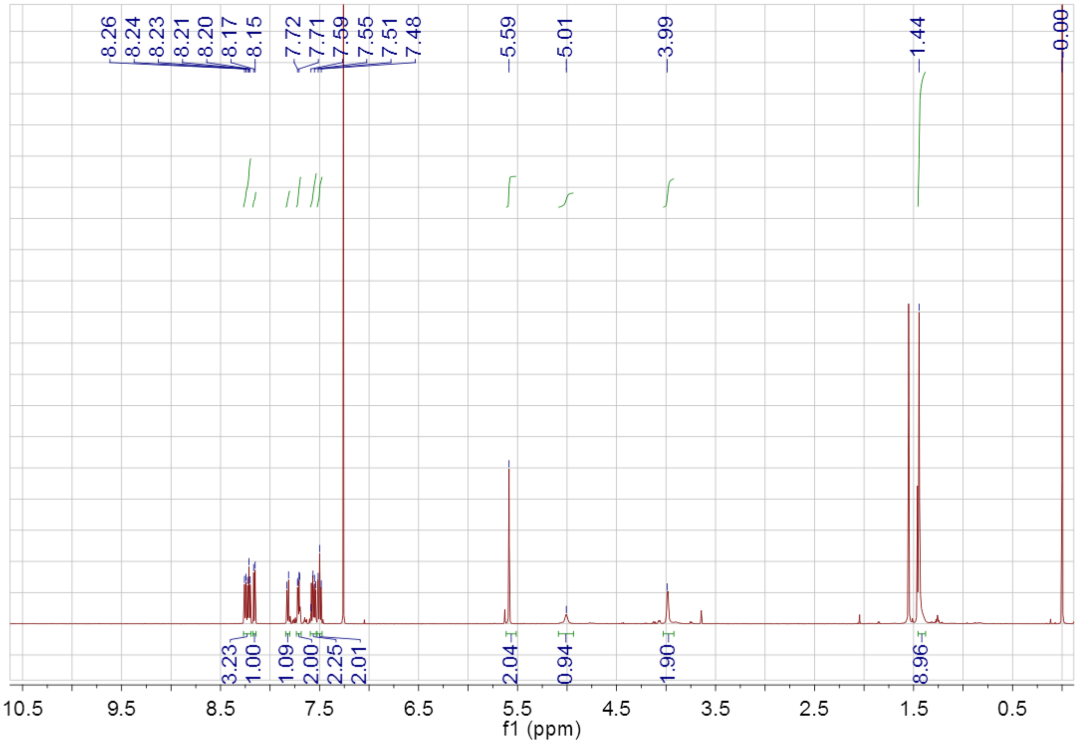

Supplementary Figure 55. ^{1}H NMR of **12** (500 MHz, $CDCl_3$).

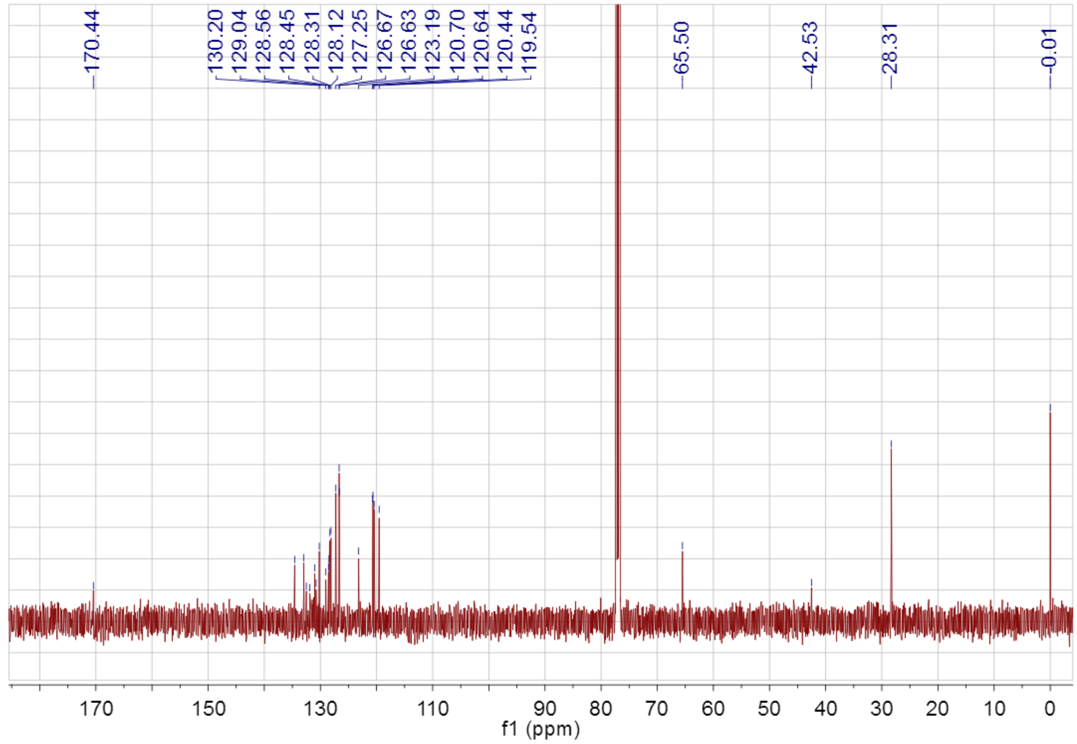

Supplementary Figure 56. ^{13}C NMR of **12** (125 MHz, $CDCl_3$).

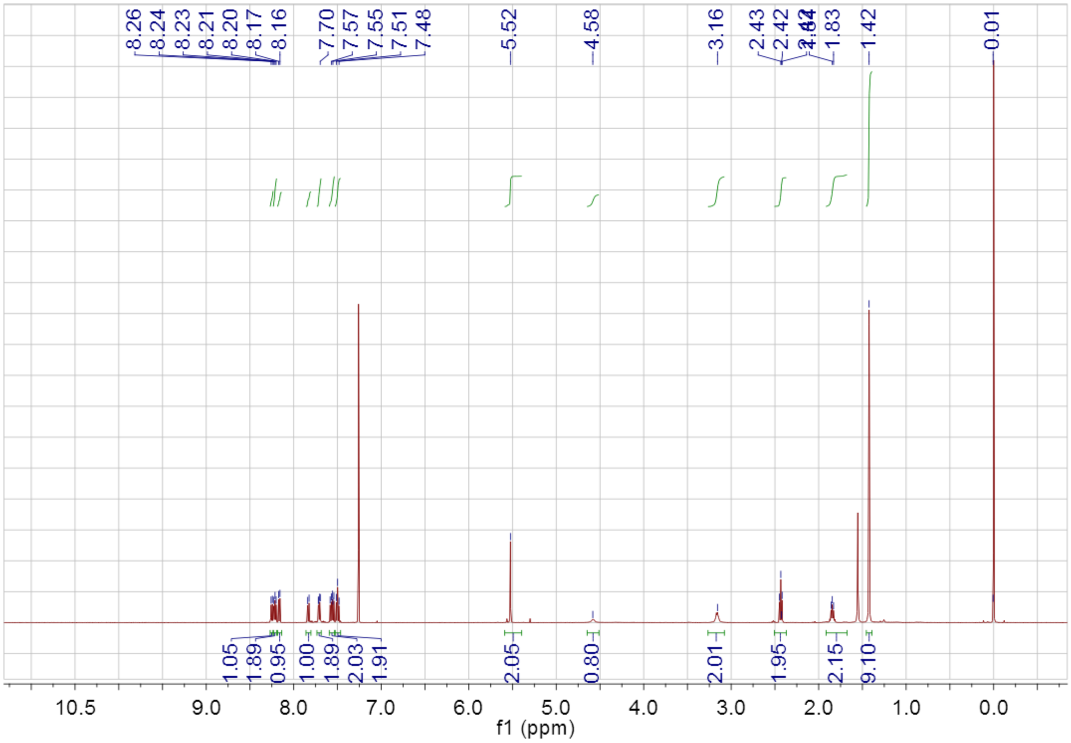

Supplementary Figure 57. ^{1}H NMR of **13** (500 MHz, $CDCl_3$).

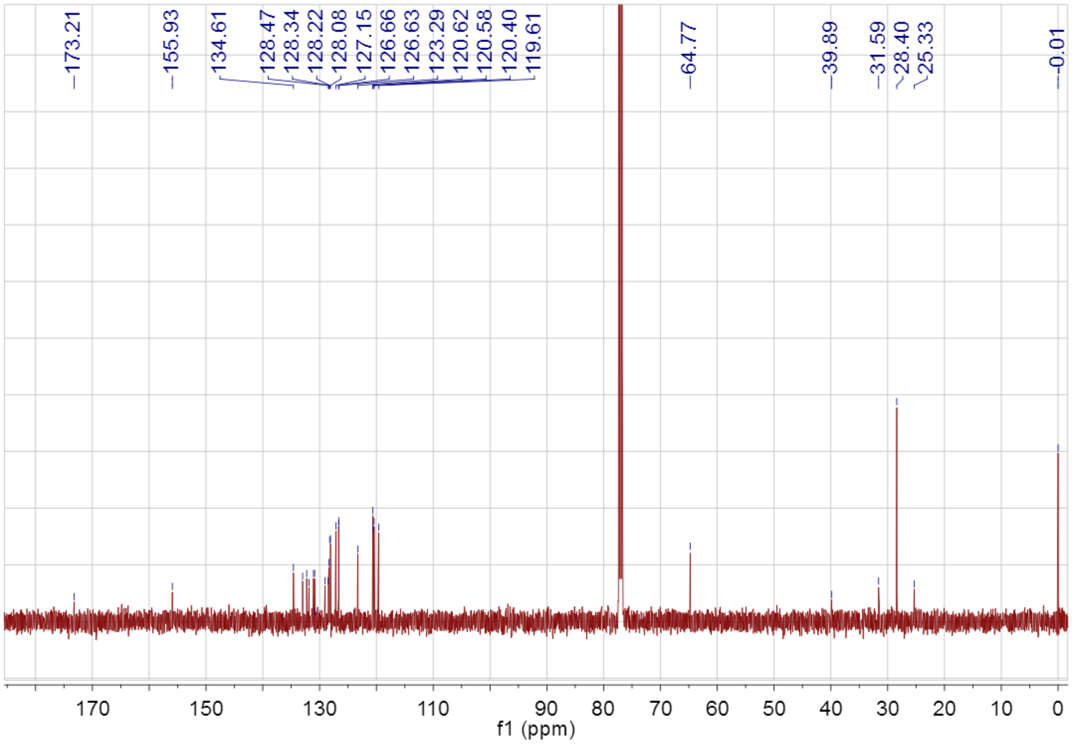

Supplementary Figure 58. ^{13}C NMR of **13** (125 MHz, $CDCl_3$).

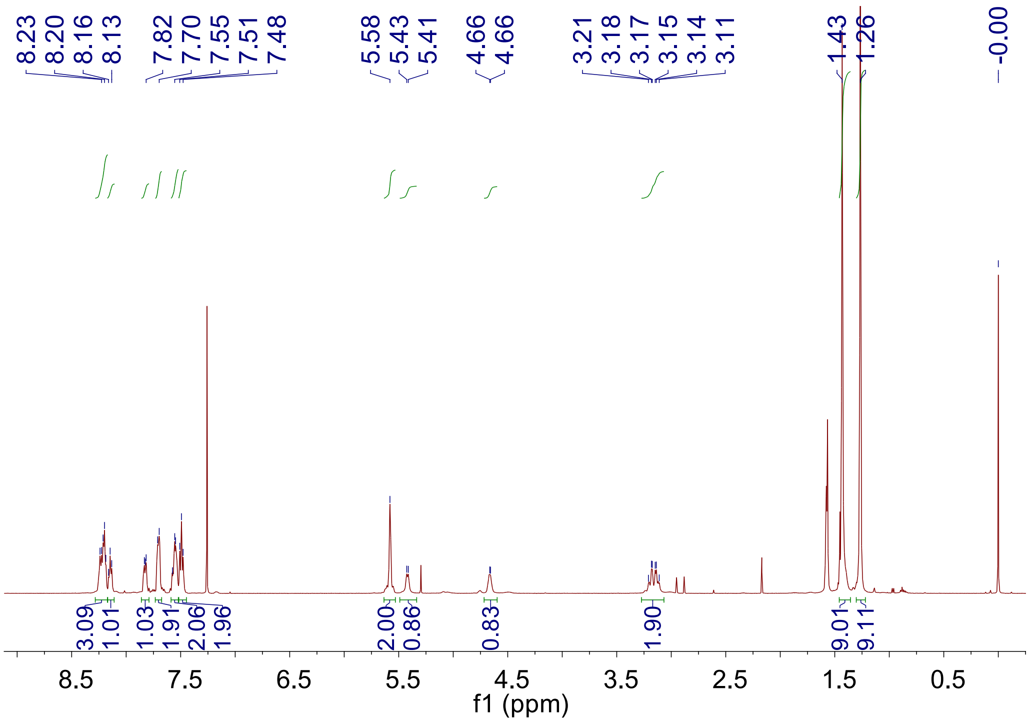

Supplementary Figure 59. ^{1}H NMR of **14** (500 MHz, $CDCl_3$).

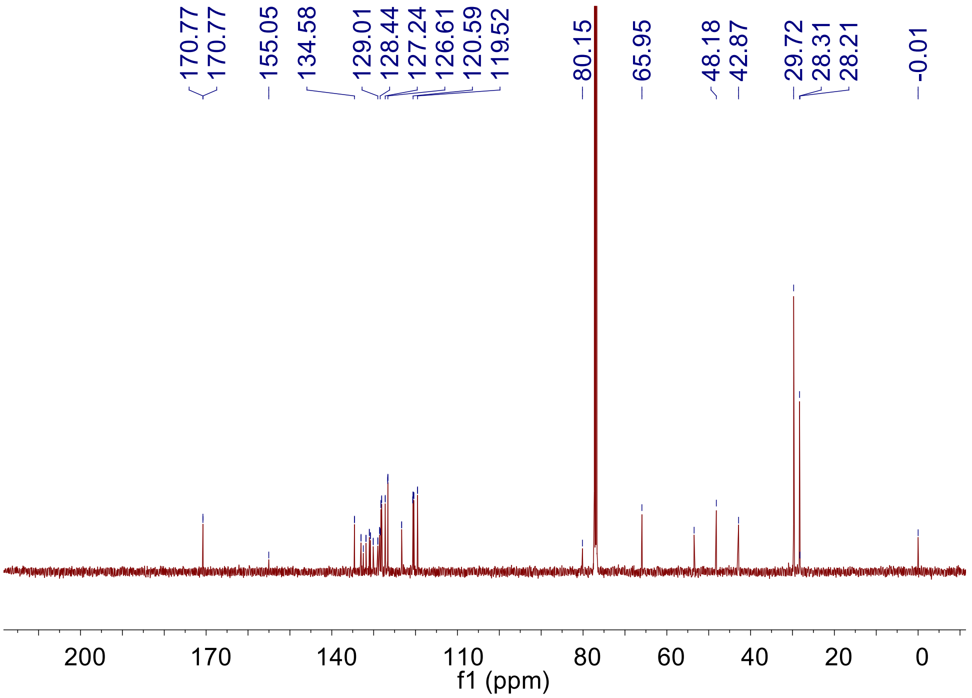

Supplementary Figure 60. ^{13}C NMR of **14** (125 MHz, $CDCl_3$).

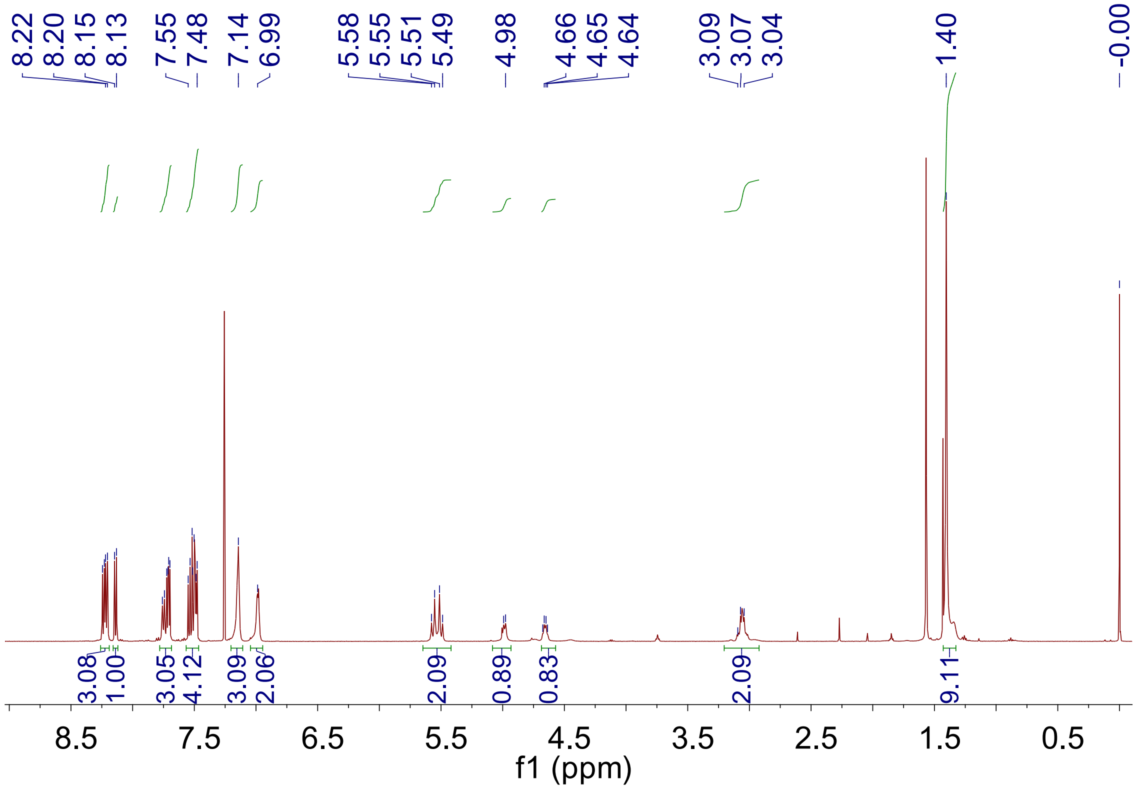

Supplementary Figure 61. ^{1}H NMR of **15** (500 MHz, $CDCl_3$).

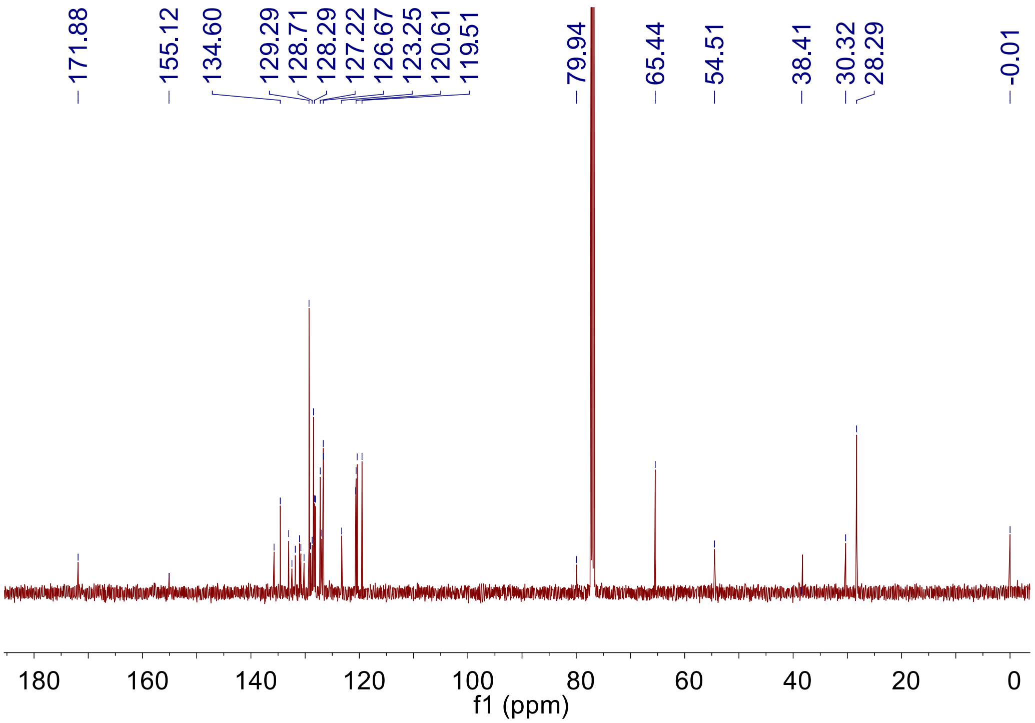

Supplementary Figure 62. ^{13}C NMR of **15** (125 MHz, $CDCl_3$).

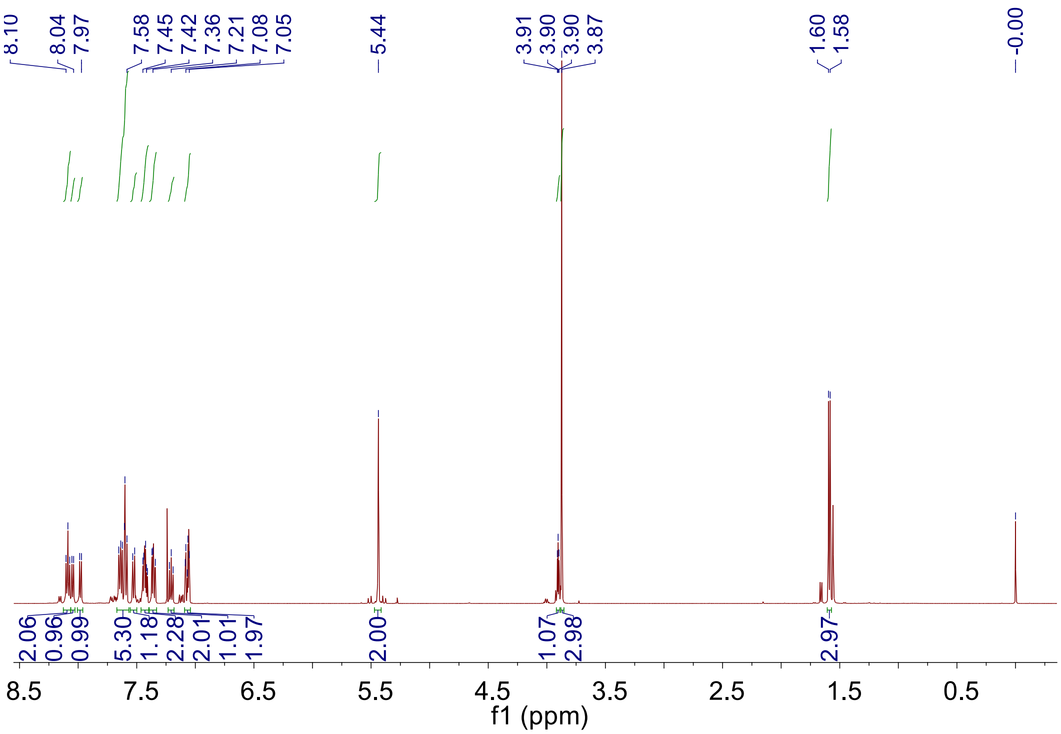

Supplementary Figure 63. ^{1}H NMR of **16** (500 MHz, $CDCl_3$).

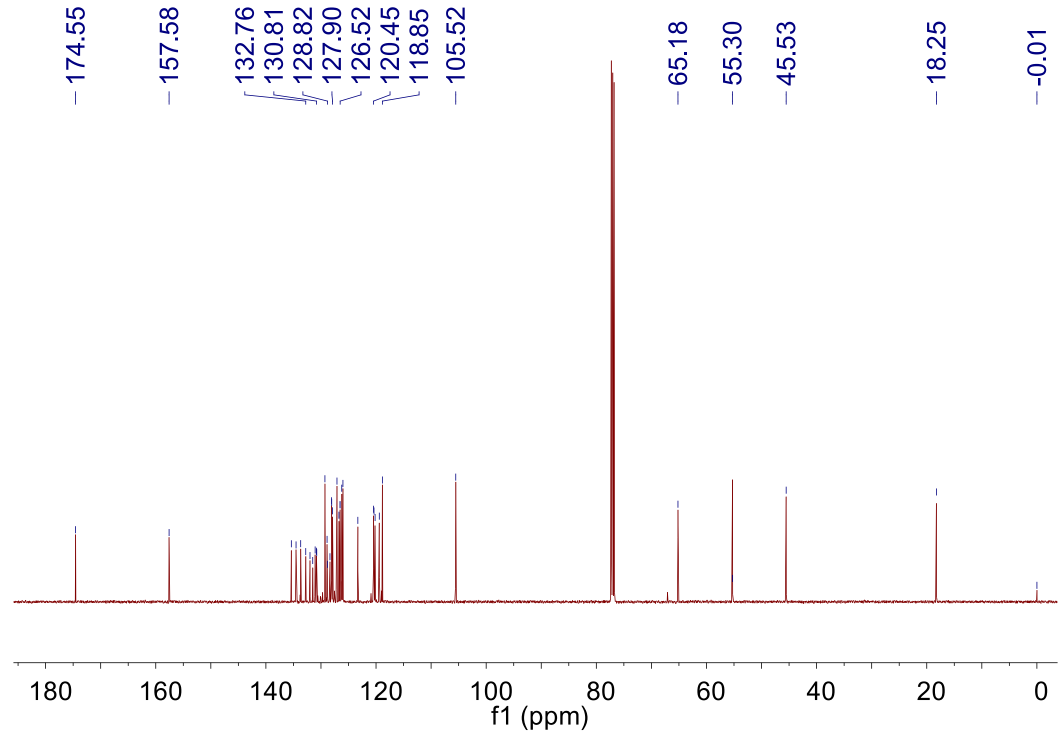

Supplementary Figure 64. ^{13}C NMR of **16** (125 MHz, $CDCl_3$).

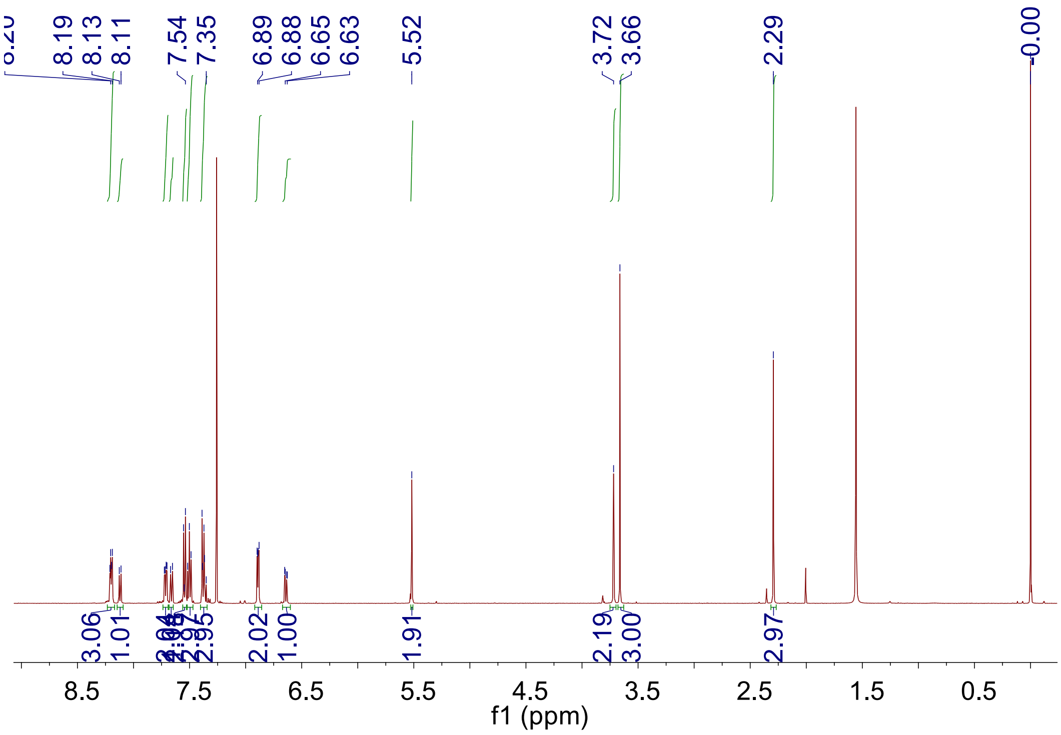

Supplementary Figure 65. ^{1}H NMR of **17** (500 MHz, $CDCl_3$).

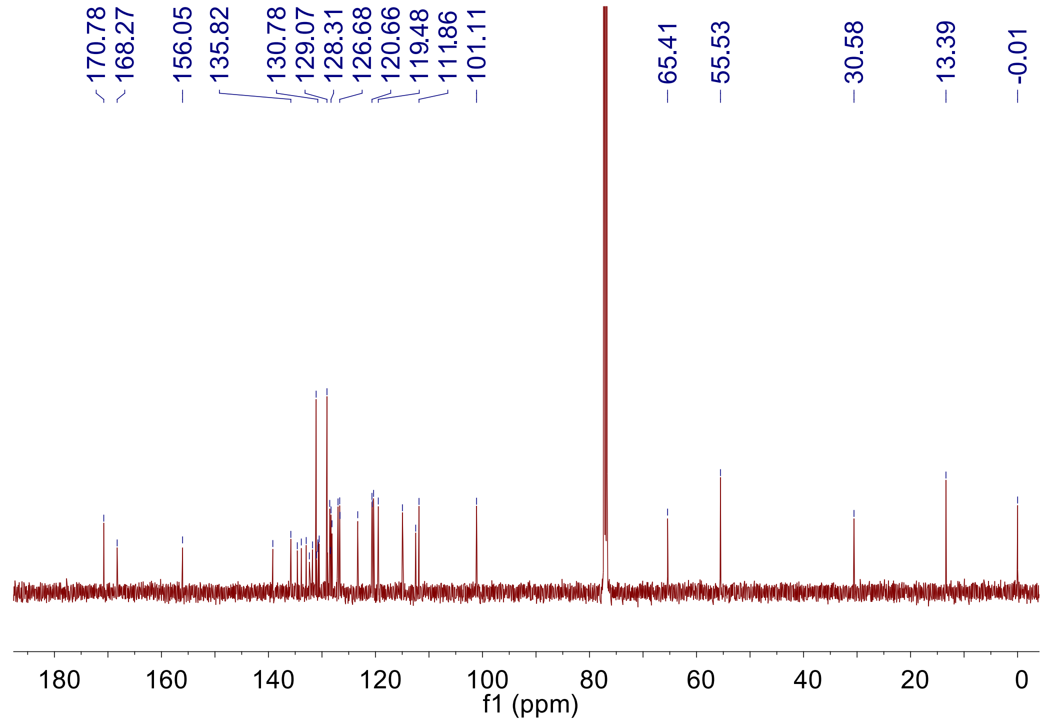

Supplementary Figure 66. ^{13}C NMR of **17** (125 MHz, $CDCl_3$).

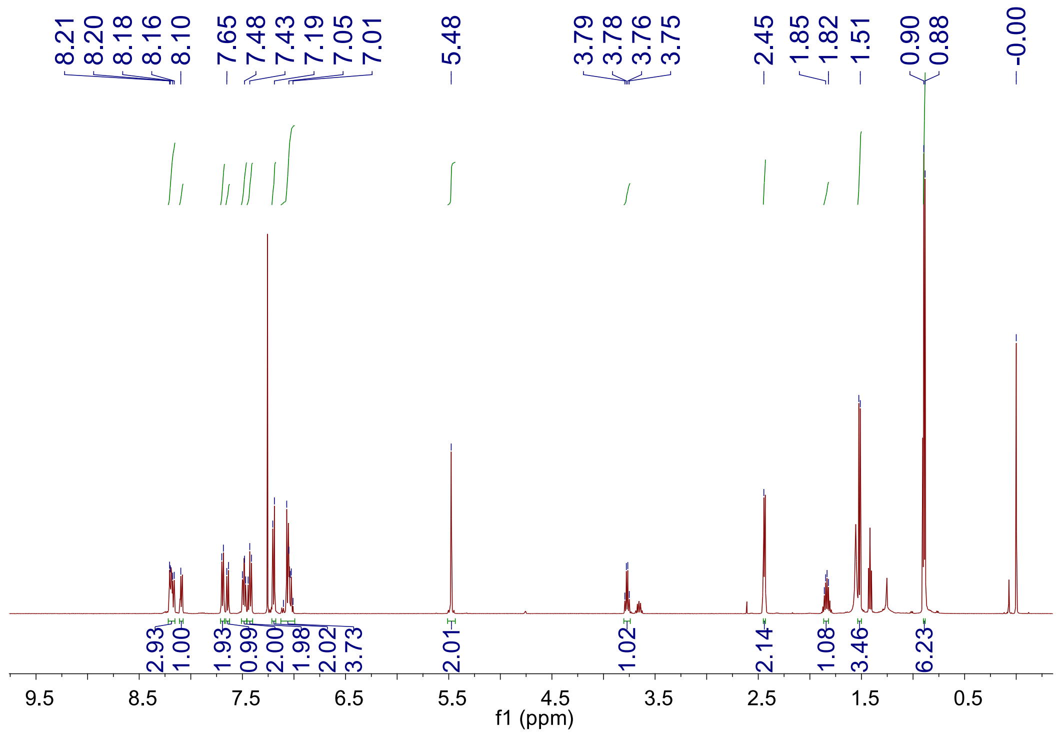

Supplementary Figure 67. ^{1}H NMR of **18** (500 MHz, $CDCl_3$).

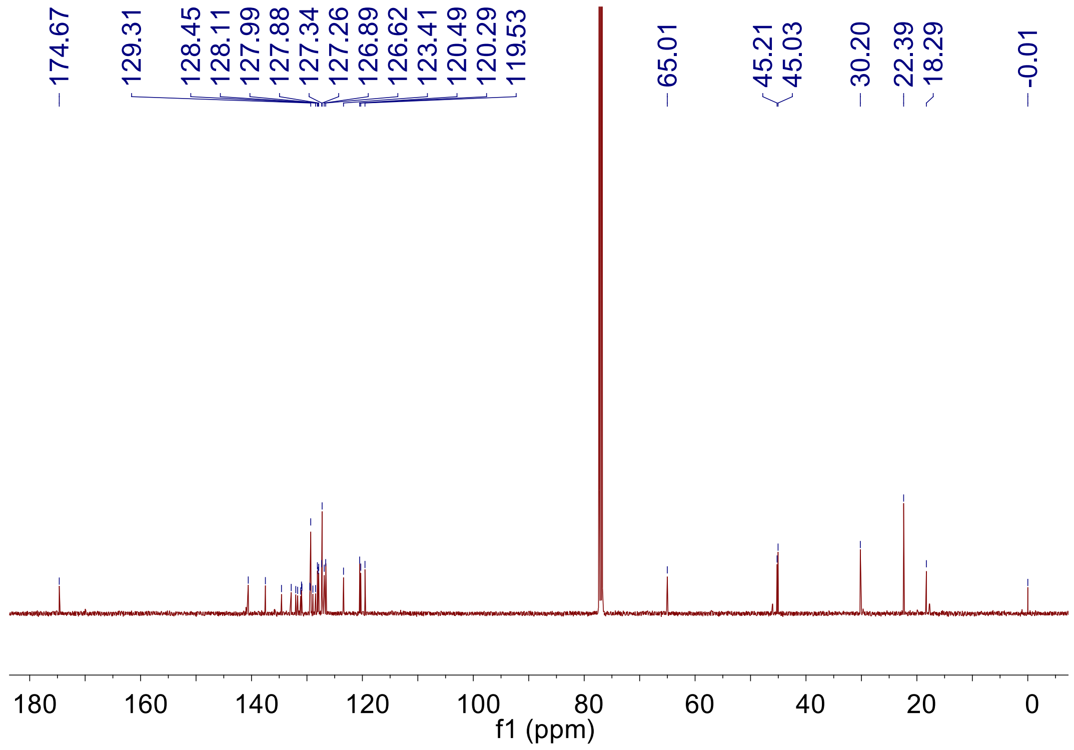

Supplementary Figure 68. ^{13}C NMR of **18** (125 MHz, $CDCl_3$).

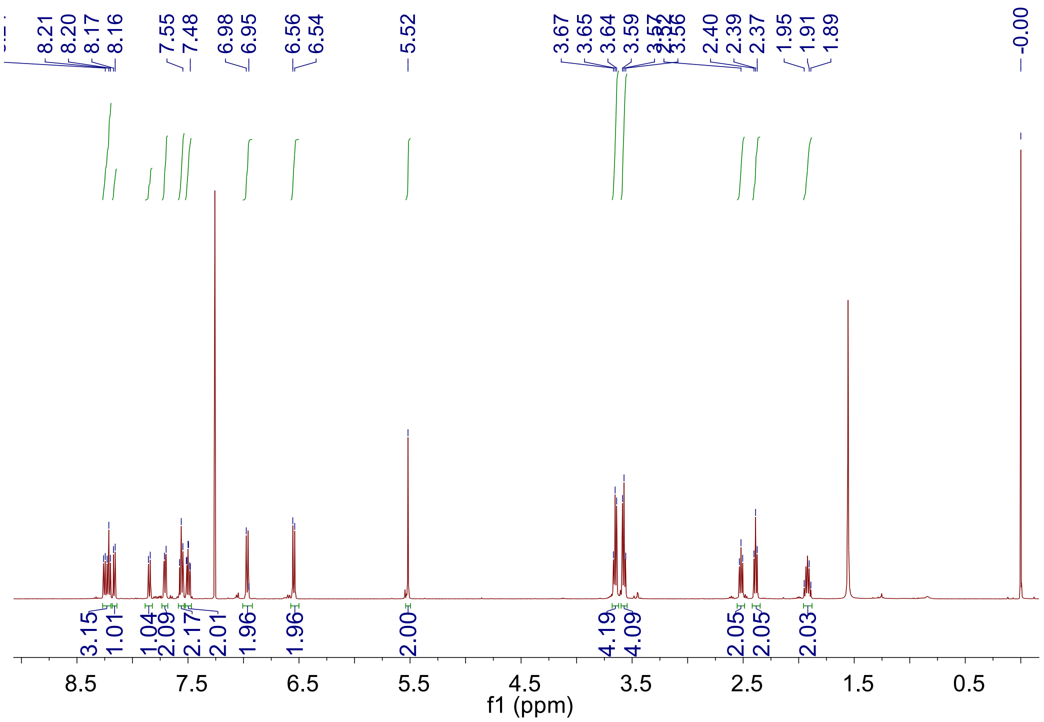

Supplementary Figure 69. ^{1}H NMR of **19** (500 MHz, $CDCl_3$).

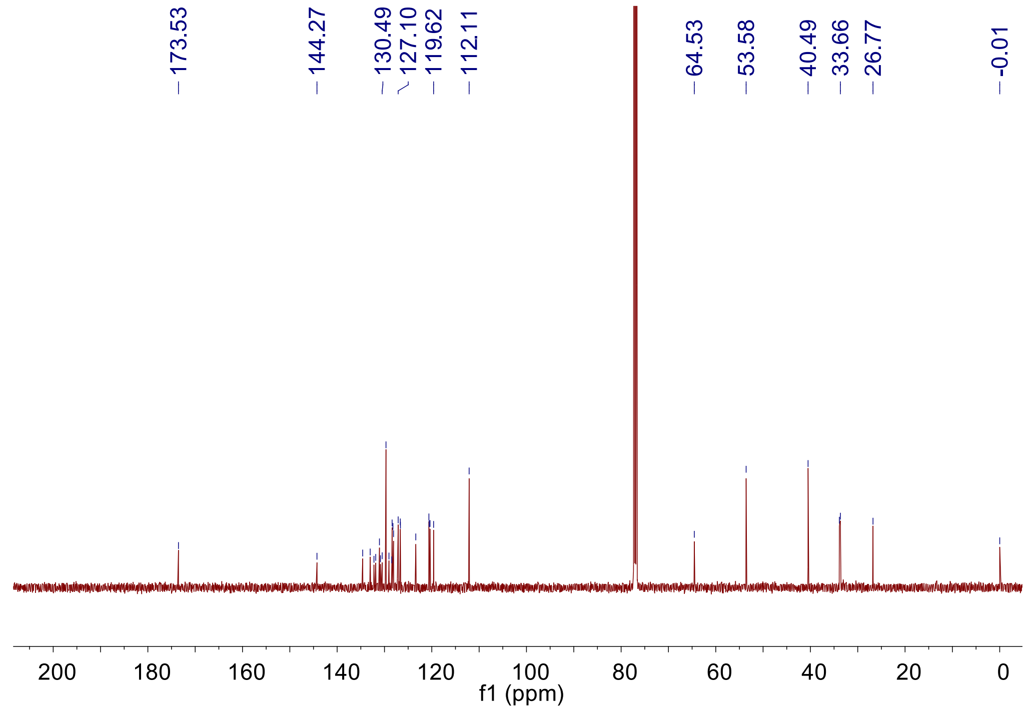

Supplementary Figure 70. ^{13}C NMR of **19** (125 MHz, $CDCl_3$).

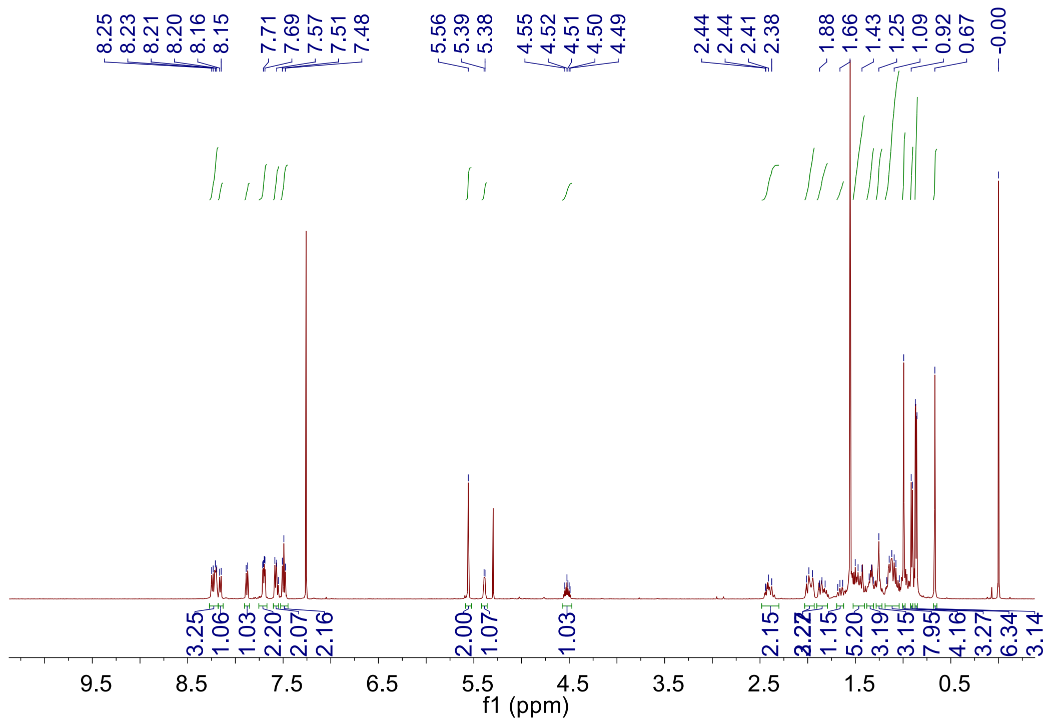

Supplementary Figure 71. ^{1}H NMR of **20** (500 MHz, $CDCl_3$).

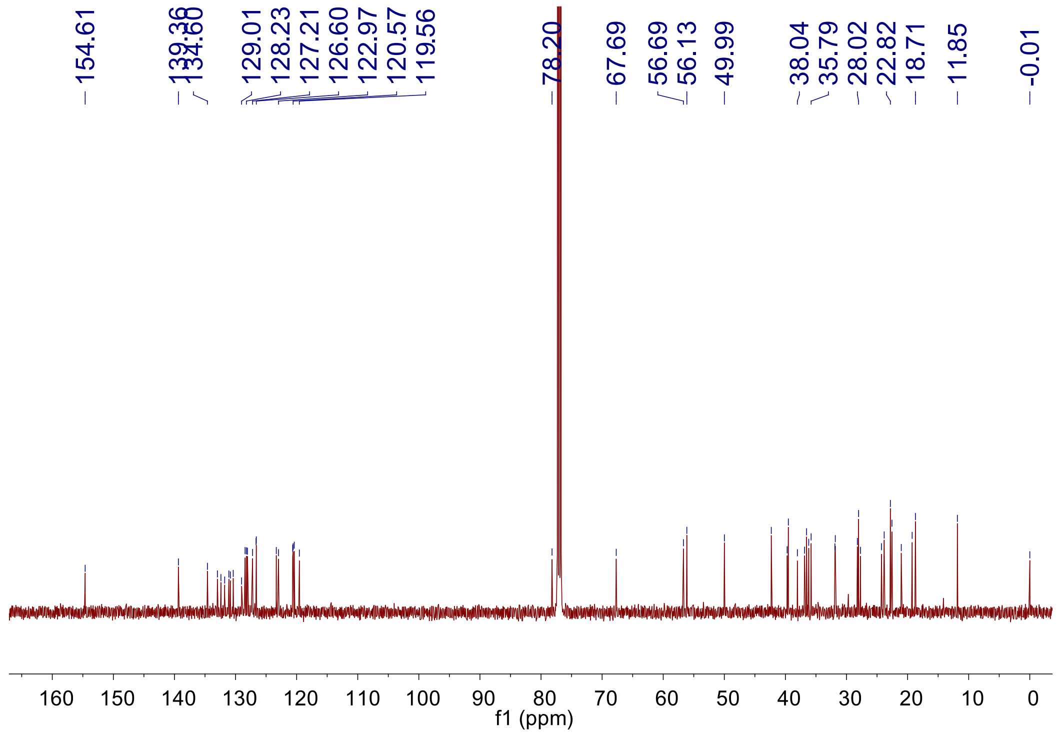

Supplementary Figure 72. ^{13}C NMR of **20** (125 MHz, $CDCl_3$).

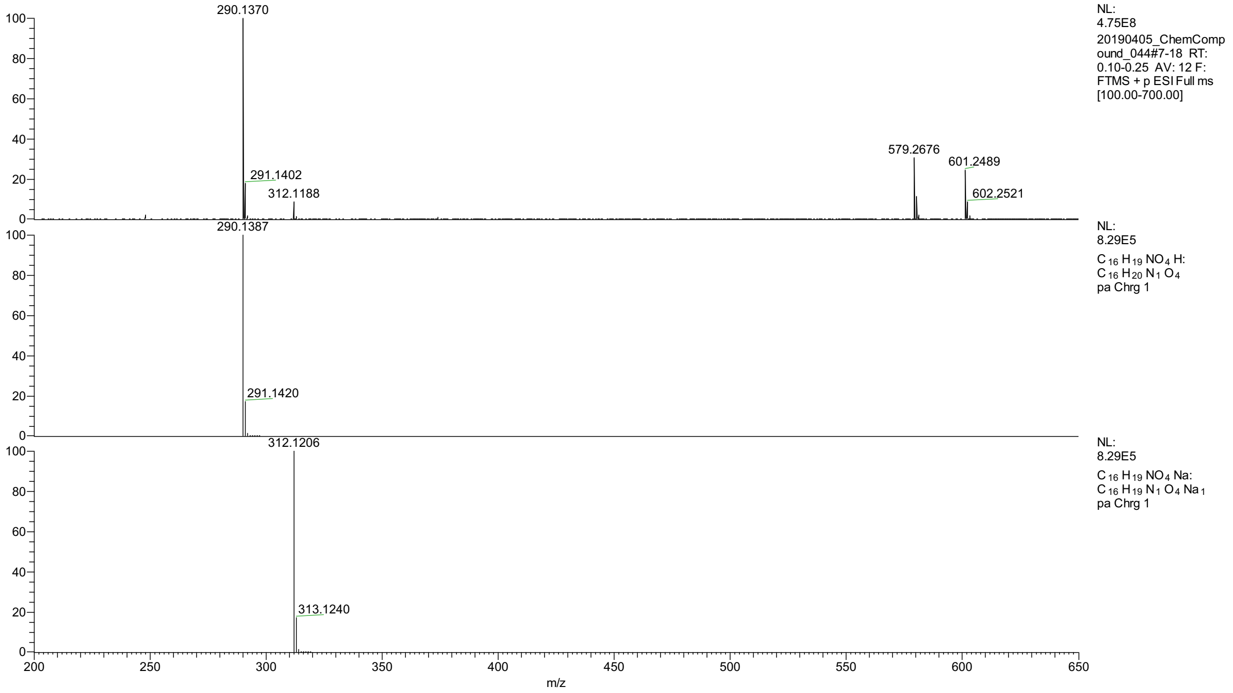

Supplementary Figure 73. HRMS (ESI) of **1**.

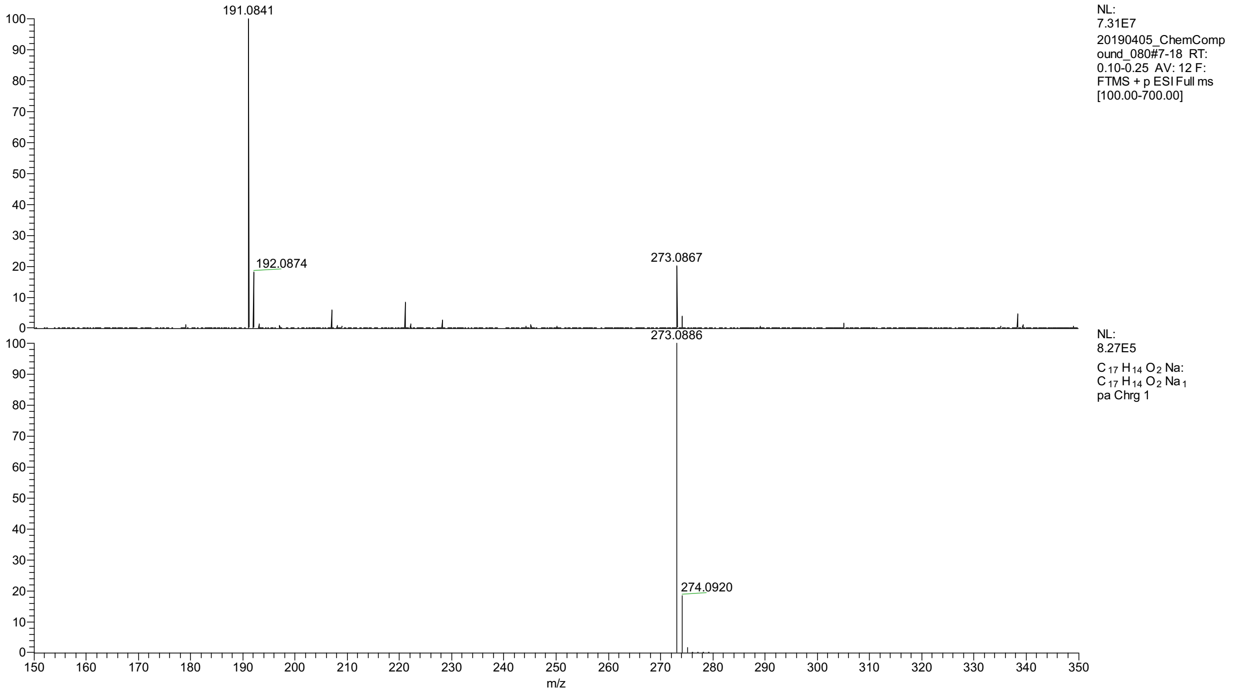

Supplementary Figure 74. HRMS (ESI) of **2**.

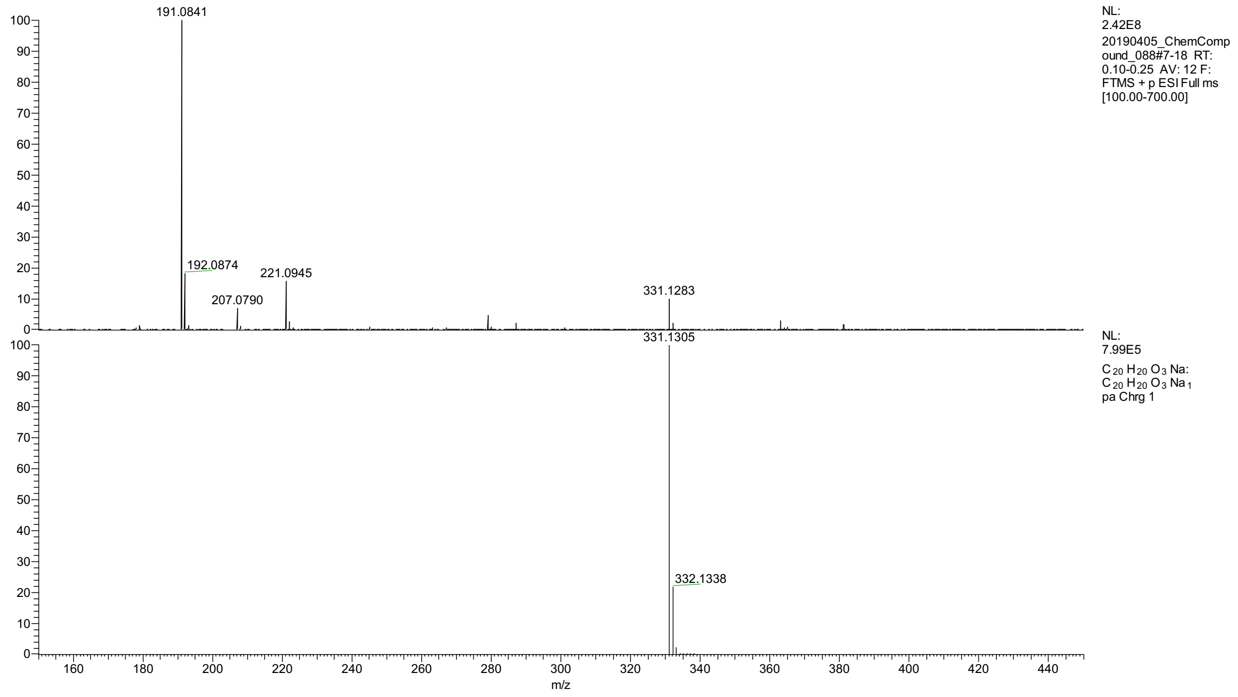

Supplementary Figure 75. HRMS (ESI) of **3**.

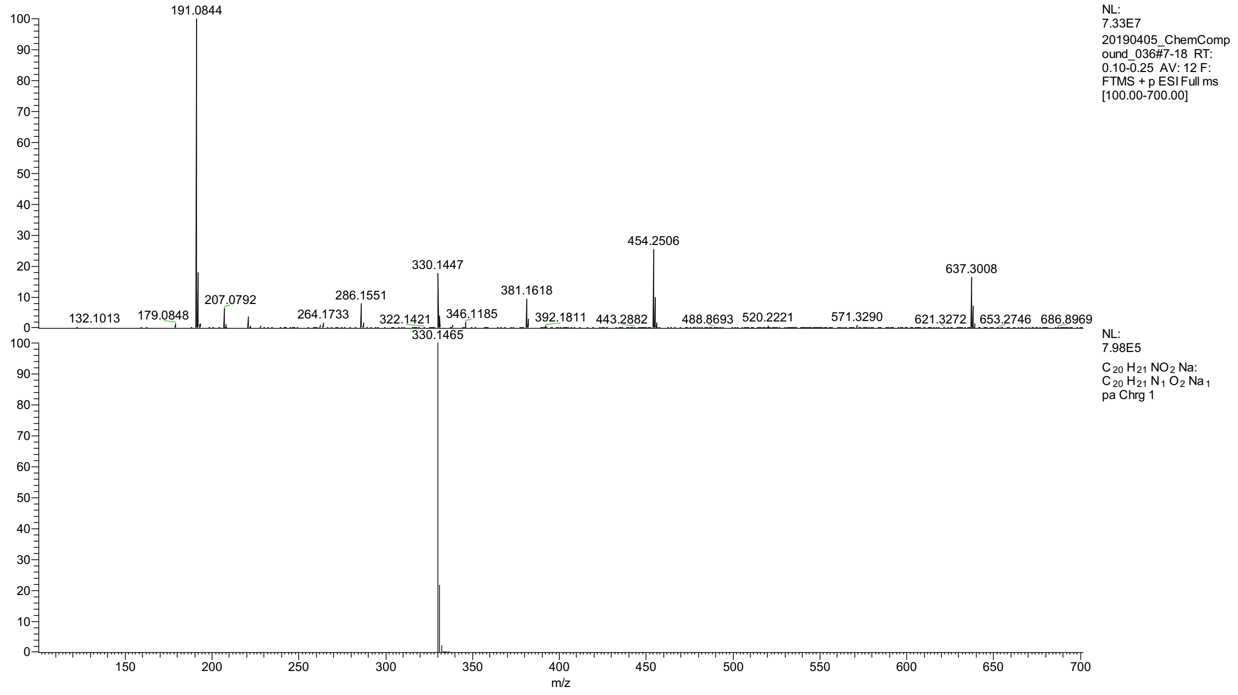

Supplementary Figure 76. HRMS (ESI) of **4**.

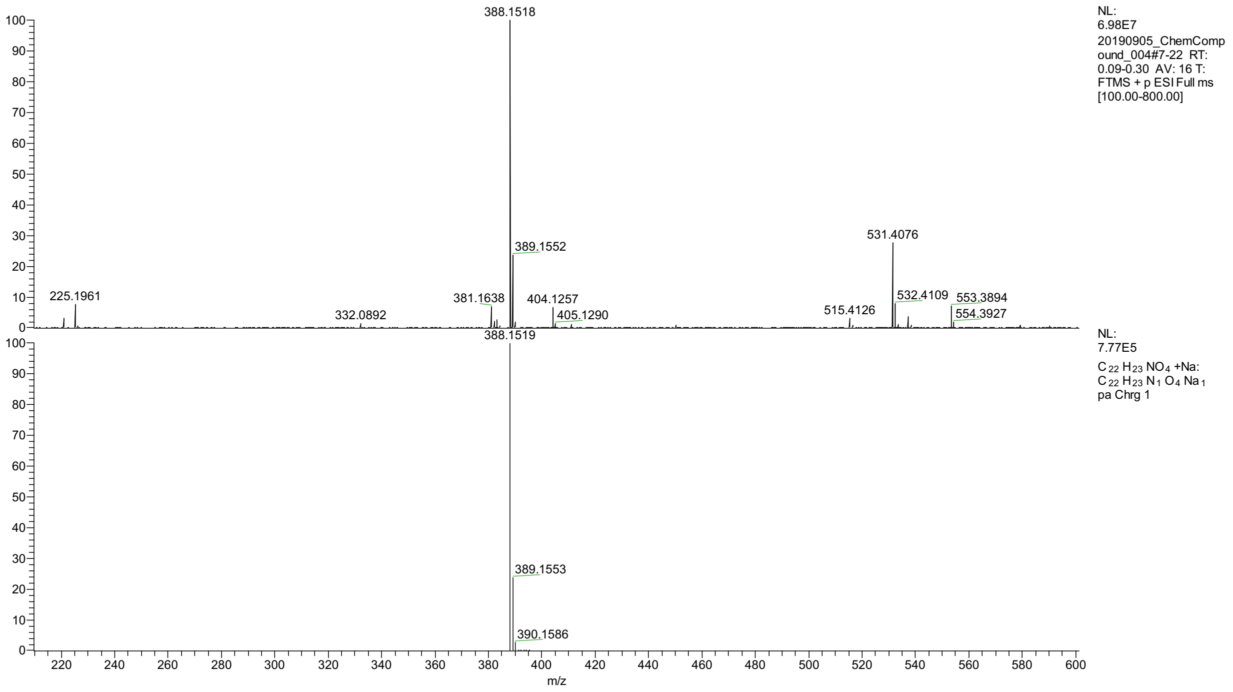

Supplementary Figure 77. HRMS (ESI) of **5**.

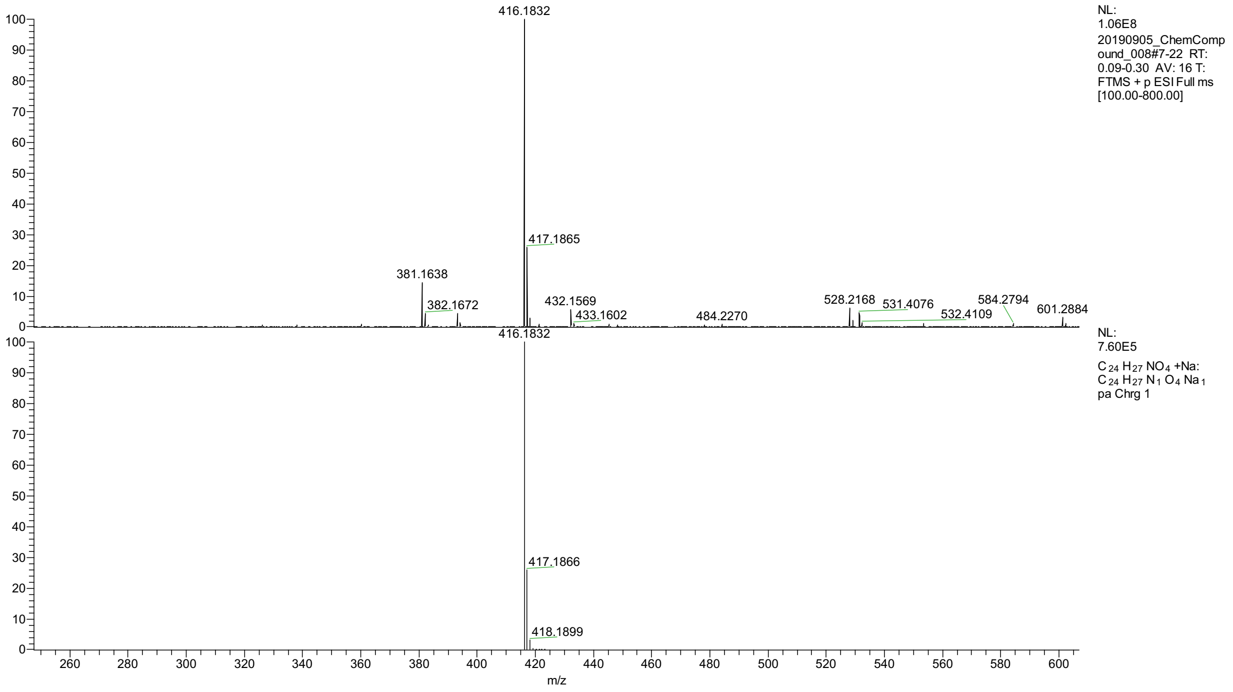

Supplementary Figure 78. HRMS (ESI) of **6**.

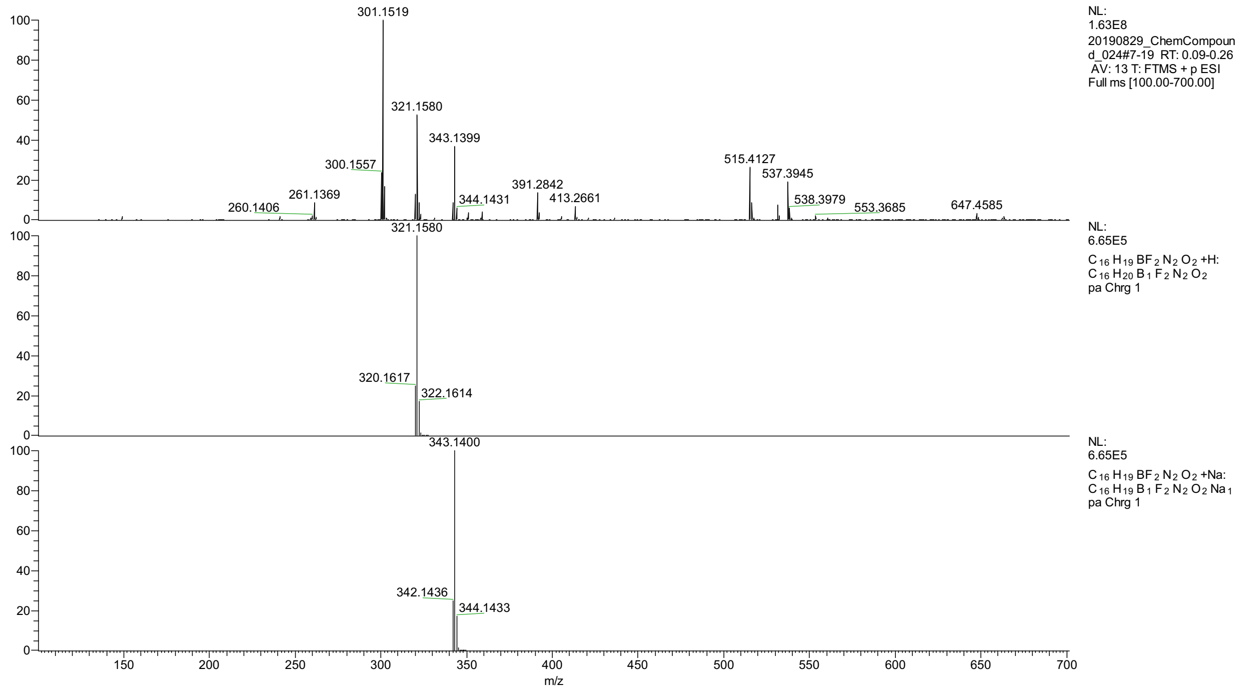

Supplementary Figure 79. HRMS (ESI) of **7**.

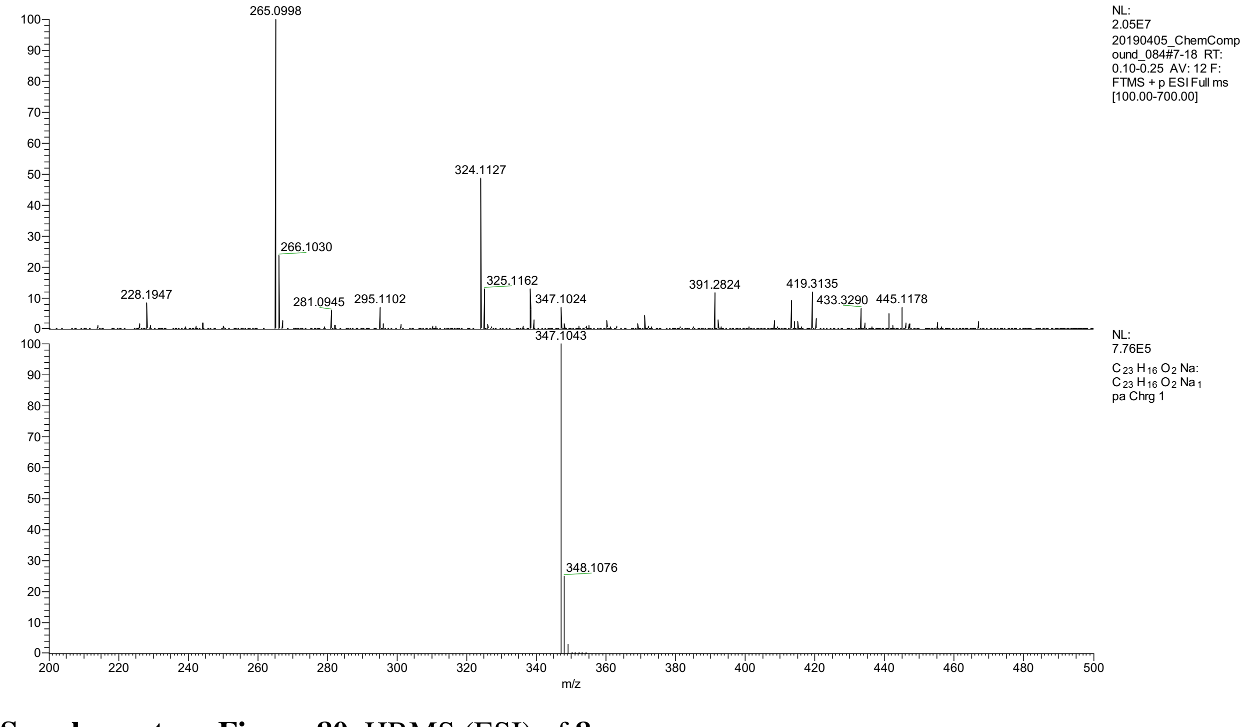

Supplementary Figure 80. HRMS (ESI) of **8**.

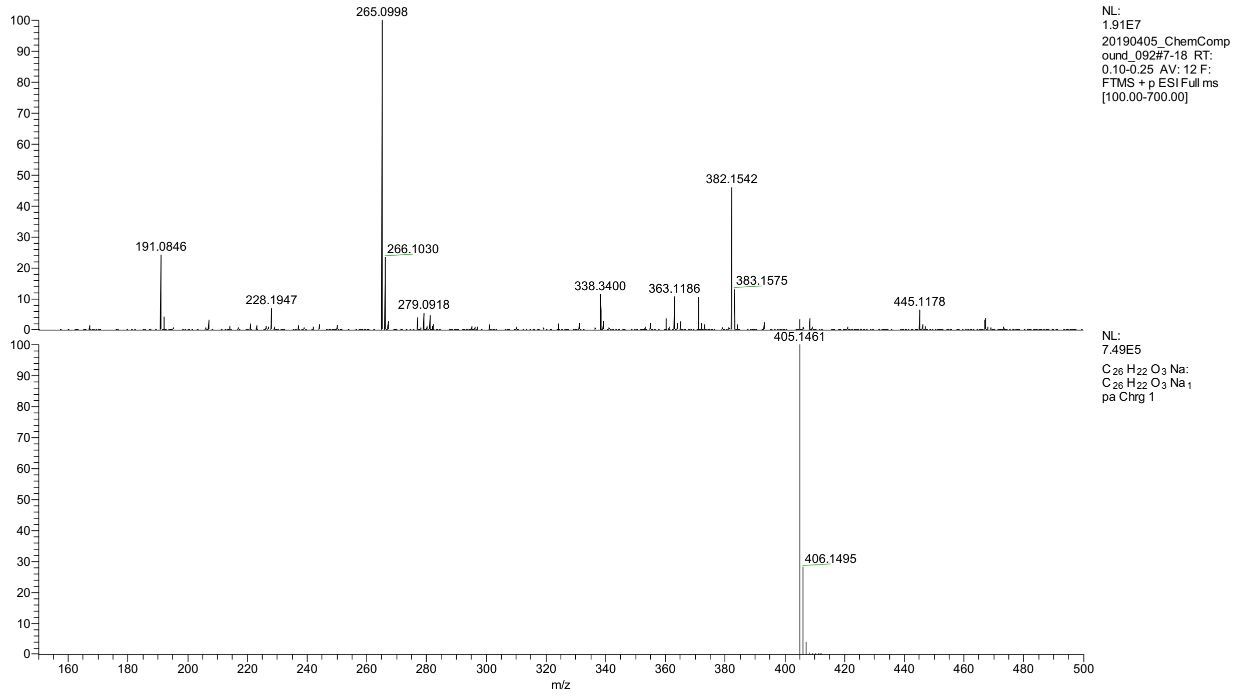

Supplementary Figure 81. HRMS (ESI) of **9**.

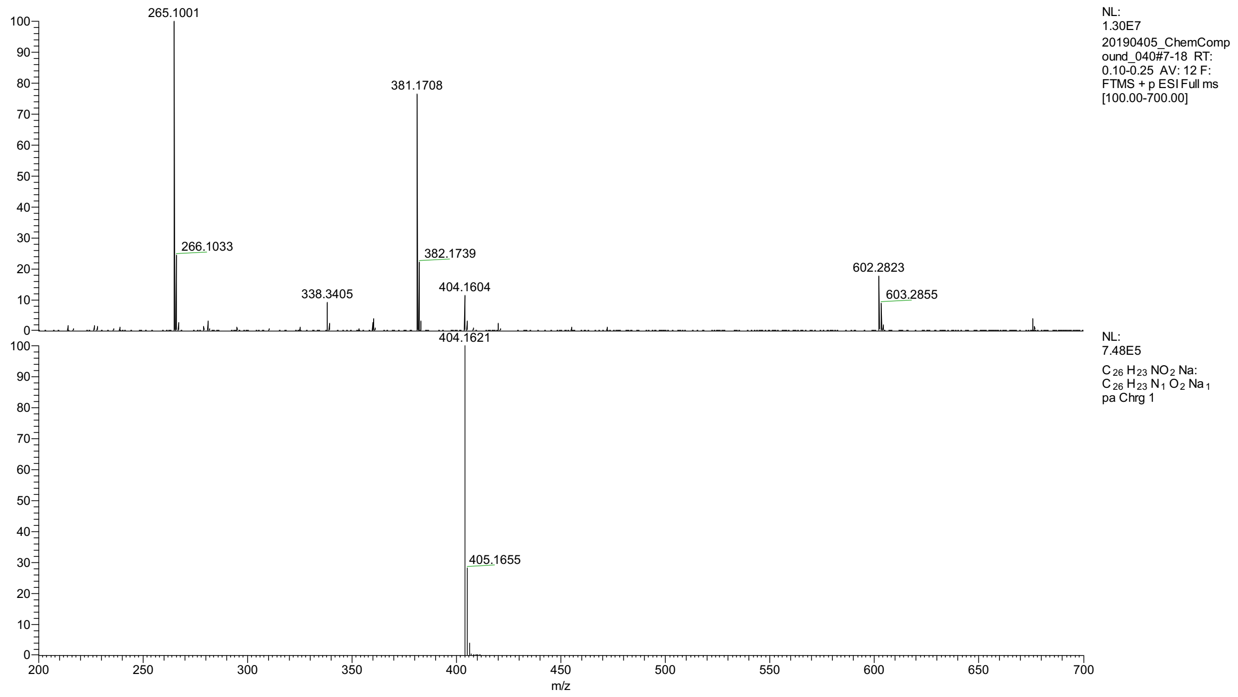

Supplementary Figure 82. HRMS (ESI) of **10**.

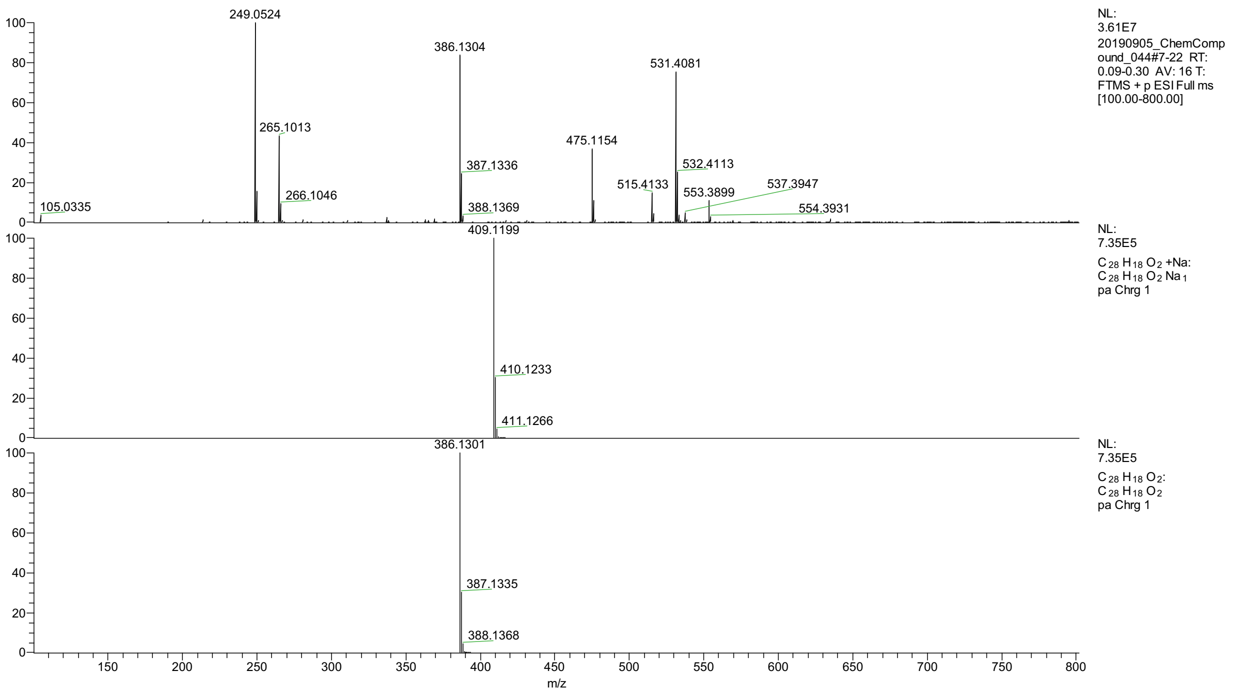

Supplementary Figure 83. HRMS (ESI) of **11**.

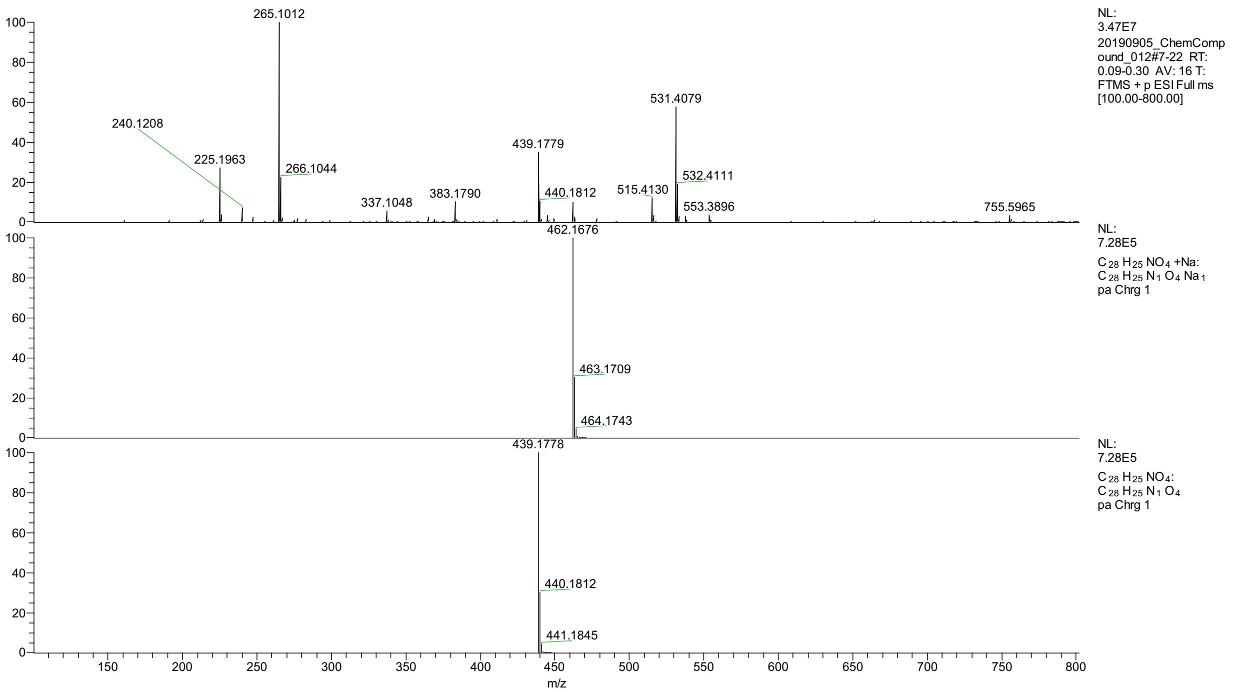

Supplementary Figure 84. HRMS (ESI) of **12**.

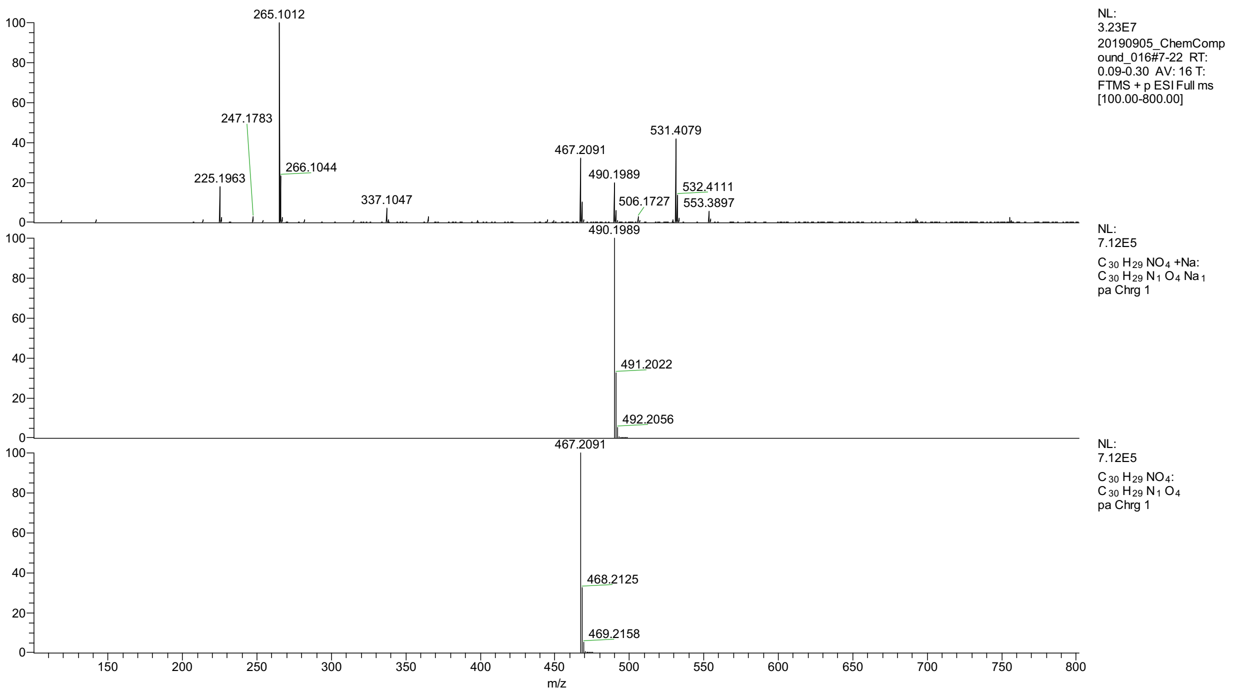

Supplementary Figure 85. HRMS (ESI) of **13**.

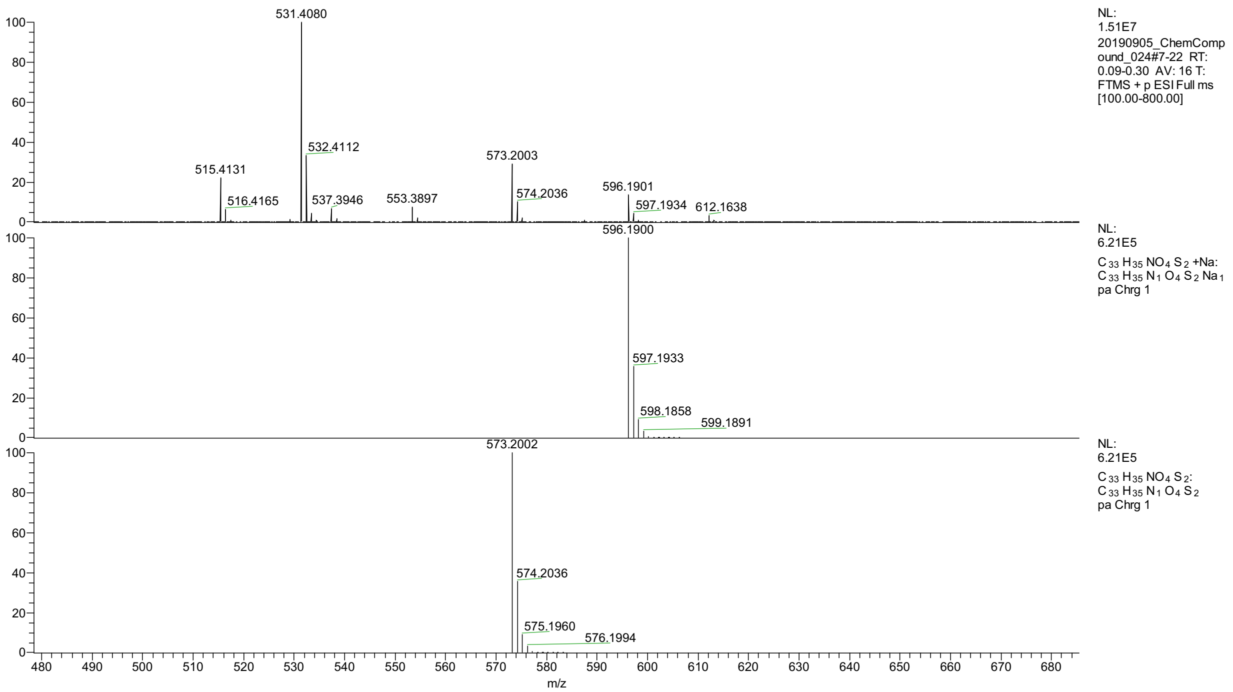

Supplementary Figure 86. HRMS (ESI) of **14**.

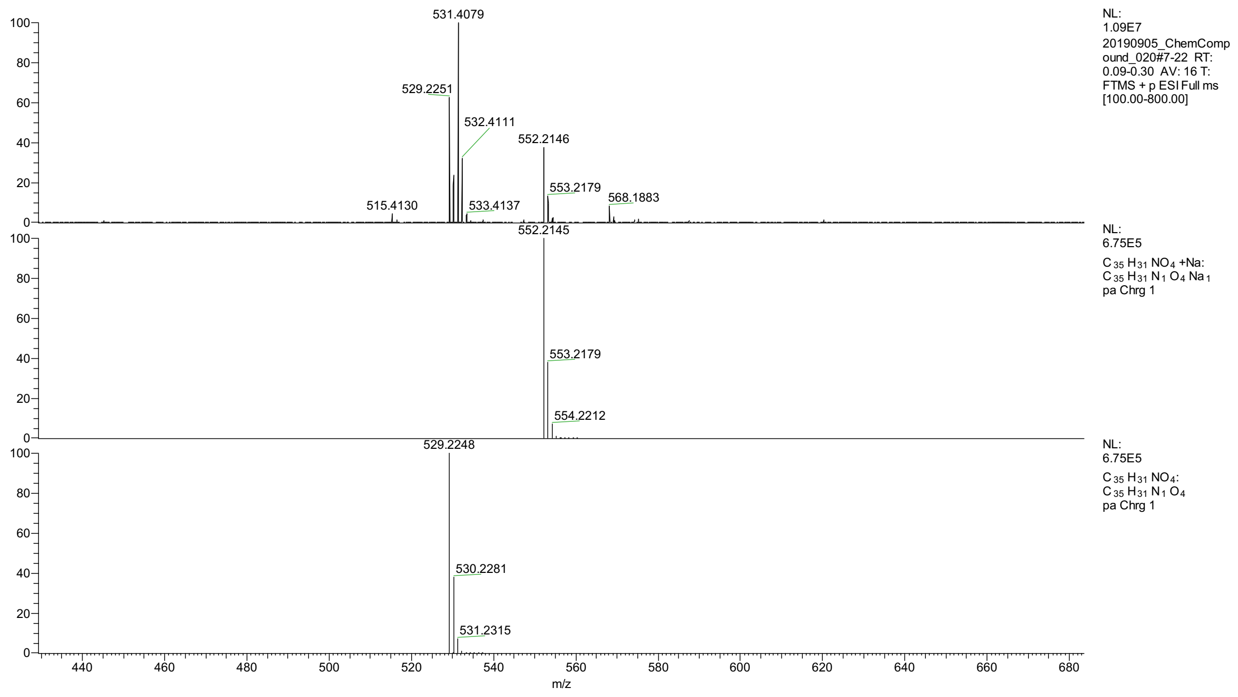

Supplementary Figure 87. HRMS (ESI) of **15**.

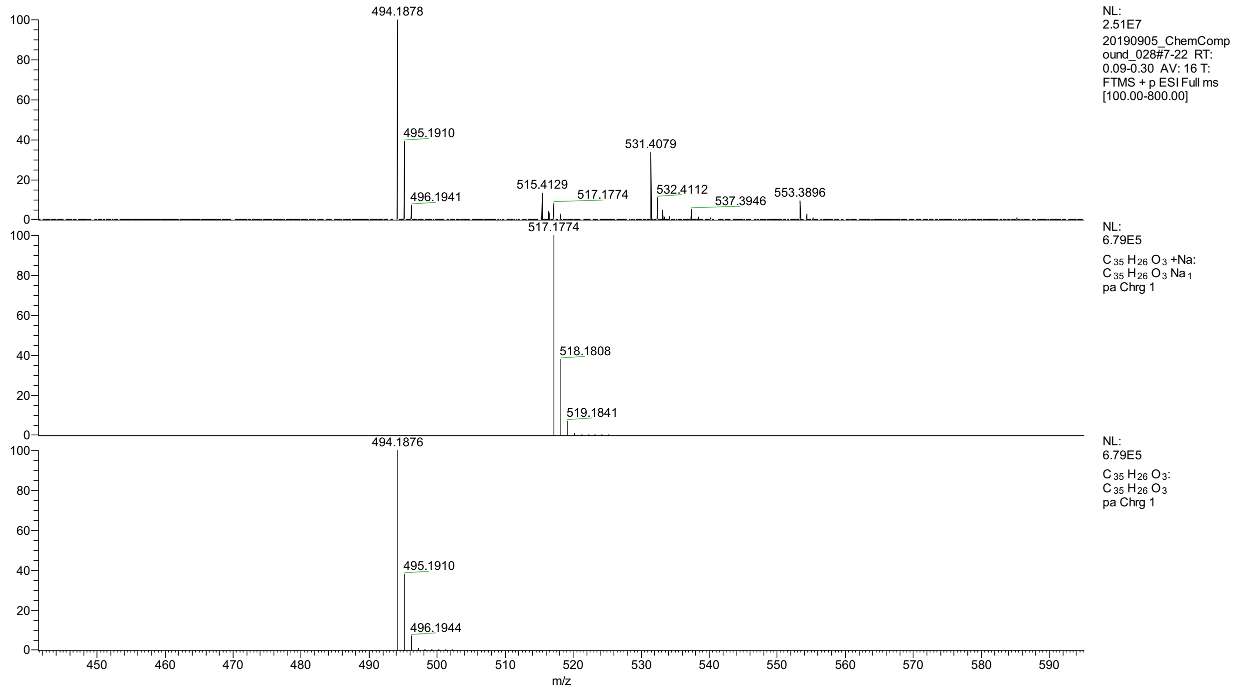

Supplementary Figure 88. HRMS (ESI) of **16**.

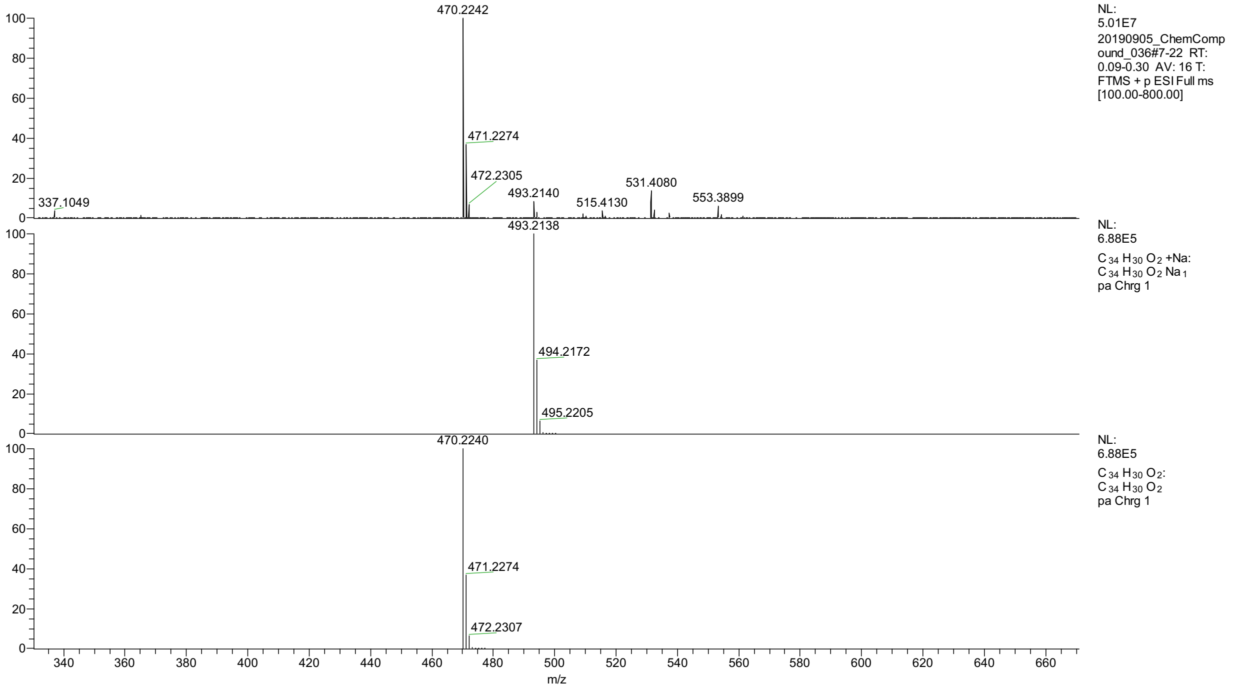

Supplementary Figure 89. HRMS (ESI) of **17**.

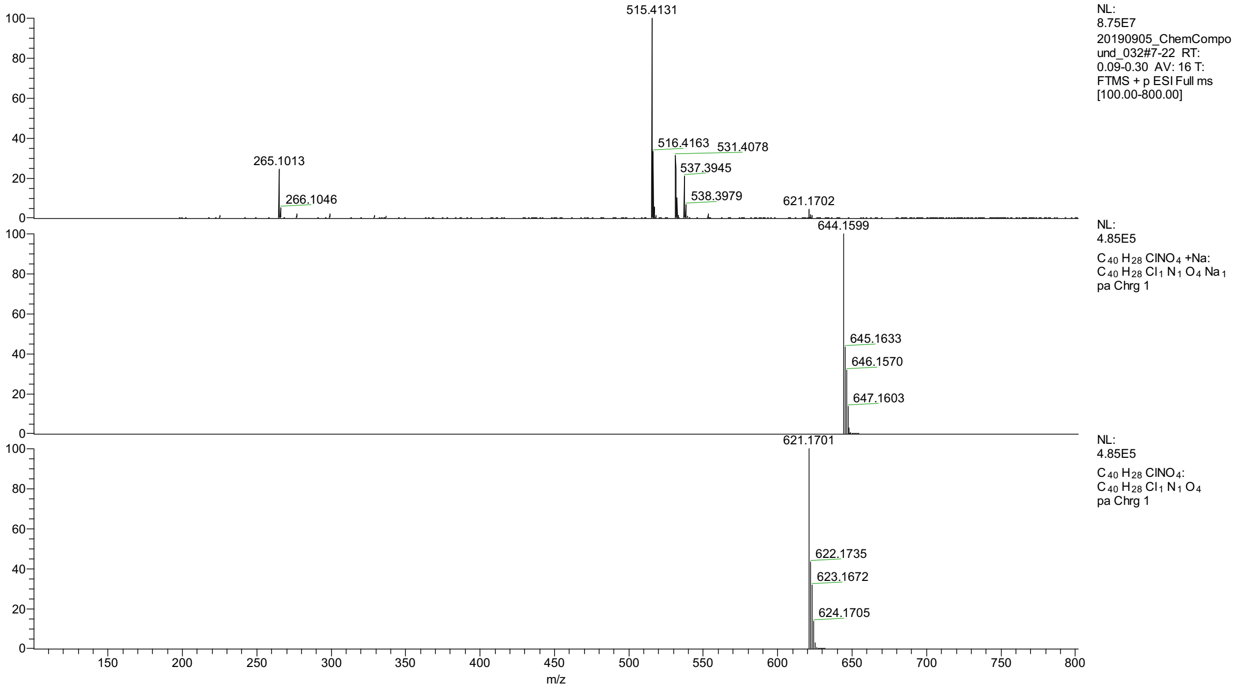

Supplementary Figure 90. HRMS (ESI) of **18**.

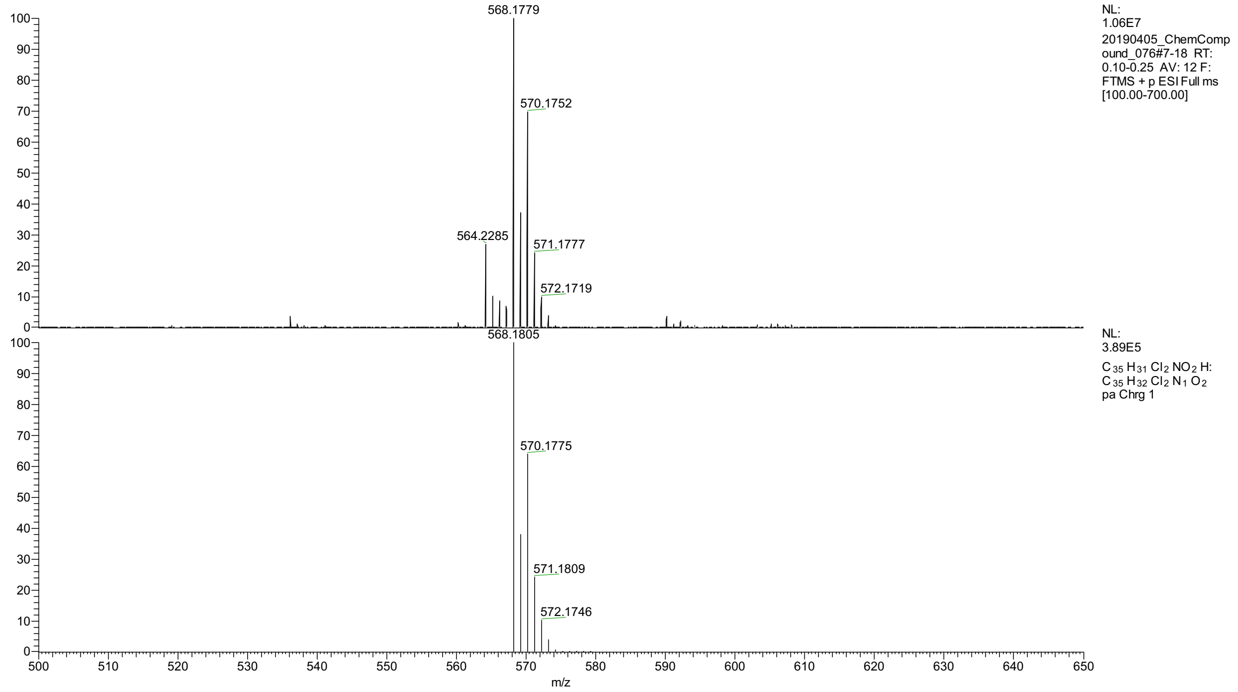

Supplementary Figure 91. HRMS (ESI) of **19**.

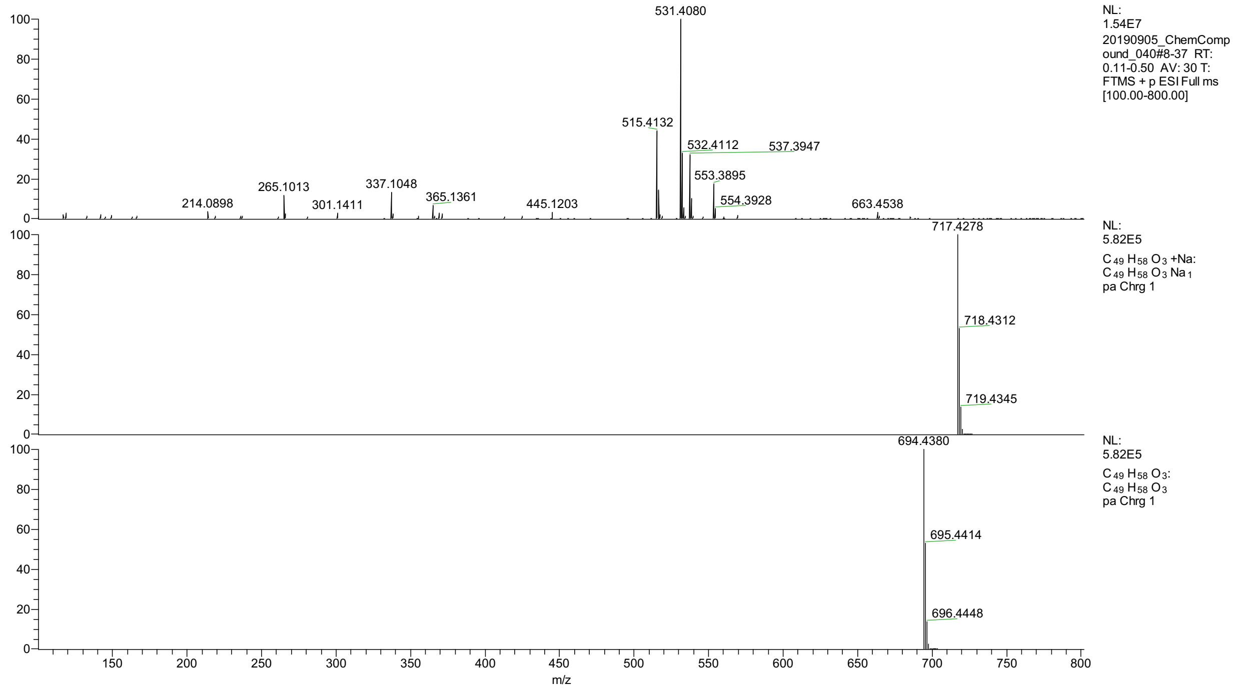

Supplementary Figure 92. HRMS (ESI) of **20**.

REFERENCE

1. Lin, Q. et al. Anticancer Drug Release from a Mesoporous Silica Based Nanophotocage Regulated by Either a One- or Two-Photon Process. *J. Am. Chem. Soc*. **132**,10645−10647 (2010).
2. Mahmood, Z. & Zhao, J. Thiol-Activatable Triplet–Triplet Annihilation Upconversion with Maleimide-Perylene as the Caged Triplet Acceptor/Emitter. *J. Org. Chem*. **81**, 587−594 (2016).
3. Lebedev, A. Y. et al. Dendritic Phosphorescent Probes for Oxygen Imaging in Biological Systems. *ACS Appl. Mater. Interfaces.* **1**, 1292−1304 (2009).
4. Cui, X., Zhao, J., Yang, P. & Sun, J. Zinc (II) tetraphenyltetrabenzoporphyrin complex as triplet photosensitizer for triplet–triplet annihilation upconversion. *Chem. Commun*. **49**, 10221−10223 (2013).
5. Bansal, A. K., Holzer, W., Penzkofer, A. & Tsuboi, T. Absorption and emission spectroscopic characterization of platinum-octaethyl-porphyrin (PtOEP). *Chem. Phys.* **330**, 118−129 (2006).
6. Hofbeck, T. & Yersin, H. The Triplet State of fac-Ir(ppy)$_3$. *Inorg. Chem*. **49**, 9290–9299 (2010).
7. Whited, M. T. et al. Singlet and triplet excitation management in a bichromophoric near-infrared-phosphorescent BODIPY-benzoporphyrin platinum complex. *J. Am. Chem. Soc.* **133**, 88−96 (2011).
8. Cui, X., Zhao, J., Zhou, Y., Ma, J. & Zhao, Y. Reversible photoswitching of triplet–triplet annihilation upconversion using dithienylethene photochromic switches. *J. Am. Chem. Soc.* **136**, 9256–9259 (2014).
9. Ji, S., Wu, W., Wu, W., Guo, H. & Zhao, J. Ruthenium(II) Polyimine Complexes with a Long-Lived ^{3}IL Excited State or a ^{3}MLCT/^{3}IL Equilibrium: Efficient Triplet Sensitizers for Low-Power Upconversion. *Angew. Chem. Int. Ed.* **50**, 1626–1629 (2011).

10. Ma, L., Guo, H., Li, Q., Guo, S. & Zhao, J. Visible light-harvesting cyclometalated Ir(III) complexes as triplet photosensitizers for triplet–triplet annihilation based upconversion. *Dalton Trans*. **41**, 10680−10689 (2012).

11. Goswami, P. P. et al. BODIPY-Derived Photoremovable Protecting Groups Unmasked with Green Light. *J. Am. Chem. Soc*. **137**, 3783−3786 (2015)

12. Jana, A., Ikbal, M. & Singh, N. D. P. Perylen-3-ylmethyl: fluorescent photoremovable protecting group (FPRPG) for carboxylic acids and alcohols. *Tetrahedron*. **68**, 1128−1136 (2012).

13. Atta, S., Ikbal, M., Boda, N., Gaurib, S. S. & Singh, N. D. P. Photoremovable protecting groups as controlled-release device for sex pheromone. *Photochem. Photobiol. Sci.* **12**, 393−403 (2013).

14. Klán, P. et al. Photoremovable Protecting Groups in Chemistry and Biology: Reaction Mechanisms and Efficacy. *Chem. Rev*. **113**, 119–191 (2013).
